# Causal effects of reproductive traits on cognitive function: A two‐sample and multivariable mendelian randomization study

**DOI:** 10.1002/ijgo.70875

**Published:** 2026-02-12

**Authors:** Xia Wang, Yunyun Guo

**Affiliations:** ^1^ The Department of Obstetrics at the Affiliated Hospital of Southwest Medical University Luzhou China; ^2^ The Ageing Epidemiology (AGE) Research Unit School of Public Health, Imperial College London London UK

**Keywords:** causal inference, cognition, mendelian randomization, reproductive traits

## Abstract

**Objective:**

Potential associations between reproductive traits and cognitive function have been discovered; however, the results are inconsistent, and the causalities are unclear. This study utilized Mendelian randomization (MR) analysis to assess the causal impact of reproductive traits on cognitive function.

**Methods:**

We performed two‐sample univariable MR (UVMR) and multivariable MR (MVMR) approach to assess genetic causal associations among reproductive traits and cognitive function. We used the inverse variance weighted (IVW) method to explore the role of reproductive traits on cognitive function in the primary analyses, followed by several sensitivity analyses for robustness of our findings. In addition, we conducted MVMR analyses to assess whether the direct causal effects were independent of two modifiable risk factors: body mass index (BMI) and educational attainment (EA).

**Results:**

UVMR analysis showed a younger age at first sexual intercourse (AFS) was significantly associated with poorer cognitive function across multiple domains. A earlier age at first birth (AFB) was significantly associated with poorer cognitive outcomes, including lower cognitive performance (*β* = 0.116, 95% confidence interval [CI]: 0.087 to 0.146, *P* < 0.001, IVW), reduced fluid intelligence score (*β* = 0.248, 95% CI: 0.186 to 0.309, *P* < 0.001, IVW), and diminished memory performance (*β* = 0.042, 95% CI: 0.012 to 0.071, *P* = 0.006, IVW). After further adjustment for BMI or EA, the associations remained significant. Genetically predicted hormone replacement therapy (HRT) use was associated with cognitive decline, including longer completion time in the Pairs Matching (PM) test and fewer correct and attempted matches in the Symbol Digit Substitution (SDS) test; however, after further adjustment for premature menopause, premature ovarian insufficiency, age at HRT initiation, and duration of use, there was insufficient evidence to support a causal association between HRT use and cognitive decline.

**Conclusion:**

Our UVMR and MVMR analyses provide evidence that earlier AFS and earlier AFB are risk factors for cognitive decline. The protective effect of oral contraceptive pills and fewer number of live births on cognitive function is partly influenced by EA. These findings emphasize the important role of reproductive traits in influencing cognitive function.

## 1 INTRODUCTION

With an aging population, impaired cognitive functioning has gradually become a global public health problem.^1^ Cognitive functioning refers to a wide range of mental activities and processes including executive functioning, attention and learning abilities, memory, and verbal expression.^2^ Impaired cognitive functioning can lead to progressive decline in the above functions, leading to the development of Alzheimer's disease, dementia, depression, bipolar disorder, and even an increased risk of all‐cause mortality.^3^, ^4^ There is strong evidence that there is a gender difference in the prevalence of dementia and cognitive decline, and that this difference varies with age.^5^ A Framingham Heart Study and global estimates of dementia prevalence published in *The Lancet* have shown that women have a higher prevalence of dementia than men, and this difference is projected to persist until 2050.^6^, ^7^ A study that included 21 cohorts on six continents, totaling approximately 30 000 participants who were free of dementia at baseline and of whom 58% were women, showed that the age‐corrected prevalence of dementia was higher in women than in men, despite possible differences in economic levels between countries.^8^ In addition, among participants with cognitive decline, the transition from mild cognitive impairment to dementia was faster in women than in men.^9^

Examining the correlation between sex‐specific reproductive traits and cognitive decline could provide more compelling insights into sex differences affecting cognitive function. However, previous controversies about the impact of female reproductive factors on cognitive performance remain. For example, a national cohort study of 4 500 000 women aged >40 years conducted by Yoo et al. showed that later menarche, earlier menopause and shorter duration of childbearing increased women's risk of dementia and that the use of hormone replacement therapy (HRT) or oral contraceptive pills (OCP) reduced women's dementia risk after more than 5 years of follow‐up.^10^ In contrast, another longitudinal study on aging and dementia came to contradictory conclusions and did not find significant associations between menstrual characteristics, fertility factors, and endogenous or exogenous estrogen levels and dementia risk.^11^

The above studies on female reproductive traits and cognitive function have all been based on observational designs. However, due to the inherent limitations of such studies, it is often difficult to fully account for all reproductive and cognition‐related confounders. Mendelian randomization (MR) analyses can help overcome these limitations by minimizing the effects of confounding and reverse causality, and the use of genetic variants associated with reproductive traits can provide stronger evidence for causal links.^12^ This study used two‐sample MR to investigate the causal effects of female reproductive traits—including age at first sexual intercourse (AFS), age at menarche (AAM), age at menopause (AAMo), age at first birth (AFB), birth weight of the first child, OCP use, HRT, medical abortion, and number of live births—on cognitive function. In addition, we conducted multivariable Mendelian randomization (MVMR) analyses to assess whether the direct causal effects of reproductive traits on cognitive function were independent of two modifiable risk factors: body mass index (BMI, calculated as weight in kilograms divided by the square of height in meters) and educational attainment (EA).

## 2 MATERIALS AND METHODS

### 2.1 Mendelian randomization study design

Our research employed a two‐sample MR framework to investigate the causal effects of reproductive traits—including AFS, AAM, AAMo, AFB, birth weight of the first child, OCP use, HRT, medical abortion, and number of live births—on seven common cognitive‐related outcomes. This MR study used summary data from large‐scale genome‐wide association studies (GWAS), all of which had received ethical approval and participant consent in their original studies. As a result, no additional ethical approval was needed for the present analysis.

**FIGURE 1:**
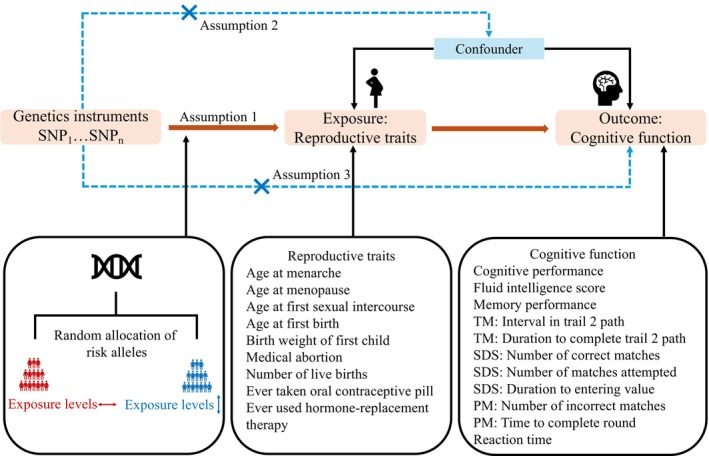
The core assumptions of the Mendelian randomization study. PM, Pairs Matching; SDS, Symbol Digit Substitution; SNP, single nucleotide polymorphism; TM, Trail Making.

Figure 1 illustrates our study design and the three core assumptions of MR^13^: (i) the selected genetic variants (instrumental variables [IVs]) are strongly associated with the reproductive traits; (ii) these genetic variants are not associated with confounders that may influence both reproductive traits and cognitive function; and (iii) the genetic variants affect cognitive outcomes solely through reproductive traits, not via alternative pathways. Ultimately, MR analysis was used to assess the causal relationship between carefully selected genetic variants and cognitive‐related outcomes.

Additionally, given the known associations between reproductive traits and potential risk factors for cognitive function, such as BMI^14^, ^15^ and EA,^16^, ^17^ we conducted a MVMR analysis. This allowed us to adjust for these potential confounders and estimate the direct effects of reproductive traits on cognitive function.

### 2.2 Data sources

#### 2.2.1 Data sources for reproductive traits

Our study utilized publicly available, summary‐level GWAS data. Since 2017, genetic variations related to nine reproductive traits—AFS, AAM, AAMo, AFB, birth weight of the first child, OCP use, HRT, medical abortion, and number of live births—have been investigated in populations of European ancestry. These studies were conducted by several major consortia, including the ReproGen Consortium, UK Biobank, 23andMe, the Human Reproductive Behavior Consortium, and FinnGen.

In the GWAS analyses, AAM, AAMo, AFS, and AFB were treated as continuous variables. Birth weight of the first child and number of live births were analyzed as ordinal categorical variables, while medical abortion, OCP use, and HRT use were treated as binary variables. Sample sizes across these GWAS ranged from 109 910 to 418 758 participants, with the number of SNPs analyzed varying from approximately 9.8 million to over 16.4 million. Further details on the GWAS data for reproductive traits are provided in Table S1.

#### 2.2.2 Data sources for cognitive outcomes

The summary‐level GWAS data for various cognitive outcomes were derived from studies conducted primarily in 2018, focusing on European populations. These genomic studies were carried out by large consortia, incorporating data from 71 quality‐controlled cohorts as well as specialized cohorts such as the Lothian Birth Cohort and the Helsinki Birth Cohort.

The cognitive outcomes examined included cognitive performance, fluid intelligence score (FIS), memory performance, and several task‐based metrics from the Trail Making (TM), Symbol Digit Substitution (SDS), and Pairs Matching (PM) tasks—such as task duration, number of correct or incorrect matches, and time to complete tasks. All outcomes were treated as continuous variables, with the exception of fluid intelligence, which was analyzed as an ordinal categorical variable.

Sample sizes across the studies ranged from 2378 participants (for reaction time) to 462 302 participants (for PM: number of incorrect matches). Most studies analyzed approximately 9.8 million SNPs, with the GWAS on cognitive performance including over 10 million. Detailed information on the GWAS datasets used for cognitive outcomes is provided in Table S1.

#### 2.2.3 Data sources for confounders

To evaluate the direct effects of reproductive traits on cognitive function, we conducted MVMR, accounting for the potential confounding influence of other traits that are genetically correlated with reproductive traits—specifically obesity (using BMI as a proxy) and EA. Summary‐level GWAS data for female BMI were obtained from the UK Biobank and the Avon Longitudinal Study of Parents and Children (ALSPAC), comprising 246 511 female participants of European ancestry.^18^ BMI was treated as a continuous variable in the GWAS analyses.^18^

Data on EA were sourced from the Social Science Genetic Association Consortium (SSGAC), which included 101 069 participants of European ancestry. This study defined two variables: years of education (EduYears), a continuous variable, and college completion (College), a binary variable. We selected the GWAS data corresponding to College, as this measure is likely to be more comparable across different countries, whereas EduYears may reflect greater within‐country individual variation.^19^

### 2.3 Selection of genetic instrumental variables

To satisfy the core assumptions of MR illustrated in Figure 1, we set a genome‐wide significance threshold of *P* < 5 × 10^−8^. For reproductive traits with no genome‐wide significant SNPs—specifically medical abortion and OCP use—we applied a more lenient threshold of *P* < 5 × 10^−6^ to increase SNP inclusion. Linkage disequilibrium (LD) clumping was performed using a threshold of *r* ^*2*^ = 0.001 within 5000 kbp windows, based on the European 1000 Genomes reference panel.^20^ Based on these criteria, the following numbers of independent SNPs were identified: 78 for AFS, 228 for AAM, 120 for AAMo, 78 for AFB, 45 for birth weight of the first child, 26 for OCP use, seven for HRT, 21 for medical abortion, and 11 for number of live births. This ensured that all SNPs included in both univariable MR (UVMR) and MVMR analyses were strongly and independently associated with the corresponding reproductive traits.

Summary statistics were collected from GWAS datasets on reproductive traits and cognitive outcomes, then harmonized to align effect alleles. Palindromic SNPs (e.g., A/T or G/C) were excluded to avoid strand orientation ambiguities. All IVs were aligned to the same DNA strand across both exposure and outcome datasets. To account for potential confounding, SNPs associated with reproductive traits were cross‐referenced in the PhenoScanner database, and those also associated with cognitive outcomes were excluded. Additionally, all retained SNPs were required to have an *F*‐statistic [*F* = (*β*/SE)^2^] > 10 to minimize weak instrument bias.^21^ To further reduce potential horizontal pleiotropy, outlier SNPs were identified and removed using the Mendelian Randomization Pleiotropy RESidual Sum and Outlier (MR‐PRESSO) method. Details of the IV selection process are provided in Figure S1.

### 2.4 Statistical analysis

In the present study, the inverse‐variance weighted (IVW) method was employed as the primary approach for estimating causal effects. To complement and validate the IVW results, we also applied MR‐Egger regression and the weighted median estimator (WME), which provide more robust estimates under different assumptions regarding pleiotropy.^22^, ^23^, ^24^ Given that BMI and EA are potential risk factors for cognitive function, we conducted MVMR analyses to adjust for these covariates and evaluate the direct effects of reproductive traits on cognitive outcomes.^25^, ^26^, ^27^

Sensitivity analyses were conducted to assess the robustness of our findings. These included funnel plot inspection, Cochran's *Q* test, MR‐Egger intercept, Mendelian Randomization Pleiotropy RESidual Sum and Outlier (MR‐PRESSO) analysis, and leave‐one‐out analysis. Funnel plots and Cochran's Q statistics were used to assess heterogeneity among the IVs.^28^ When heterogeneity was detected in the causal estimates across SNPs for any given reproductive trait, we applied a random‐effects IVW model to account for this variability.^28^ To detect horizontal pleiotropy, we used the intercept term from MR‐Egger regression.^29^ Following this, we employed the MR‐PRESSO test to identify and remove outlier SNPs. MR‐PRESSO corrects for horizontal pleiotropy by recalculating causal estimates after excluding these outliers using an outlier‐adjusted model. Finally, we performed a leave‐one‐out analysis to determine whether any single SNP disproportionately influenced the overall causal estimate between reproductive traits and cognitive outcomes.^30^

All analyses were conducted using R software (version 4.4.2), employing the TwoSampleMR (version 0.6.8) and MendelianRandomization (version 0.10.0) packages. To account for multiple testing, the Bonferroni method was applied, setting a corrected significance threshold at 0.0008 (0.05 divided by 63 MR estimates). Associations with *p* values below 0.0008 were considered statistically significant, while those between 0.0008 and 0.05 were classified as suggestive, indicating conventional significance without meeting the stricter Bonferroni‐adjusted criterion.

## 3 RESULTS

### 3.1 Selection of instrumental variables

Based on the IV selection criteria, we identified 78, 228, 120, 78, 45, 26, 7, 21, and 11 SNPs as genetic instruments for the following reproductive traits, respectively: AFS, AAM, AAMo, AFB, birth weight of the first child, OCP, HRT, medical abortion, and number of live births. Full details of all selected IVs included in the analyses are provided in Tables S2–S10. The F‐statistics for the associations between the IVs and reproductive traits ranged from 39.62 to 90.17, indicating strong instruments and little evidence of weak instrument bias.

### 3.2 MR analysis

**FIGURE 2:**
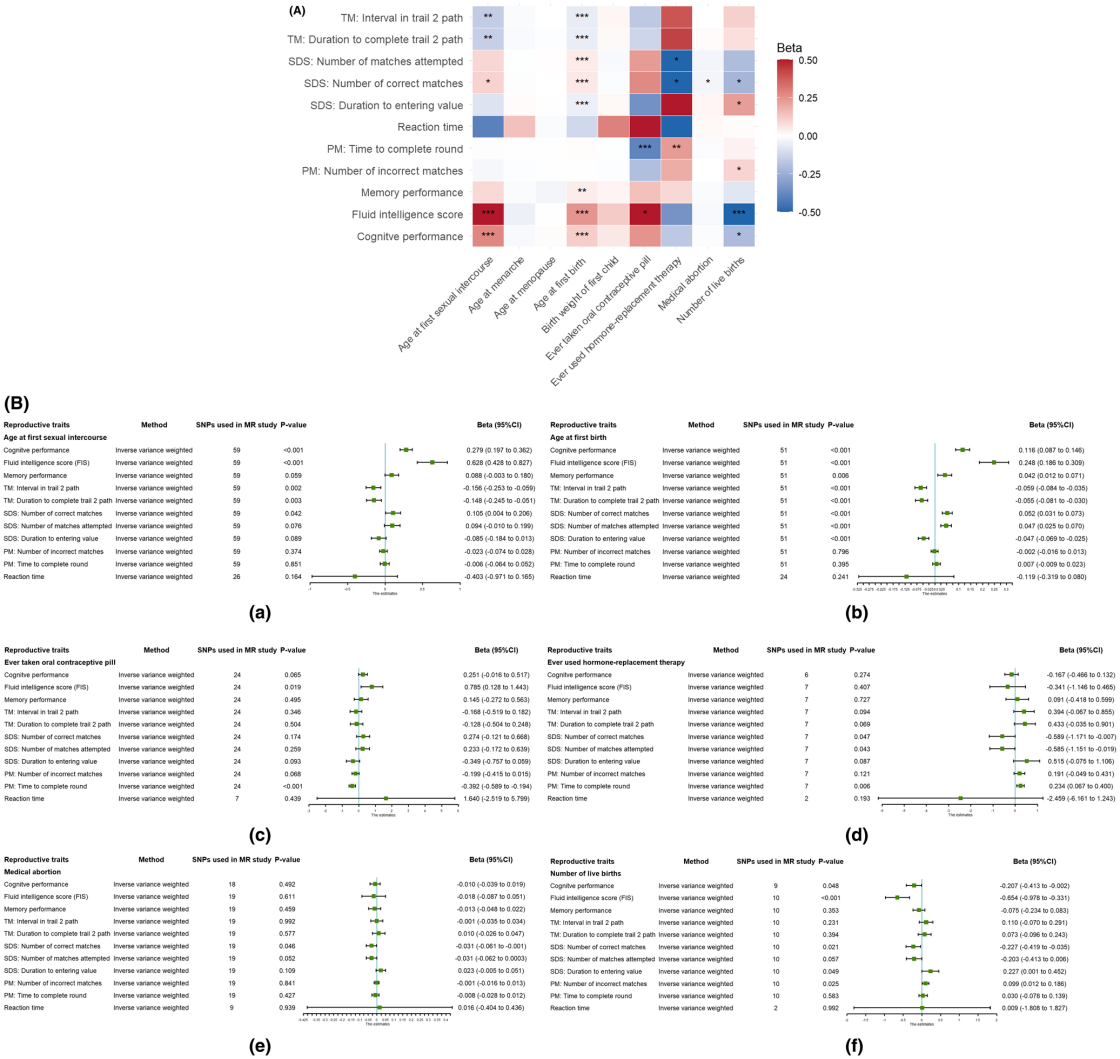
(A) Heat map showing the causal estimates of nine reproductive traits on cognitive function outcomes in the primary analyses with IVW. (B) Forest plot of Mendelian randomization analyses for the relevance of (a) age at first sexual intercourse, (b) age at first birth, (c) oral contraceptive pills, (d) hormone replacement therapy, (e) medical abortion, and (f) number of live births with cognitive‐related outcomes. CI, confidence interval; IVW, inverse variance weighted; MR, Mendelian randomization; PM, Pairs Matching; SDS, Symbol Digit Substitution; SNP, single nucleotide polymorphism; TM, Trail Making.

We evaluated the causal effects of nine reproductive traits on seven cognitive function outcomes. Figure 2A summarizes the direction and magnitude of the associations between each reproductive trait and cognitive outcome. The results revealed that six reproductive traits—AFS, AFB, OCP use, HRT, medical abortion, and number of live births—were causally associated with future cognitive decline. Figure 2B presents detailed results for significant associations related to reproductive traits.

Univariable MR analysis provided credible evidence that a younger AFS was significantly associated with poorer cognitive function across multiple domains. Specifically, earlier AFS was linked to lower cognitive performance (*β* = 0.279, 95% confidence interval [CI]: 0.197 to 0.362, *P* < 0.001, IVW), lower FIS (*β* = 0.628, 95% CI: 0.428–0.827, *P* < 0.001, IVW), fewer correct matches on the SDS test (*β* = 0.105, 95% CI: 0.004–0.206, *P* = 0.042, IVW), and worse performance on the TM test, including longer interval times (*β* = −0.156, 95% CI: −0.253 to −0.059, *P* = 0.002, IVW) and longer completion durations for the Trail 2 path (*β* = −0.148, 95% CI: −0.245 to −0.051, *P* = 0.003, IVW).

Likewise, a younger AFB was significantly associated with poorer cognitive outcomes, including lower cognitive performance (*β* = 0.116, 95% CI: 0.087–0.146, *P* < 0.001, IVW), reduced FIS (*β* = 0.248, 95% CI: 0.186–0.309, *P* < 0.001, IVW), and diminished memory performance (*β* = 0.042, 95% CI: 0.012–0.071, *P* = 0.006, IVW). Younger AFB was also consistently associated with poorer performance across all subcomponents of the SDS and TM tests. On the SDS test, earlier AFB was linked to fewer correct matches (*β* = 0.052, 95% CI: 0.031–0.073, *P* < 0.001, IVW), fewer attempted matches (*β* = 0.047, 95% CI: 0.025–0.070, *P* < 0.001, IVW), and slower input speed (*β* = −0.047, 95% CI: −0.069 to −0.025, *P* < 0.001, IVW). In the TM test, it was associated with longer interval times (*β* = −0.059, 95% CI: −0.084 to −0.035, *P* < 0.001, IVW) and increased duration to complete the Trail 2 path (*β* = −0.055, 95% CI: −0.081 to −0.030, *P* < 0.001, IVW).

A greater number of live births was also associated with worse cognitive outcomes. Specifically, it was linked to lower cognitive performance (*β* = −0.207, 95% CI: −0.413 to −0.002, *P* = 0.048, IVW) and reduced FIS (*β* = −0.654, 95% CI: −0.978 to −0.331, *P* < 0.001, IVW). Furthermore, it was associated with poorer outcomes on the PM test, as reflected by an increased number of incorrect matches (*β* = 0.099, 95% CI: 0.012–0.186, *P* = 0.025, IVW), and on the SDS test, including fewer correct matches (*β* = −0.227, 95% CI: −0.419 to −0.035, *P* = 0.021, IVW) and longer duration to enter a value (*β* = 0.227, 95% CI: 0.001–0.452, *P* = 0.049, IVW).

OCP use was positively associated with cognitive function, including higher FIS (*β* = 0.785, 95% CI: 0.128–1.443, *P* = 0.019, IVW) and shorter completion time in the PM test (*β* = −0.392, 95% CI: −0.589 to −0.194, *P* < 0.001, IVW). In contrast, HRT use was associated with poorer cognitive performance, including longer time to complete rounds in the PM test (*β* = 0.234, 95% CI: 0.067–0.400, *P* = 0.006, IVW), fewer correct matches in the SDS test (*β* = −0.589, 95% CI: −1.171 to −0.007, *P* = 0.047, IVW), and fewer attempted matches (*β* = −0.585, 95% CI: −1.151 to −0.019, *P* = 0.043, IVW).

There was also evidence of an inverse causal relationship between medical abortion and performance on the SDS test, specifically in the number of correct matches (*β* = −0.031, 95% CI: −0.061 to −0.001, *P* = 0.046, IVW). The MR‐Egger and WME methods yielded similar directions of effect, although not all estimates reached statistical significance (Figure S2). No significant associations were found between AAM, AAMo, or birth weight of first child and any of the cognition‐related outcomes.

### 3.3 Sensitivity analysis

In our sensitivity analyses, some heterogeneity was detected in the cognitive function outcomes, as indicated by funnel plot visualizations and Cochran's Q test (Figure S3 and Table S11). To account for this, random‐effects IVW models were applied to mitigate the influence of heterogeneity on the causal estimates. The MR‐Egger regression intercept analyses indicated no evidence of horizontal pleiotropy (Table S12). After applying the outlier‐corrected method in MR‐PRESSO and removing outlier IVs, we observed further evidence that a earlier AFS was associated with poorer memory performance and reduced accuracy in the SDS subtest (number of matches attempted). We also found additional evidence that a higher number of live births was linked to diminished cognitive function in several domains, including the interval in the Trail 2 path (TM), the duration to enter a value (SDS), and the number of incorrect matches in the PM task (Table S13). Furthermore, the leave‐one‐out analyses confirmed that the causal estimates of reproductive traits on cognitive function were not driven by any single SNP (Figure S4).

### 3.4 Multivariable mendelian randomization (MVMR)

**FIGURE 3:**
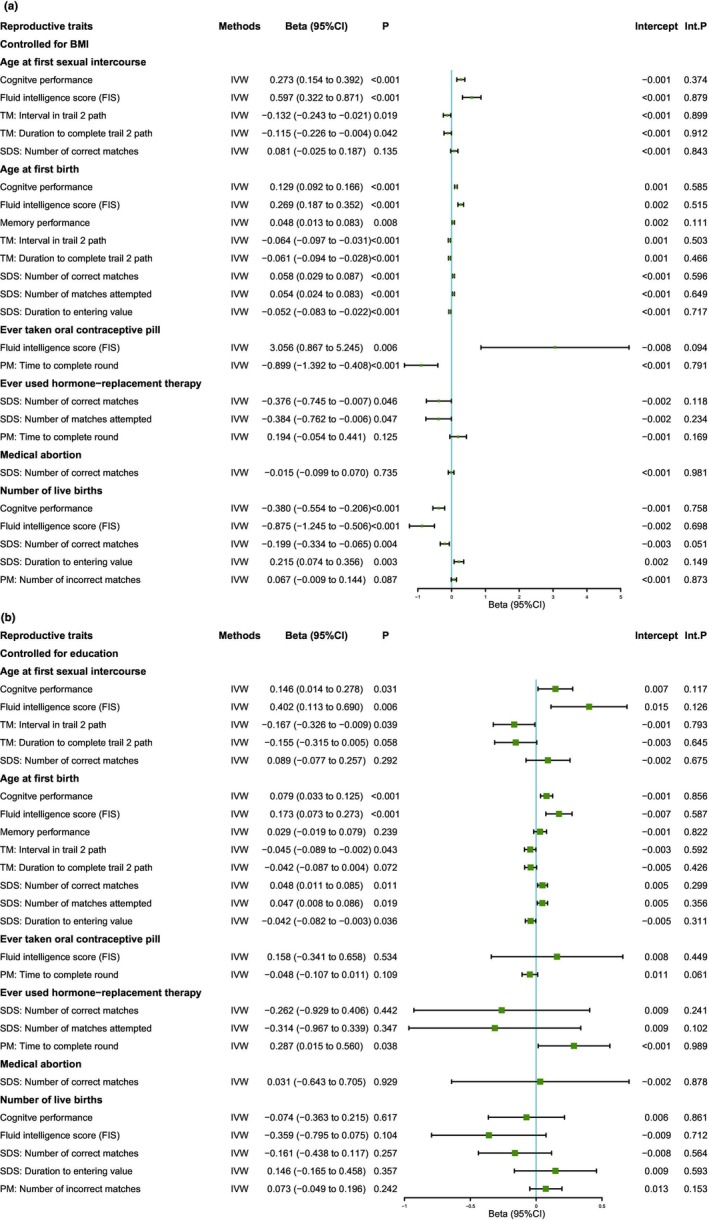
Multivariable Mendelian randomization analyses of statistically significant reproductive traits and cognitive function tests in the univariable Mendelian randomization (UVMR), after adjustment for (a) body mass index (BMI) and (b) educational attainment (EA). P estimates the causal effect of reproductive traits on cognitive function. A small *P* value (<0.05) suggests that the estimated causal effect is statistically significant. Int.P refers to the *P* values derived from the Egger intercepts. MR‐Egger intercept test detects horizontal pleiotropy. A *P* value >0.05 indicates no significant pleiotropy. CI, confidence interval; IVW, inverse variance weighted; PM, Pairs Matching; SDS, Symbol Digit Substitution; SNP, single nucleotide polymorphism; TM, Trail Making.

In the MVMR analyses, additional genetic variants significantly associated with BMI and EA were incorporated alongside each woman's reproductive trait. After adjusting for BMI, older AFS remained significantly associated with better cognitive performance (*β* = 0.273, 95% CI: 0.154–0.392, *P* < 0.001, IVW), higher FIS (*β* = 0.597, 95% CI: 0.322–0.871, *P* < 0.001, IVW), and improved performance on the TM test (interval in Trail 2 path: *β* = −0.132, 95% CI: −0.243 to −0.021, *P* = 0.019, IVW; duration to complete Trail 2 path: *β* = −0.115, 95% CI: −0.226 to −0.004, *P* = 0.042, IVW) (Figure 3a).

The causal effect of older AFB also remained robust across multiple cognitive domains, including cognitive performance (*β* = 0.129, 95% CI: 0.092–0.166, *P* < 0.001, IVW), FIS (*β* = 0.269, 95% CI: 0.187–0.352, *P* < 0.001, IVW), memory performance (*β* = 0.048, 95% CI: 0.013–0.083, *P* = 0.008, IVW), TM test (interval: *β* = −0.064, 95% CI: −0.097 to −0.031, *P* < 0.001; duration: *β* = −0.061, 95% CI: −0.094 to −0.028, *P* < 0.001), and SDS test (correct matches: *β* = 0.058, 95% CI: 0.029–0.087, *P* < 0.001; matches attempted: *β* = 0.054, 95% CI: 0.024–0.083, *P* < 0.001; duration to enter value: *β* = −0.052, 95% CI: −0.083 to −0.022, *P* < 0.001) (Figure 3a).

In addition, OCP use was significantly associated with better FIS (*β* = 3.056, 95% CI: 0.867–5.245, *P* = 0.006, IVW) and shorter completion time in the PM test (*β* = −0.899, 95% CI: −1.392 to −0.408, *P* < 0.001, IVW). HRT use was associated with lower SDS performance, including fewer correct matches (*β* = −0.376, 95% CI: −0.745 to −0.007, *P* = 0.046, IVW) and fewer matches attempted (*Β* = −0.384, 95% CI: −0.762 to −0.006, *P* = 0.047, IVW). Number of live births also demonstrated a consistent negative causal effect on cognition after adjusting for BMI, including cognitive performance (*β* = −0.380, 95% CI: −0.554 to −0.206, *P* < 0.001), FIS (*β* = −0.875, 95% CI: −1.245 to −0.506, *P* < 0.001), and SDS test performance (correct matches: *β* = −0.199, 95% CI: −0.334 to −0.065, *P* = 0.004; duration to enter value: *β* = 0.215, 95% CI: 0.074–0.356, *P* = 0.003). However, the previously observed effect of medical abortion on SDS test performance lost statistical significance after adjusting for BMI (Figure 3a).

After further adjustment for EA, the association between older AFS and improved cognition remained significant, including effects on cognitive performance (*β* = 0.146, 95% CI: 0.014–0.278, *P* = 0.031), FIS (*β* = 0.402, 95% CI: 0.113–0.690, *P* = 0.006), and TM test interval (*β* = −0.167, 95% CI: −0.326 to −0.009, *P* = 0.039). Genetically predicted older AFB was also positively associated with cognitive performance (*β* = 0.079, 95% CI: 0.033–0.125, *P* < 0.001), FIS (*β* = 0.173, 95% CI: 0.073–0.273, *P* < 0.001), TM test interval (*β* = −0.045, 95% CI: −0.089 to −0.002, *P* = 0.043), and SDS test outcomes (correct matches: *β* = 0.048, 95% CI: 0.011–0.085, *P* = 0.011; matches attempted: *β* = 0.047, 95% CI: 0.008–0.086, *P* = 0.019; duration: *β* = −0.042, 95% CI: −0.082 to −0.003, *P* = 0.036). The negative effect of HRT on PM test performance also remained (*β* = 0.287, 95% CI: 0.015–0.560, *P* = 0.038). In contrast, no significant associations were detected between OCP use, medical abortion, or number of live births and any cognitive function measures after accounting for EA (Figure 3b). We additionally conducted sensitivity analyses. The intercept from MR‐Egger in the multivariable MR did not indicate evidence of horizontal pleiotropy. Additionally, for HRT, we further adjusted for premature menopause, premature ovarian insufficiency (POI), age at HRT initiation and duration of use. The results indicate insufficient evidence to support a causal relationship between HRT use and cognitive decline (Figure S5). To further address potential postnatal influences, we conducted additional MVMR analyses adjusting for breastfeeding in the associations of age at first birth and number of live births with cognitive outcomes. The overall patterns of association remained broadly consistent after adjustment, suggesting that the observed findings are not solely attributable to breastfeeding‐related effects (Figure S6). We have added an additional schematic figure (Figure S7), used together with Figure 1, to summarize the study design and key findings.

## 4 DISCUSSION

In this study, we conducted two‐sample MR analyses for the first time to evaluate the causal effects of women's reproductive traits—including AFS, AAM, AAMo, AFB, birth weight of the first child, OCP use, HRT, medical abortion, and number of live births—on cognitive function. In UVMR analyses, we found that genetically predicted later AFS and AFB, as well as fewer live births, were associated with better cognitive outcomes, while OCP use was identified as a protective factor. In contrast, HRT use and medical abortion showed adverse causal effects on cognitive function. These associations remained significant for AFS, AFB, OCP use, HRT use, and number of live births after adjusting for BMI. Furthermore, the effects of AFS, AFB, and HRT on cognitive function persisted after accounting for EA. However, the adverse effect of medical abortion was no longer significant after adjusting for either BMI or EA.

Previous observational studies on the association between reproductive traits and cognitive function have reported mixed results. In line with our findings, some studies found that later AFS, AFB, and lower number of live births were protective factors for cognitive function. A population‐based cohort study in Denmark, covering more than 4 million people, found that older first‐time mothers had a slightly lower overall risk of dementia compared with women aged 25–29 who had their first child. At the same time, men and women who became parents at less than 20 years of age had the greatest overall risk of dementia compared with other age groups.^31^ A recent cross‐sectional survey that included 1000 women over the age of 60 suggested that the later the AFB, the better the participants' performance in situational and working memory, verbal fluency, sustained attention, executive functioning, cognitive flexibility, and overall cognitive functioning.^32^ Analyzing pooled data on 3500 women from two cohort studies, the Consortium for the Study of Cohorts of Memory International (COSMIC) found that among women without dementia at baseline, women with ≥5 full‐term pregnancies had a risk of AD that was approximately 1.7 times higher than that of women with one to four full‐term pregnancies, and that cognitive scores were poorer in women with multiple pregnancies than in women with one to four full‐term pregnancies.^33^ Another study, COSMIC, which pooled 11 cohorts of nearly 15 000 community‐dwelling older women, showed that women who had five or more births had a 47% increased dementia risk compared with those who had only one birth.^34^ A prospective cohort study by Gong et al. that included more than 500 000 participants with a median follow‐up of 11.8 years found a 14% increased dementia risk in women with four or more children compared with women with two children.^35^ Traditional observational studies struggle to capture causal relationships between reproductive traits and cognitive function. Recently, a Mendelian randomization analysis by Wang et al. suggested that genetic predictions of earlier AAM, AAMo, and AFB were significantly associated with AD risk.^36^ However, Oppenheimer et al., using an AD dataset excluding UK Biobank samples to minimize sample overlap, did not observe a significant causal association between AAM, AAMo, and AD.^37^ Compared to the study by Wang et al., our research incorporated additional key exposure factors, such as AFS, birth weight of the first child, OCP use, HRT, medical abortion, and number of live births. Moreover, while prior studies primarily focused on AD as an outcome measure,^36^, ^37^ our research comprehensively assessed cognitive function using seven cognitive tests and their subtests. This broader phenotypic characterization may be more sensitive to detecting early cognitive changes, partially explaining the discrepancy between our findings and those of previous studies.

Estrogen exposure may be a potential mechanism to explain the causal effect of AFS, AFB, and lower number of live births on cognitive function. Based on the results of the present study, we hypothesized that later AFS and AFB may prolong the protective effects of estrogen to some extent. Estrogen levels usually peak just before ovulation. Earlier childbearing allows women to experience hormonal fluctuations earlier, shortening the period of stable high estrogen exposure; whereas later childbearing prolongs this exposure period and may contribute to cognitive function.^38^, ^39^ At the same time, we hypothesized that fewer births represent greater lifetime estrogen exposure. This is because there is evidence that childbearing “resets” ovarian function in the non‐pregnant state, resulting in lower estrogen concentrations in fertile women than in non‐pregnant women.^40^ In addition, two studies have suggested that estrogen concentrations are negatively correlated with the number of children.^40^, ^41^ Estrogen deficiency is considered a risk factor for cognitive dysfunction. Previous studies have demonstrated the neuroprotective effect of estrogen against β‐amyloid‐induced neurotoxicity in animal tests and in vitro models.^42^, ^43^ Estrogen can enhance and maintain cognitive function by stimulating microglia‐associated Aβ clearance, stimulating the growth of neural progenitor cells in the subgranular zone of the hippocampal dentate gyrus, increasing the number of dendritic spines and enhancing synaptic connections.^43^, ^44^

Our study found that, after adjusting for EA, the associations between the number of live births and OCP use with cognitive function were no longer statistically significant. This finding suggests that the impact of the number of live births on cognitive function may not be solely attributable to pregnancy‐related hormonal changes, but may instead be more strongly influenced by social factors. Higher levels of education are often associated with increased income and higher socioeconomic status (SES).^45^ Women with lower EA and SES tend to have higher rates of unintended pregnancies, earlier age at first pregnancy, and a greater number of children. Early childbearing, in turn, can limit educational opportunities.^46^, ^47^ Even after starting a family, factors such as educational level, employment status, and material resources can continue to influence reproductive choices, including the number of children.^48^ A study by Kim et al. suggested an inverse association between parental education level and the number of children.^49^ On the other hand, cognitive reserve theory suggests that education promotes cognitive functioning and reduces the risk of cognitive impairment, and the protective effect of education on cognitive health is believed to persist into later life.^50^, ^51^, ^52^

Previous findings on the effects of OCP on cognitive functioning have been inconsistent. Gravelsins et al. showed that OCP‐using women had significantly higher active control indices in reaction time than non‐users when performing a sustained performance task, suggesting that they performed better in maintaining goal‐related information.^53^ However, it has also been found that OCP use may be associated with poorer mental arithmetic accuracy and reduced verbal fluency.^54^, ^55^ The most likely reason for the differences in the results of previous studies is the different types of OCPs. Despite the fact that most are combination preparations containing estrogens, androgens and progestins, there are still significant differences in hormone types and dosages between the different drugs. Second, due to the wide variety of OCPs, this also increased the heterogeneity among users. Another possible influencing factor is the generally small sample size. The number of oral contraceptive users in many studies ranged from only 10 to 60.^53^, ^54^, ^55^ Small sample studies are prone to type I errors, such as overestimating effect sizes, and type II errors, which result in failure to identify effects that are truly present in the population.^56^ Both types of errors undermine the replicability of study results.^57^ Findings on the timing of OCP use are also divided. Some studies have found that use of OCP for no more than 5 years is associated with a reduced risk of cognitive impairment,^58^ whereas others have shown superior cognitive performance in long‐term users.^59^, ^60^ In addition, studies generally lack detailed information on the onset of hormone use and history of prior use.

HRT is commonly used in peri‐ and postmenopausal women to supplement the body's decrease in estrogen, thereby alleviating abnormal vasodilatory responses and other menopause‐related symptoms.^61^ The Women's Health Initiative Memory Study (WHIMS), which enrolled 4000 female participants aged >65 years, found after 4 years that HRT exposure resulted in a two‐fold increase in the risk of a dementia diagnosis, demonstrating exacerbated cognitive decline.^62^, ^63^ However this finding contradicts the favorable effects of estrogen on cognitive function. The “critical window hypothesis” can be used to explain this puzzling paradoxical result. This hypothesis suggests that the effectiveness of HRT is critically dependent on the time window in which it is administered shortly after the menopausal transition. Since women enter menopause at an average of 51 years of age, female participants in WHIMS may have had declining estrogen levels for more than a decade at the time of enrolment.^64^ Our UVMR analysis suggests that HRT appears to be associated with cognitive decline. However, in this study, HRT was defined in the genetic analysis as a binary lifetime exposure factor, lacking information on underlying clinical conditions, age at HRT initiation, and duration of use. Notably, genetically predicted HRT use may represent underlying reproductive aging processes, such as early menopause or POI, both characterized by prolonged estrogen deficiency. To test this hypothesis, we further conducted MVMR analyses adjusted for early menopause, POI, age at HRT initiation, and duration of use. In these adjusted models, the association between genetically predicted HRT use and cognitive outcomes ceased to be significant. This suggests that the association observed in the UVMR is more likely to reflect cognitive consequences arising from estrogen deficiency associated with early reproductive aging, rather than adverse effects of HRT use itself. Therefore, in conjunction with the evidence previously presented in WHIMS, our findings should not be interpreted as evidence that HRT itself causes cognitive decline, but rather underscore the importance of the timing of HRT use.

Our study utilized MR analyses to, to our knowledge, comprehensively investigate the potential causal associations between nine female reproductive traits and performance across a wide range of cognitive function tests. This approach has key advantages in minimizing confounding and mitigating reverse causation inherent in traditional observational studies. Importantly, the use of genetic instruments reflects lifelong exposure, thereby strengthening causal inference in the investigation of reproductive traits and cognitive function. In addition, we considered the role of BMI and EA in the link between reproductive factors and cognitive function. Consistent evidence between UVMR and MVMR supports genetically predicted causal negative effects of earlier AFS and earlier AFB on cognitive function. However, we should interpret our findings with caution. First, the pooled data on female reproductive traits included only females, whereas cognitive functions were tested in both males and females. Therefore, if there are differences in the effects of genetic variants on sex, this may bias our results. However, our analyses using SNPs for AFS, AFB, birth weight of first child, and number of live births that included pooled data for both males and females were consistent with the main analysis, suggesting that this bias had a small effect on our results. Second, there was partial overlap between the data samples for reproductive traits and cognitive function outcomes. The ideal situation for two‐sample MR is that there is no overlap between exposures and outcomes. At the same time, the low threshold for selecting SNPs as genetic tools for medical abortion and OCP may have biased the results. However, complete non‐overlap is difficult to achieve using publicly available aggregated data. The robustness of the genetic tools (all means *F* > 10) suggests that no considerable bias will occur. Further, based on Burgess's simulations, we calculate expected bias to be no more than 0.001 and class I error to be no more than 0.05.^65^ Third, we could not completely rule out the effect of genetic pleiotropy on the results, but we performed the MR‐Egger intercept test and MVMR within BMI and EA to account for the high genetic correlations and to obtain evidence of independent effects of reproductive factors on cognitive function. Fourth, we used summary‐level data from participants of European ancestry. The differential association of multiple births with the risk of cognitive decline across geographic regions may be due to the different sensitivities of the hormonal and vascular systems to the effects of pregnancy or childbirth across ethnic and racial groups. For example, one study found that the number of births affected estradiol levels during pregnancy in white women but not in Asian women.^66^ Thus our results cannot be generalized to other races. Fifth, the MR design examines the cumulative effects of reproductive traits on cognitive function over a lifetime, and it is difficult to capture short‐term effects. Sixth, the associations between the use of HRT or OCP and cognitive function should be interpreted with caution, as these exposures were defined as binary lifetime measures, and information on age at initiation, duration of use, timing of cessation, indication for use, and reasons for discontinuation was not available. Finally, MR analyses are based on linear assumptions and therefore do not allow inference regarding optimal ranges for age at first birth or number of live births. Consequently, our findings should not be interpreted as evidence that later childbearing improves cognitive outcomes or that higher parity is inherently detrimental. In addition, although sensitivity analyses adjusting for breastfeeding were performed, individual‐level longitudinal data are required to explore potential non‐linear relationships and to more comprehensively disentangle postnatal and social factors influencing cognitive aging.

## 5 CONCLUSIONS

Our UVMR and MVMR analyses provide evidence that earlier AFS and earlier AFB are risk factors for cognitive decline. The protective effect of OCP and fewer number of live births on cognitive function is partly influenced by EA. These findings emphasize the important role of reproductive traits in influencing cognitive function.

## AUTHOR CONTRIBUTIONS

YG designed the study, performed the statistical analysis, and wrote the manuscript. XW contributed to professional advice on mechanisms linking reproductive traits and cognitive function. All authors provided critical feedback on the multiple drafts of the manuscript.

## FUNDING INFORMATION

The study was not funded by any organization.

## CONFLICT OF INTEREST STATEMENT

The authors have no conflicts of interest to declare.

## Supporting information


Data S1.

## Data Availability

The GWAS summary statistics of reproductive traits used in this MR study are available in OpenGWAS(https://gwas.mrcieu.ac.uk/datasets/ukb‐b‐10817/), (https://gwas.mrcieu.ac.uk/datasets/ukb‐b‐589/), (https://gwas.mrcieu.ac.uk/datasets/ukb‐b‐5620/), and (https://gwas.mrcieu.ac.uk/datasets/ukb‐b‐12417/). Detailed information for reproductive traits and cognitive function outcomes refer to Table S1.
