# Supplementary material for: Causal effects of reproductive traits on cognitive function: A two‐sample and multivariable mendelian randomization study
Source: Int J Gynaecol Obstet. 2026 Feb 12;174(1):424–35. doi: 10.1002/ijgo.70875 (PMC13278646; doi:10.1002/ijgo.70875)
Supplement: Supplementary file 1 — Data S1. [file IJGO-174-424-s001.pdf]

Supplementary materials

Table S1. Description of the genetic instruments associated with reproductive traits and cognitive function tests

Table S2. Genetic variants used as instrumental variables for age at menarche.

Table S3. Genetic variants used as instrumental variables for age at menopause.

Table S4. Genetic variants used as instrumental variables for age at first sexual intercourse.

Table S5. Genetic variants used as instrumental variables for age at first birth.

Table S6. Genetic variants used as instrumental variables for birth weight of first child .

Table S7. Genetic variants used as instrumental variables for number of live births.

Table S8. Genetic variants used as instrumental variables for medical abortion.

Table S9. Genetic variants used as instrumental variables for oral contraceptive pills.

Table S10. Genetic variants used as instrumental variables for ever used hormone-replacement therapy.

Table S11. Cochran's Q tests for heterogeneity from MR-IVW and MR-Egger analyses of reproductive traits and cognitive function.

Table S12. MR-Egger intercept tests for horizontal pleiotropy from MR analysis of reproductive traits and cognitive function.

Table S13. The results of MR analyses using the Outlier-corrected method in MR-PRESSO with the results of the original MR analyses.

Figure S1 Study workflow IV selection for analysis of the effect of reproductive traits on cognitive function

Figure S2. Scatter plot of SNPs impacts on reproductive traits and cognitive function.

Figure S3. Funnel plot for cogitive function regarding reproductive traits.

Figure S4. Forest plots depicting the results of sensitivity analyses using a leave-one-out approach to assess the influence of reproductive traits on cognitive function.

Figure S5. MVMR analyses of statistically significant HRT use and cognitive function tests in the UVMR, after adjustment for early menopause, premature ovarian insufficiency, age at HRT initiation and duration of use.

Figure S6. MVMR analyses of statistically significant age at first birth/number of live births and cognitive function tests in the UVMR, after adjustment for total breastfeeding duration.

Figure S7. Study workflow and summary of key results from Mendelian randomization analyses

Table S1. Description of the genetic instruments associated with reproductive traits and cognitive function tests

Phenotypes	Sample size	Number of SNPs	Population	Year	GWAS ID	Consortium (Source)	Phenotype Definition	Data Type	Notes	Similarities	Key Differences / Notes
**REPRODUCTIVE TRAITS**											
Age at menarche	252,514	10,562,208	European	2017	ieu-b-5106	ReproGen Consortium	Self-reported age (years) at first menstrual bleeding	Continuous	In the Reproductive Genetics (ReproGen) Consortium GWAS, age at menarche is defined as the self-reported age in whole years at first menstrual bleeding (onset of menstruation) in female participants. Ages reported <9 or >17 years were excluded to improve accuracy/consistency across cohorts.	Self-reported; measured in years; treated as a continuous trait	Minor differences in exclusion criteria (e.g. implausible ages) and cohort-specific quality control

						UK Biobank	Self-reported age at first menstruation	Continuous	In UK Biobank studies, age at menarche is also self-reported by female participants, usually collected via questionnaire at recruitment. Mean ages in UK Biobank datasets are typically around ~12.9–13.0		
						23andMe Studies	Self-reported age at menarche	Continuous	23andMe contributes summary statistics for age at menarche from its research cohort based on self-reported age at first menstruation (in whole years).		

Age at menopause	143,819	9,851,867	European	2018	ukb-b-17422	MRC-IEU	Age at last menstrual period as captured in the UK Biobank data and analysed through the MRC-IEU GWAS pipeline. The GWAS summary statistics reflect genetic associations with the age (in standard deviation units) at which a woman experiences her last menstrual period, which is used as the proxy for menopause timing in this dataset.	Continuous	This phenotype (last menstrual period) is often used as a measure of age at natural menopause in genetic epidemiology studies	Self-reported; continuous trait	Some cohorts exclude surgical menopause or HRT users, while others use last menstrual period as a proxy
Age at first sexual intercourse	214,547	16,426,473	European	2021	ebi-a-GCST90000045	UK Biobank	Self-reported age (in whole years) at first sexual intercourse collected via baseline touchscreen questionnaire.	Continuous	Participants who reported ever having had sexual intercourse; widely used in genetic and epidemiological studies.	Self-reported; continuous trait; harmonised across cohorts	Questionnaire wording and age reporting may vary between cohorts
						Human Reproductive Behaviour Consortium	Self-reported age at first sexual intercourse harmonised across multiple cohorts.	Continuous	Cohort-specific quality control applied to ensure consistency across studies; used in large-scale GWAS		

									meta-analyses of reproductive behaviours.		
Age at first birth	418,758	10,766,720	European	2021	ebi-a-GCST90000048	UK Biobank	Age at first birth is defined as the self-reported age (in whole years) at which a female participant gave birth to her first child. This information was collected via the baseline touchscreen questionnaire.	Continuous	Analyses include women who reported at least one live birth.	Self-reported; continuous trait; restricted to women with ≥1 live birth	Differences in handling of assisted reproduction and parity restrictions
						Human Reproductive Behaviour Consortium	Age at first birth is defined as the self-reported age at which a woman gave birth to her first child, harmonised across multiple contributing cohorts.	Continuous	Analyses focus on natural reproductive history, excluding assisted reproductive technologies where relevant.		
Birth weight of first child	145,558	10,894,596	European	2017	ukb-a-318	Neale Lab	Birth weight of first child (Neale Lab) is defined as the self-reported birth weight (in grams) of a woman's first-born child, as recorded in the UK Biobank questionnaire data and analysed in the Neale	Continuous	Based on maternal recall; includes women with at least one live birth; implausible values excluded through standard quality control	Maternal self-report; continuous (grams)	Subject to recall bias; availability limited to parous women

							Lab GWAS pipeline. The phenotype reflects maternal report rather than clinically recorded birth weight.				
Number of live births	250,782	9,851,867	European	2018	ukb-b-1209	MRC-IEU	Number of live births (MRC-IEU) is defined as the total self-reported number of live-born children a woman has had. This phenotype is derived primarily from UK Biobank questionnaire data and processed through the MRC-IEU OpenGWAS pipeline.	Count (discrete)	Analysed as a quantitative trait in GWAS; standard quality control applied to exclude implausible values	Self-reported; count variable	Some cohorts model as count vs. quantitativ e trait
Medical abortion	109,910	16,379,616	European	2021	finn-b-O15_AB ORT_MEDICA L	FinnGen	Medical abortion (FinnGen) is defined as a registry-based diagnosis or procedure indicating termination of pregnancy using pharmacological methods, identified from the Finnish national health registers.	Binary (case–control)	Based on hospital discharge and outpatient records; captures non-surgical abortion procedures using diagnosis and/or procedure codes	Binary phenotype	Registry-b ased in FinnGen vs. self-report in some cohorts (if available)

Ever taken oral contraceptive pill	250,440	9,851,867	European	2018	ukb-b-9509	MRC-IEU	Self-reported history of ever using the oral contraceptive pill, derived from UK Biobank questionnaire data and processed through the MRC-IEU OpenGWAS pipeline.	Binary (yes/no)	The phenotype indicates whether a participant has ever used oral contraceptives at any point in their lifetime	Binary (yes/no)	No information on duration, formulation, or timing
Ever used hormone-replacement therapy	250,301	9,851,867	European	2018	ukb-b-18541	MRC-IEU	Self-reported history of having ever used hormone-replacement therapy (HRT), derived from UK Biobank questionnaire data and processed through the MRC-IEU OpenGWAS pipeline.	Binary (yes/no)	This phenotype indicates any lifetime use of HRT, regardless of duration or formulation	Binary (yes/no)	No differentiation by HRT type, dose, or duration

**Phenotypes**	**Sample size**	**Number of SNPs**	**Category**	**Consortium (Source)**	**Population**	**Year**	**GWAS ID**	**Cognitive Domain Assessed**	**Rationale for Inclusion**	**Relevance to Cognitive Decline**
**COGNITIVE FUNCTION**										
Cognitive performance	257,841	10,066,414	Continuous	71 quality-controlled cohorts*	European	2018	ebi-a-GCST006572	Global cognition	Provides an overall summary measure of cognitive ability	Declines reflect general cognitive ageing and global impairment
Fluid intelligence score	149,051	9,851,867	Categorical Ordered	MRC-IEU (UK Biobank)	European	2018	ukb-b-5238	Reasoning and problem-solving	Sensitive to age-related cognitive changes	Lower scores associated with early cognitive decline
Memory performance	48,080	9,851,867	Continuous	MRC-IEU (UK Biobank)	European	2018	ukb-b-16872	Short-term and working memory	Memory impairment is a core feature of cognitive decline	Early indicator of mild cognitive impairment

TM: Interval in trail 2 path	105,451	9,851,867	Continuous	MRC-IEU (UK Biobank)	European	2018	ukb-b-16311	Executive function	Assesses task switching and cognitive flexibility	Executive dysfunction is common in early cognitive impairment
TM: Duration to complete trail 2 path	99,477	9,851,867	Continuous	MRC-IEU (UK Biobank)	European	2018	ukb-b-20140	Processing speed and executive control	Sensitive to slowing with ageing	Longer completion times indicate cognitive slowing
SDS: Number of correct matches	113,106	9,851,867	Continuous	MRC-IEU (UK Biobank)	European	2018	ukb-b-15625	Visual memory and attention	Measures accuracy in symbol matching	Reduced accuracy linked to impaired attention and memory
SDS: Number of matches attempted	113,106	9,851,867	Continuous	MRC-IEU (UK Biobank)	European	2018	ukb-b-1806	Processing speed	Reflects speed–accuracy trade-off	Lower attempt rates indicate cognitive slowing
SDS: Duration to entering value	113,410	9,851,867	Continuous	MRC-IEU (UK Biobank)	European	2018	ukb-b-19585	Processing speed	Measures response speed	Increased duration suggests slower cognitive processing
PM: Number of incorrect matches	462,302	9,851,867	Continuous	MRC-IEU (UK Biobank)	European	2018	ukb-b-20498	Attention and error monitoring	Captures executive control and attention	Higher error rates associated with cognitive dysfunction
PM: Time to complete round	454,157	9,851,867	Continuous	MRC-IEU (UK Biobank)	European	2018	ukb-b-13823	Executive function and speed	Reflects efficiency in problem-solving	Longer times indicate reduced cognitive efficiency
Reaction time	2,378	2,415,500	Continuous	Brisbane adolescent twin sample; Lothian Birth Cohort 1936 (LBC1936); Lothian Birth Cohort 1921 (LBC1921); The Helsinki Birth Cohort	European	2011	ieu-a-1066	Processing speed	Widely used marker of cognitive processing speed	Slower reaction times predict cognitive decline

				Study (HBCS)						

Notes. SNP: Single Nucleotide Polymorphism

The 71 quality-controlled cohorts include: 23andMe; Manchester Studies of Cognition (ACPRC); National Longitudinal Study of Adolescent to Adult Health (Add Health); Age, Gene/Environment Susceptibility (AGES); Avon Longitudinal Study of Parents and Children (ALSPAC); Austrian Stroke Prevention Study (ASPS); Berlin Aging Study II (BASE-II); Cohorte Lausannoise (CoLaus); Copenhagen Studies on Asthma in Childhood 2000 (COPSAC2000); Croatia Korčula (CROATIA-Korčula); deCODE genetics (deCODE); Dortmund Health Study (DHS); Wellcome Trust Diabetes and Inflammation Laboratory (DIL); Estonian Genome Center, University of Tartu (EGCUT); English Longitudinal Study of Ageing (ELSA); Erasmus Rucphen Family Study (ERF); Family Heart Study (FamHS); Fenland Study (FENLAND); The National FINRISK Study (FINRISK); Finnish Twin Cohort (FTC); Geisinger Health System (Geisinger); Genetics of Overweight Young Adults (GOYA); Genetic Regulation of Arterial Pressure of Humans In the Community (GRAPHIC); Generation Scotland: Scottish Family Health Study (GSII); Health 2000 Cases and Controls (H2000 Cases, H2000 Controls); Helsinki Birth Cohort Study (HBCS); Hunter Community Study (HCS); Heinz Nixdorf Recall Study – CorexB, Oexpr, Omni1 (HNRS); Health and Retirement Study (HRS); Hypergenes (Hypergenes); Italian Network of Genetic Isolates – CARL and FVG (INGI-CARL, INGI-FVG); Cooperative Health Research in the Region of Augsburg (KORA S3, KORA S4); Lothian Birth Cohorts 1921 and 1936 (LBC1921, LBC1936); The LifeLines Cohort Study (LifeLines); Minnesota Center for Twin and Family Research (MCTFR); Molecular Genetics of Schizophrenia (MGS); Mother and Child Cohort of Norway (MoBa); Nijmegen Biomedical Study (NBS); Netherlands Study of Depression and Anxiety (NESDA); Northern Finland Birth Cohort 1966 (NFBC66); EPIC-Norfolk (NORFOLK); Netherlands Twin Register (NTR); Ogliastra Genetic Park and Talana (OGP, OGP-Talana); Orkney Complex Disease Study (ORCADES); Prevention of Renal and Vascular End-stage Disease (PREVEND); Queensland Institute of Medical Research (QIMR); Rotterdam Studies I–III (RS-I, RS-II, RS-III); Rush Memory and Aging Project and Religious Orders Study (Rush-MAP, Rush-ROS); SardiNIA Study of Aging (SardiNIA); Study of Health in Pomerania and TREND (SHIP, SHIP-TREND); Swedish Twin Registry – Salty and TwinGene (STR – Salty, STR – TwinGene); The Hellenic Study of Interactions between SNPs and Eating in Atherosclerosis Susceptibility (THISEAS); TwinsUK (TwinsUK); UK Biobank (UKB); UK Household Longitudinal Study (UKHLS); Viking Health Study – Shetland (VIKING); Wisconsin Longitudinal Study (WLS); 1958 British Birth Cohort (WTCCC58C); and The Cardiovascular Risk in Young Finns Study (YFS).

Table S2. Genetic variants used as instrumental variables for age at menarche.

SNP	Chromosome	Position	Effect allele	Other allele	Beta	SE	Pval
rs395962	6	105397418	G	T	-0.120	0.0045	1.20E-158
rs12347838	9	108949596	C	T	-0.100	0.0046	3.60E-106
rs7114175	11	122813983	T	A	0.060	0.0042	1.90E-46
rs7852169	9	114318394	G	C	0.100	0.0076	1.21E-39
rs506589	1	177894287	C	T	-0.069	0.0053	3.60E-38
rs9635759	17	49613785	A	G	0.059	0.0046	4.39E-37
rs7576624	2	625029	T	C	-0.071	0.0056	6.83E-37
rs3767357	1	165403451	G	A	-0.080	0.0063	8.11E-37
rs10934420	3	117552111	C	T	0.053	0.0042	1.47E-36
rs6704684	2	56596394	A	G	0.071	0.0057	3.80E-36
rs7359336	16	69733460	A	G	-0.053	0.0042	1.00E-35
rs1512238	18	44748467	G	A	0.053	0.0043	1.20E-35
rs142058842	2	156621725	G	C	0.071	0.0057	2.51E-35
rs10931831	2	199621641	T	C	-0.053	0.0045	1.27E-31
rs10138913	14	60943106	C	T	-0.054	0.0046	1.31E-31
rs1079866	7	41470093	G	C	0.072	0.0062	7.02E-31
rs11075992	16	53820066	C	T	-0.048	0.0043	3.09E-29
rs2724961	8	4560227	C	T	0.048	0.0043	4.23E-29
rs12566985	1	75002193	A	G	0.048	0.0044	6.54E-28
rs62379978	5	133915969	G	T	0.065	0.0061	1.38E-26
rs11873906	18	3813464	A	G	-0.051	0.0048	3.32E-26
rs7256078	19	10006795	T	C	0.046	0.0044	5.95E-26
rs10791503	11	101436561	C	T	0.047	0.0045	6.16E-26
rs7178532	15	23794517	A	G	0.048	0.0046	2.29E-25
rs1659127	16	14388305	A	G	0.048	0.0046	3.67E-25
rs10832021	11	13324530	A	G	-0.048	0.0047	2.40E-24
rs9758500	3	86910329	G	A	0.045	0.0044	3.49E-24
rs2438086	2	105871129	G	A	-0.047	0.0047	1.14E-23
rs2300922	3	185651469	T	C	0.043	0.0043	2.18E-23
rs4363822	15	67992583	G	A	-0.042	0.0043	6.95E-23

rs732898	14	100794126	A	G	0.051	0.0052	1.06E-22
rs6931884	6	100158873	T	C	0.061	0.0063	3.04E-22
rs3743266	15	60781513	C	T	-0.043	0.0045	1.40E-21
rs62104180	2	466003	A	G	0.110	0.0115	1.42E-21
rs1984870	3	24715135	T	G	0.042	0.0044	3.47E-21
rs10746733	9	86712623	G	A	-0.042	0.0044	3.75E-21
rs10938397	4	45182527	G	A	-0.041	0.0044	4.59E-21
rs4804025	19	47609223	A	G	-0.044	0.0047	7.89E-21
rs3733632	4	104640935	G	A	0.054	0.0058	1.10E-20
rs4735762	8	78097322	A	G	-0.042	0.0045	1.27E-20
rs752278	15	89037134	A	G	-0.040	0.0043	2.03E-20
rs138625771	3	51333959	C	G	0.150	0.0162	2.27E-20
rs7132908	12	50263148	A	G	-0.041	0.0044	2.55E-20
rs9522262	13	112186283	G	C	-0.041	0.0044	2.65E-20
rs4897178	6	126727908	G	T	-0.040	0.0044	6.36E-20
rs7896371	10	1729026	T	C	0.040	0.0045	2.88E-19
rs1172955	10	97877320	A	T	-0.042	0.0047	9.22E-19
rs9349203	6	41893323	A	G	-0.037	0.0042	1.61E-18
rs913588	9	7174673	A	G	-0.037	0.0043	5.85E-18
rs3113862	4	95143122	G	A	0.037	0.0043	1.12E-17
rs35330978	16	29880874	T	C	-0.042	0.0049	1.44E-17
rs16937956	11	8404501	G	A	0.037	0.0044	3.23E-17
rs59072247	2	73536689	T	C	0.110	0.0132	8.79E-17
rs9475046	6	54720275	C	G	0.035	0.0042	9.35E-17
rs11210871	1	44029353	G	C	-0.039	0.0048	3.26E-16
rs852061	20	17109159	C	A	0.035	0.0043	4.77E-16
rs1356379	2	209622211	T	C	0.056	0.0069	6.62E-16
rs1380995	1	72733841	T	A	0.034	0.0043	1.91E-15
rs73035994	3	24206463	C	T	0.100	0.0128	5.15E-15
rs4464946	8	87326259	A	G	-0.039	0.0050	5.63E-15
rs4745292	9	76811790	T	C	-0.037	0.0048	8.84E-15
rs10049090	3	156797702	A	G	-0.034	0.0044	9.78E-15

rs1571536	9	92215638	C	T	-0.033	0.0043	1.52E-14
rs4945266	11	78027488	G	A	0.045	0.0059	1.68E-14
rs7678293	4	28742169	G	A	0.037	0.0048	1.69E-14
rs7738950	6	77741872	G	A	-0.040	0.0052	2.09E-14
rs953230	11	46064974	A	G	0.036	0.0048	4.26E-14
rs4561063	1	102520898	G	T	-0.033	0.0044	4.67E-14
rs1994234	17	53229345	G	A	0.033	0.0044	5.76E-14
rs16918378	8	53877882	C	T	-0.050	0.0067	6.30E-14
rs10767659	11	27686196	T	G	0.034	0.0046	9.52E-14
rs1925047	13	74600274	C	A	0.034	0.0046	1.13E-13
rs247520	5	110876057	C	T	-0.038	0.0051	1.27E-13
rs1469039	8	140651779	A	G	0.042	0.0057	1.54E-13
rs2267812	7	74138121	C	A	-0.041	0.0056	1.87E-13
rs1374360	2	200125150	T	C	-0.037	0.0050	2.13E-13
rs62414763	6	76449917	C	G	0.032	0.0044	2.56E-13
rs6901192	6	56862310	G	C	-0.038	0.0052	2.85E-13
rs11668587	19	18829770	G	A	0.034	0.0047	3.32E-13
rs6933660	6	151803754	A	C	-0.033	0.0045	3.93E-13
rs11165924	1	98375448	G	A	-0.034	0.0047	4.27E-13
rs10076858	5	43134752	A	G	-0.034	0.0047	4.51E-13
rs8136272	22	49678782	T	A	-0.035	0.0048	5.23E-13
rs6478680	9	127458468	A	G	0.031	0.0043	5.86E-13
rs7864983	9	83285190	A	G	-0.032	0.0044	5.97E-13
rs12091368	1	199801507	T	C	0.031	0.0043	6.35E-13
rs12549420	8	54024806	T	G	0.038	0.0053	6.79E-13
rs6864818	5	168734867	C	T	-0.037	0.0052	6.80E-13
rs2836950	21	40604429	G	C	-0.032	0.0045	7.10E-13
rs17449243	1	21174429	C	T	0.049	0.0069	1.03E-12
rs1327938	13	59833252	C	T	-0.032	0.0045	1.57E-12
rs9376867	6	100760042	A	G	-0.034	0.0048	1.76E-12
rs10931078	2	184318783	T	C	-0.040	0.0057	1.80E-12
rs35935052	2	142302503	T	G	0.042	0.0060	1.85E-12

rs7259484	19	1813207	G	A	-0.037	0.0053	2.22E-12
rs2688327	8	3768344	A	T	-0.033	0.0047	2.34E-12
rs107068	19	36204690	G	A	0.029	0.0041	2.40E-12
rs9548873	13	40238492	T	C	-0.031	0.0044	2.78E-12
rs148965598	16	19975731	G	A	0.045	0.0064	2.90E-12
rs2999049	3	127878817	C	T	0.033	0.0047	2.97E-12
rs783521	15	83280445	G	A	0.030	0.0043	3.83E-12
rs12603280	17	6034754	A	G	-0.035	0.0051	4.97E-12
rs12168307	22	31294766	G	T	-0.033	0.0048	8.55E-12
rs6027163	20	37297776	A	G	0.035	0.0052	1.08E-11
rs34635213	2	61612865	G	A	0.042	0.0062	1.21E-11
rs7144872	14	93920422	T	C	-0.031	0.0046	1.25E-11
rs62229372	21	37692507	T	C	0.046	0.0068	1.27E-11
rs813301	5	52909927	T	C	0.029	0.0043	1.34E-11
rs94194	22	22288309	C	T	-0.029	0.0043	1.48E-11
rs2783994	9	1676170	A	G	-0.030	0.0045	1.99E-11
rs77530428	12	17126283	G	A	0.120	0.0181	3.04E-11
rs66752974	3	137057630	A	C	0.038	0.0057	3.26E-11
rs2798224	4	3267668	A	G	0.028	0.0042	4.04E-11
rs6597884	10	126851174	C	T	0.028	0.0043	4.57E-11
rs7719067	5	153538241	G	A	0.028	0.0043	4.78E-11
rs2046549	9	11808441	C	G	-0.034	0.0052	5.56E-11
rs2770957	5	180656734	G	C	-0.034	0.0052	6.20E-11
rs2042067	7	132651302	C	T	0.028	0.0043	6.56E-11
rs12571664	10	121708929	C	T	-0.035	0.0054	6.61E-11
rs62316795	4	132621869	A	C	0.038	0.0058	6.77E-11
rs10237306	7	121955981	T	G	0.029	0.0044	6.85E-11
rs12663002	6	28441634	T	C	0.042	0.0064	7.02E-11
rs2625387	11	43613426	A	G	0.029	0.0045	8.27E-11
rs3782120	11	206089	A	G	0.032	0.0049	9.13E-11
rs59086897	2	25145173	A	T	-0.029	0.0045	9.70E-11
rs9310564	3	18438158	G	A	-0.029	0.0045	9.73E-11

rs11140404	9	86768740	T	C	-0.047	0.0073	1.27E-10
rs2240330	5	137780631	T	C	-0.036	0.0056	1.40E-10
rs240788	6	100984106	G	T	-0.028	0.0044	1.49E-10
rs10400136	10	120833948	A	G	-0.028	0.0044	1.76E-10
rs11070591	15	47816374	A	G	0.030	0.0047	1.90E-10
rs2253310	6	108888593	G	C	-0.028	0.0044	1.98E-10
rs7077302	10	123676662	G	C	-0.050	0.0079	2.10E-10
rs73989733	2	202979964	T	C	-0.043	0.0068	2.15E-10
rs11575886	20	54823550	G	A	-0.043	0.0068	2.26E-10
rs7108556	11	86716236	T	C	0.033	0.0052	3.10E-10
rs7644997	3	114594882	G	A	-0.046	0.0073	3.87E-10
rs29938	19	34311481	C	T	-0.028	0.0045	4.03E-10
rs59246405	17	43123625	T	C	0.027	0.0043	4.87E-10
rs7273470	20	33456921	C	G	0.031	0.0050	5.23E-10
rs4402316	11	84780098	C	G	0.032	0.0052	6.05E-10
rs60024815	17	78779664	T	A	-0.031	0.0050	7.06E-10
rs7128359	11	16787922	T	G	-0.036	0.0058	7.09E-10
rs11031040	11	30317733	G	T	0.035	0.0057	9.00E-10
rs443252	20	62799680	C	T	-0.062	0.0102	1.06E-09
rs437836	5	156715068	C	T	-0.035	0.0057	1.08E-09
rs794361	7	75191602	A	T	0.027	0.0044	1.14E-09
rs9426832	1	154631886	G	A	-0.026	0.0043	1.23E-09
rs9497905	6	148275528	A	C	0.031	0.0051	1.28E-09
rs78928932	6	136228617	C	T	0.061	0.0101	1.38E-09
rs7478970	11	29118542	A	G	-0.032	0.0053	1.42E-09
rs9647570	5	167370263	G	T	0.037	0.0061	1.46E-09
rs4739183	8	78854425	T	C	-0.027	0.0045	1.69E-09
rs1126930	12	49399132	C	G	-0.077	0.0128	1.90E-09
rs758747	16	3627358	T	C	-0.029	0.0048	1.90E-09
rs302719	1	8490320	G	T	0.028	0.0047	1.91E-09
rs6185	8	25280800	G	C	0.029	0.0049	2.37E-09
rs10750766	11	65473798	A	C	-0.029	0.0049	2.45E-09

rs13278754	8	34902952	C	G	-0.028	0.0047	2.52E-09
rs2312205	2	69704941	G	A	-0.033	0.0055	2.70E-09
rs74626840	9	120316487	G	A	0.053	0.0089	2.92E-09
rs13233916	7	138874416	G	C	0.049	0.0083	2.96E-09
rs13218577	6	128328289	C	T	-0.029	0.0049	3.03E-09
rs970953	2	164514239	C	T	0.029	0.0049	3.04E-09
rs11215424	11	115087421	G	A	0.029	0.0049	3.06E-09
rs117008125	15	64625413	A	G	-0.075	0.0127	3.46E-09
rs6968642	7	93312679	T	C	-0.026	0.0044	3.49E-09
rs7628689	3	88216647	G	A	-0.035	0.0059	4.03E-09
rs17035311	4	106066293	C	A	-0.035	0.0060	5.17E-09
rs3197999	3	49721532	A	G	-0.027	0.0046	5.65E-09
rs62120396	19	18346003	T	C	-0.028	0.0048	5.85E-09
rs13219335	6	76928081	T	C	0.026	0.0045	6.16E-09
rs7826872	8	132071766	T	C	0.026	0.0045	6.28E-09
rs610445	19	7891514	G	C	0.030	0.0052	6.38E-09
rs9271761	6	32594022	A	C	0.032	0.0055	6.54E-09
rs10134333	14	30501371	T	C	-0.064	0.0110	6.58E-09
rs111558392	20	20348962	T	C	-0.036	0.0062	7.12E-09
rs4951261	1	205717823	C	A	-0.025	0.0043	7.24E-09
rs845193	1	7421139	G	A	-0.029	0.0050	7.28E-09
rs77751245	8	105326459	G	T	-0.026	0.0045	7.68E-09
rs17328643	2	153529785	G	T	0.041	0.0071	7.72E-09
rs11191541	10	104824199	G	A	0.025	0.0043	8.31E-09
rs7924036	10	65191645	T	G	0.025	0.0043	8.62E-09
rs28532925	15	24540383	G	A	-0.043	0.0075	8.68E-09
rs10885077	10	112759731	G	T	-0.028	0.0049	9.03E-09
rs2343507	1	162895515	A	C	0.025	0.0044	9.56E-09
rs61846901	10	51056858	T	C	-0.027	0.0047	9.73E-09
rs141164370	1	151168409	T	C	0.082	0.0143	9.91E-09
rs113557523	11	94085099	T	C	-0.044	0.0077	1.05E-08
rs34415424	8	4845681	C	T	-0.028	0.0049	1.07E-08

rs147622113	19	10771941	T	C	0.085	0.0149	1.10E-08
rs2244634	1	14134154	T	G	-0.031	0.0054	1.12E-08
rs7979001	12	97506357	A	G	0.024	0.0042	1.47E-08
rs72768041	1	241795639	A	T	-0.074	0.0131	1.53E-08
rs17351032	3	132560225	C	T	0.033	0.0058	1.56E-08
rs34938743	19	5044043	G	A	0.028	0.0050	1.65E-08
rs12156559	9	96446863	T	C	-0.029	0.0051	1.76E-08
rs2425674	20	43529461	G	C	0.024	0.0043	1.94E-08
rs10011174	4	153495515	A	G	0.026	0.0047	2.29E-08
rs9568123	13	49475780	G	A	0.033	0.0059	2.42E-08
rs13120031	4	177465182	T	C	0.025	0.0045	2.49E-08
rs4886869	15	77799657	G	A	-0.024	0.0043	2.51E-08
rs2236129	1	41617914	G	C	-0.044	0.0079	2.55E-08
rs4741022	9	10249081	T	A	0.026	0.0047	2.72E-08
rs4746113	10	74071178	A	G	-0.026	0.0047	2.75E-08
rs928593	10	2718972	C	T	0.039	0.0070	2.86E-08
rs2106405	12	111394644	A	G	-0.024	0.0043	2.90E-08
rs77955256	3	44883523	A	T	-0.039	0.0070	2.92E-08
rs3764002	12	108618630	T	C	-0.028	0.0050	2.93E-08
rs582780	3	172121443	G	A	-0.025	0.0045	3.08E-08
rs15671	7	94186064	C	A	0.024	0.0043	3.34E-08
rs913199	1	65871062	T	G	-0.024	0.0043	3.35E-08
rs67679562	10	90247827	A	G	0.033	0.0060	3.46E-08
rs55680968	17	7774047	G	A	0.046	0.0084	3.74E-08
rs7562809	2	65278144	C	G	0.041	0.0075	3.84E-08
rs2548459	19	49209339	C	T	0.024	0.0044	4.09E-08
rs213558	14	78634174	A	G	0.028	0.0051	4.10E-08
rs11071023	15	54362745	A	G	-0.024	0.0044	4.18E-08
rs10868968	9	73794592	T	G	-0.023	0.0042	4.32E-08
rs77848509	14	101114062	T	G	0.045	0.0082	4.38E-08
rs7605368	2	42984340	A	G	-0.028	0.0051	4.52E-08
rs8853	12	115108907	C	T	0.024	0.0044	4.54E-08

rs7072571	10	71380093	G	A	-0.033	0.0060	4.63E-08
rs9330454	9	92515514	A	G	-0.026	0.0048	4.81E-08
rs11062204	12	2475403	C	T	-0.024	0.0044	4.91E-08
rs11889802	2	60456933	T	G	-0.039	0.0072	4.96E-08
rs17268	7	142510350	A	G	0.027	0.0050	4.96E-08
rs11673135	19	31044844	C	G	-0.032	0.0059	4.97E-08

Notes. SNP: Single Nucleotide Polymorphism; SE: Standard Error

Table S3. Genetic variants used as instrumental variables for age at menopause.

SNP	Chromosome	Position	Effect allele	Other allele	Beta	SE	Pval
rs16991615	20	5948227	A	G	0.243	0.0077	1.00E-200
rs34962991	19	55827175	A	G	-0.098	0.0038	2.90E-145
rs75770066	12	66704225	G	A	0.231	0.0106	8.09E-105
rs28797500	8	37884310	C	T	-0.085	0.0044	3.80E-81
rs58279426	5	176448139	C	T	0.068	0.0037	1.50E-74
rs1565909	4	84400330	T	C	0.062	0.0037	3.50E-64
rs77100210	12	10875928	C	A	0.113	0.0084	5.60E-41
rs9358956	6	10887253	C	G	-0.066	0.0049	1.60E-40
rs251723	16	11953080	C	G	0.052	0.0039	2.00E-40
rs2277339	12	57146069	G	T	-0.078	0.0060	9.70E-39
rs12503643	4	185746088	T	G	0.047	0.0038	2.60E-35
rs2844466	6	31601012	C	T	-0.047	0.0038	8.30E-35
rs9438982	1	39358143	A	C	-0.047	0.0039	7.40E-33
rs9788714	15	89781855	A	G	-0.043	0.0038	1.80E-29
rs9915489	17	41173226	T	A	0.044	0.0039	7.20E-29
rs809673	2	27656036	G	A	-0.042	0.0038	1.70E-28
rs7347500	20	61287669	A	G	-0.049	0.0044	4.50E-28
rs4821798	22	39021165	C	T	-0.043	0.0040	1.20E-27
rs62156695	2	67649768	G	A	-0.064	0.0060	2.70E-26
rs11031005	11	30226356	C	T	0.055	0.0053	3.90E-25
rs17650301	17	62479273	C	A	-0.040	0.0040	2.80E-23
rs112217463	15	41438563	A	G	-0.037	0.0038	1.50E-22
rs4886238	13	61113739	A	G	0.038	0.0039	6.30E-22
rs28416520	12	130823657	A	G	-0.036	0.0037	1.20E-21
rs6830848	4	48690408	T	G	-0.035	0.0037	3.60E-21
rs2941506	17	37833035	G	A	0.037	0.0040	5.20E-21
rs112869704	1	46780285	T	C	0.037	0.0040	7.40E-21
rs349306	19	950694	A	G	0.053	0.0056	8.60E-21
rs76928871	2	48005821	G	A	0.043	0.0047	4.30E-20
rs4668354	2	171814750	G	C	0.035	0.0038	5.60E-20

rs7087644	10	97826334	G	A	-0.084	0.0093	2.00E-19
rs419128	5	6739791	A	G	-0.035	0.0039	2.20E-19
rs1760940	14	20938251	C	A	-0.038	0.0043	4.40E-19
rs6500437	16	89789898	C	T	-0.035	0.0040	4.60E-19
rs11650324	17	5330186	G	A	0.039	0.0044	1.10E-18
rs10854167	20	61533039	C	G	-0.039	0.0045	1.90E-18
rs4495657	1	180949131	C	T	0.033	0.0038	2.40E-18
rs728900	10	131590300	A	T	-0.032	0.0038	3.20E-17
rs60907808	19	23104068	G	A	-0.044	0.0053	3.20E-17
rs10937153	3	183562925	A	G	0.036	0.0043	7.40E-17
rs9313736	5	175948316	A	G	-0.031	0.0038	8.00E-17
rs112190116	7	144096158	T	C	0.143	0.0176	5.10E-16
rs4408133	1	242049649	C	G	0.032	0.0039	6.90E-16
rs299168	19	56317058	A	G	0.051	0.0063	8.00E-16
rs72814771	5	173471004	G	T	0.049	0.0063	2.90E-15
rs10998203	10	70224532	G	C	-0.036	0.0046	3.30E-15
rs4782369	16	88531861	C	G	0.030	0.0039	2.10E-14
rs7132277	12	123593382	T	C	-0.036	0.0047	2.20E-14
rs7125555	11	32549463	T	C	-0.028	0.0037	6.50E-14
rs4491723	2	152280246	G	A	0.031	0.0042	1.10E-13
rs7499238	16	33909759	T	C	-0.029	0.0040	2.10E-13
rs72827480	2	121146501	C	T	0.027	0.0038	6.00E-13
rs7778113	7	5448087	T	G	0.028	0.0039	9.40E-13
rs9307242	4	100934530	C	T	-0.027	0.0038	1.20E-12
rs77055915	12	66735567	C	G	-0.142	0.0201	1.50E-12
rs7589040	2	135682208	T	C	-0.032	0.0045	2.40E-12
rs2519673	7	105994726	A	G	-0.027	0.0038	2.40E-12
rs10521305	16	53908484	C	T	0.054	0.0078	2.70E-12
rs74742883	7	99785765	G	T	-0.031	0.0045	3.00E-12
rs2304192	19	3933314	G	A	-0.028	0.0040	3.00E-12
rs6667957	1	244613791	C	T	-0.026	0.0037	4.30E-12
rs9818740	3	135939586	A	G	-0.028	0.0041	1.70E-11

rs6139074	20	63244	C	A	-0.031	0.0046	1.80E-11
rs8106745	19	46892168	C	T	0.031	0.0046	2.00E-11
rs2688194	5	154257868	C	T	0.044	0.0067	3.60E-11
rs10813912	9	32975210	G	A	0.025	0.0038	6.00E-11
rs746748	20	25282967	T	C	0.047	0.0073	7.50E-11
rs1815198	17	55360585	G	A	-0.025	0.0038	9.60E-11
rs2524119	6	31229404	C	T	0.024	0.0037	1.20E-10
rs6961014	7	128692710	G	C	-0.029	0.0045	1.20E-10
rs12132692	1	169732743	T	C	0.040	0.0063	1.30E-10
rs4049337	7	56162172	C	G	-0.026	0.0040	1.50E-10
rs72708144	1	149815740	C	T	0.059	0.0092	1.70E-10
rs345985	3	156397174	T	C	-0.024	0.0037	1.90E-10
rs2624847	3	50174197	T	G	0.027	0.0042	2.10E-10
rs6470643	8	129621777	C	A	-0.028	0.0045	2.40E-10
rs17856037	14	34985658	T	C	-0.074	0.0117	2.90E-10
rs4679121	3	126160601	T	C	-0.038	0.0060	3.10E-10
rs536092	20	48501351	T	C	0.024	0.0038	3.90E-10
rs6912979	6	160120799	C	T	-0.026	0.0041	4.30E-10
rs6435156	2	203425475	T	C	-0.026	0.0042	4.50E-10
rs1247483	10	78008749	T	C	-0.025	0.0040	7.60E-10
rs8045589	16	9210373	T	A	-0.023	0.0037	9.70E-10
rs11180609	12	76040392	C	T	-0.046	0.0076	9.80E-10
rs77952879	8	103636045	C	G	-0.042	0.0069	1.30E-09
rs7827991	8	81754701	A	C	0.042	0.0069	1.30E-09
rs507926	3	9008174	C	T	0.030	0.0049	1.50E-09
rs28651018	12	122186268	T	C	0.022	0.0037	1.50E-09
rs61870304	10	126680627	G	A	-0.037	0.0061	1.70E-09
rs888368	15	86302283	G	A	0.022	0.0037	1.70E-09
rs74701710	10	13208912	A	G	-0.048	0.0080	1.80E-09
rs62445870	7	50514904	T	C	0.075	0.0124	1.90E-09
rs9613667	22	29127412	C	A	-0.023	0.0039	2.00E-09
rs17820747	2	32701687	C	A	0.026	0.0043	2.10E-09

rs57652769	16	79753976	T	C	0.024	0.0040	2.80E-09
rs7661090	4	13571901	T	C	-0.035	0.0060	3.20E-09
rs11571818	13	32968810	C	T	-0.113	0.0191	3.30E-09
rs7845046	8	48926264	A	T	-0.049	0.0084	4.40E-09
rs1020622	11	9469792	G	C	0.022	0.0037	4.70E-09
rs783562	9	23823399	A	G	0.022	0.0038	4.90E-09
rs17646517	3	44286015	G	C	-0.072	0.0123	5.10E-09
rs12046563	1	43137280	G	A	-0.025	0.0043	5.50E-09
rs200448	1	6701978	C	T	-0.021	0.0037	8.10E-09
rs55848327	15	63819427	A	G	0.025	0.0044	8.80E-09
rs10476835	5	141675985	A	G	0.021	0.0037	1.00E-08
rs1655907	6	29918587	C	T	-0.029	0.0051	1.10E-08
rs17680522	16	9069331	G	A	0.023	0.0041	1.20E-08
rs78080415	6	111537016	C	T	0.030	0.0052	1.30E-08
rs9607474	22	22336708	T	C	-0.032	0.0057	1.50E-08
rs4716056	6	16278390	G	A	0.021	0.0038	1.60E-08
rs7779	1	246930564	C	G	0.040	0.0071	1.70E-08
rs73037453	19	33464225	T	C	-0.026	0.0047	1.70E-08
rs1868673	3	150187314	C	A	-0.021	0.0038	2.50E-08
rs6584351	10	101970997	G	A	0.020	0.0037	2.60E-08
rs10937595	3	193384203	A	T	0.021	0.0037	2.70E-08
rs2241522	15	42127734	G	A	-0.022	0.0039	2.90E-08
rs394448	19	56452144	C	G	-0.020	0.0037	3.20E-08
rs34811474	4	25408838	A	G	-0.024	0.0044	4.50E-08
rs6736096	2	216958369	C	T	0.020	0.0037	4.70E-08
rs156520	4	155690081	A	C	0.022	0.0041	4.90E-08

Notes. SNP: Single Nucleotide Polymorphism; SE: Standard Error

Table S4. Genetic variants used as instrumental variables for age at first sexual intercourse.

SNP	Chromosome	Position	Effect allele	Other allele	Beta	SE	Pval
rs12204714	6	152235339	T	C	0.029	0.0029	6.30E-24
rs961522	2	58272502	C	T	0.028	0.0028	8.60E-23
rs13005495	2	60157097	G	T	-0.025	0.0028	5.50E-19
rs73077107	3	49671037	A	G	0.033	0.0042	2.80E-15
rs226488	5	87896330	C	T	-0.022	0.0029	3.70E-15
rs12714592	3	84387950	C	A	-0.024	0.0031	7.50E-15
rs12896157	14	41059664	A	G	-0.027	0.0035	3.30E-14
rs3896224	10	106467853	G	A	0.021	0.0028	7.00E-14
rs12089815	1	91189933	A	G	0.021	0.0028	7.20E-14
rs7558132	2	173934771	A	G	-0.021	0.0029	3.70E-13
rs359240	2	60475008	A	G	-0.021	0.0028	5.20E-13
rs784255	18	53403228	T	G	-0.020	0.0028	7.60E-13
rs16948048	17	47440466	G	A	-0.020	0.0029	1.30E-12
rs66632973	3	85585552	T	A	0.024	0.0034	1.60E-12
rs79108591	14	98599967	A	G	-0.023	0.0034	1.40E-11
rs6767258	3	88182893	A	G	-0.025	0.0038	6.60E-11
rs1226414	2	157109930	T	A	0.018	0.0028	7.10E-11
rs6962772	7	99081730	G	A	0.025	0.0038	8.40E-11
rs12528918	6	67554041	G	T	-0.018	0.0028	1.30E-10
rs79532211	4	112396533	G	A	0.025	0.0040	1.80E-10
rs1580173	3	107955515	A	G	-0.018	0.0028	1.90E-10
rs140504125	18	77567765	T	C	-0.020	0.0031	1.90E-10
rs2910032	5	152540354	T	C	0.017	0.0028	2.40E-10
rs62136829	2	44136481	T	C	0.040	0.0064	2.60E-10
rs4552798	7	1928001	T	C	0.018	0.0028	3.10E-10
rs72712556	4	140900251	A	G	0.018	0.0030	3.20E-10
rs11037662	11	43851308	T	G	0.025	0.0040	3.90E-10
rs10510025	10	118650996	T	C	-0.021	0.0032	4.30E-10
rs28399241	8	8663215	T	C	-0.017	0.0028	7.80E-10
rs1372173	8	87684674	G	A	0.022	0.0037	7.90E-10

rs62435144	7	1279105	A	G	0.030	0.0050	1.20E-09
rs57945129	3	117641724	T	C	0.024	0.0039	1.40E-09
rs4727799	7	114110568	T	C	-0.018	0.0029	1.50E-09
rs7942078	11	28656064	T	A	0.018	0.0029	1.50E-09
rs9397485	6	152434828	G	A	0.019	0.0031	1.60E-09
rs13097782	3	35739575	T	C	0.017	0.0028	2.10E-09
rs11627661	14	79556618	C	G	-0.017	0.0029	2.70E-09
rs198310	7	24191861	T	A	0.020	0.0034	3.50E-09
rs2152741	13	67135121	T	C	0.023	0.0039	3.70E-09
rs62519833	8	65487989	G	A	0.026	0.0044	4.30E-09
rs8033799	15	47681384	C	A	-0.020	0.0034	4.40E-09
rs7079070	10	134182921	A	G	-0.016	0.0028	4.50E-09
rs12203592	6	396321	T	C	0.021	0.0034	4.60E-09
rs7815972	8	118975279	G	A	0.017	0.0029	4.60E-09
rs11240331	1	204968339	T	C	0.019	0.0032	5.10E-09
rs10260121	7	115110126	C	T	-0.025	0.0043	5.50E-09
rs57383707	18	1811604	T	C	-0.025	0.0044	7.50E-09
rs76513770	16	72505534	C	T	0.023	0.0041	8.50E-09
rs11214488	11	112971545	T	C	-0.020	0.0036	8.60E-09
rs7635829	3	147094222	A	G	-0.019	0.0032	9.50E-09
rs1157072	1	72634912	G	A	0.036	0.0063	9.60E-09
rs62166484	2	142340046	A	T	-0.031	0.0055	1.10E-08
rs113367286	7	140144414	T	C	0.017	0.0031	1.20E-08
rs4800092	18	36920318	T	C	-0.016	0.0028	1.20E-08
rs11656471	17	32899382	T	G	-0.020	0.0036	1.30E-08
rs62177787	2	63456210	G	C	0.019	0.0034	1.50E-08
rs6549670	3	74936132	G	A	-0.022	0.0038	1.60E-08
rs62499803	8	53175090	G	A	-0.020	0.0036	1.70E-08
rs72693550	9	23735995	A	C	-0.022	0.0038	1.70E-08
rs186723454	3	54156598	G	A	-0.022	0.0039	1.80E-08
rs11196020	10	114375603	C	T	0.025	0.0045	2.00E-08
rs72931396	2	200301157	A	C	0.016	0.0028	2.30E-08

rs57537843	2	22558973	A	G	-0.016	0.0029	2.30E-08
rs11076963	16	5816651	G	C	-0.019	0.0034	2.60E-08
rs79155408	13	56969707	T	C	-0.034	0.0061	2.80E-08
rs10219714	12	19119135	T	C	0.016	0.0029	3.00E-08
rs2684837	18	44749884	C	A	0.015	0.0028	3.00E-08
rs11678980	2	162101261	A	G	-0.016	0.0029	3.30E-08
rs10761784	10	65308750	T	A	0.015	0.0028	3.30E-08
rs12759968	1	66341400	T	C	0.020	0.0036	3.40E-08
rs4768354	12	41824245	T	C	0.015	0.0028	3.70E-08
rs11131357	4	62966257	T	C	0.016	0.0030	4.10E-08
rs12541633	8	36706700	T	A	0.016	0.0028	4.30E-08
rs17425189	1	57862949	C	T	0.020	0.0036	4.40E-08
rs3007104	14	47367434	A	G	-0.015	0.0028	4.50E-08
rs66906321	2	630070	C	T	-0.021	0.0037	4.60E-08
rs7349386	2	213773970	C	T	0.036	0.0065	4.70E-08
rs770082	12	89776485	A	G	-0.015	0.0028	4.80E-08

Notes. SNP: Single Nucleotide Polymorphism; SE: Standard Error

Table S5. Genetic variants used as instrumental variables for age at first birth.

SNP	Chromosome	Position	Effect allele	Other allele	Beta	SE	Pval
rs12407439	1	22347396	G	A	0.088	0.0151	6.86E-09
rs7517629	1	91196099	G	A	0.066	0.0111	3.02E-09
rs2069278	1	66635371	C	T	-0.059	0.0105	1.87E-08
rs3767654	1	220331319	G	T	0.072	0.0125	6.16E-09
rs72694234	1	153823918	G	T	-0.078	0.0108	7.01E-13
rs2906457	1	44338575	C	A	0.068	0.0112	1.43E-09
rs12739999	1	32207990	A	G	-0.076	0.0138	3.89E-08
rs7516843	1	210322929	G	A	0.062	0.0105	3.37E-09
rs359240	2	60475008	A	G	-0.079	0.0103	1.58E-14
rs11887646	2	5891511	G	A	0.077	0.0121	2.14E-10
rs80153284	2	156465747	A	C	0.246	0.0439	2.17E-08
rs12613825	2	51936388	C	G	-0.082	0.0148	2.59E-08
rs73989464	2	215241551	G	A	0.075	0.0132	1.20E-08
rs1106090	2	58068741	A	G	0.061	0.0102	2.82E-09
rs72779695	2	12797853	T	C	-0.087	0.0158	3.76E-08
rs7562372	2	100906331	C	T	0.063	0.0103	9.55E-10
rs13425787	2	104135952	G	A	-0.071	0.0102	2.80E-12
rs112282597	2	166250414	G	A	0.070	0.0122	8.37E-09
rs2681780	3	49897830	T	C	-0.120	0.0101	7.52E-33
rs1376961	3	83510683	T	C	0.072	0.0125	9.46E-09
rs9814726	3	17953353	A	T	0.062	0.0102	1.84E-09
rs13319205	3	47800216	A	T	-0.067	0.0115	7.45E-09
rs11915934	3	74883069	G	A	-0.083	0.0133	4.75E-10
rs17314804	4	140946828	T	C	0.074	0.0103	7.79E-13
rs362307	4	3241845	T	C	-0.126	0.0194	7.93E-11
rs329122	5	133864599	A	G	0.057	0.0102	1.90E-08
rs12153189	5	45109961	G	C	0.088	0.0134	4.51E-11
rs1445979	5	60744339	A	T	-0.056	0.0101	2.65E-08
rs2347867	6	152229850	A	G	0.086	0.0103	6.74E-17
rs3096695	6	32069806	C	G	-0.082	0.0141	4.83E-09

rs9388090	6	98527087	T	C	0.067	0.0107	3.81E-10
rs11155364	6	144952491	A	C	-0.087	0.0156	2.36E-08
rs6964957	7	151330390	T	C	0.058	0.0102	9.87E-09
rs55988458	7	2059761	A	G	0.079	0.0129	9.46E-10
rs2009182	7	3445547	C	T	0.070	0.0118	2.76E-09
rs1859100	7	114194615	G	T	-0.061	0.0103	2.09E-09
rs11774212	8	145686505	T	C	0.066	0.0105	3.04E-10
rs10111950	8	10815754	G	C	0.062	0.0105	3.26E-09
rs1590949	9	23360417	G	C	0.069	0.0103	2.14E-11
rs7865801	9	14745886	A	G	0.065	0.0105	6.38E-10
rs12250380	10	106587950	G	A	0.063	0.0100	2.56E-10
rs6585429	10	118893231	G	A	0.096	0.0134	9.03E-13
rs11031006	11	30226528	A	G	0.088	0.0141	3.77E-10
rs7481939	11	28668284	G	A	0.058	0.0105	4.74E-08
rs1702877	12	56427808	T	C	0.063	0.0107	3.90E-09
rs12815479	12	123430082	T	C	0.108	0.0188	9.89E-09
rs7958796	12	84043145	T	A	-0.080	0.0110	3.81E-13
rs78702863	13	67143823	T	C	-0.105	0.0178	4.37E-09
rs176223	14	29604585	C	T	0.090	0.0149	1.82E-09
rs6574018	14	72213258	T	G	0.060	0.0109	3.24E-08
rs72704712	14	103312965	G	T	-0.100	0.0146	6.62E-12
rs1950402	14	27085685	G	A	-0.068	0.0115	3.66E-09
rs1452378	15	74100132	C	T	0.075	0.0135	2.56E-08
rs8027457	15	99204101	C	T	-0.054	0.0098	4.53E-08
rs11646744	16	59241949	T	G	-0.075	0.0121	7.13E-10
rs77576048	16	90078724	T	C	0.100	0.0180	2.94E-08
rs10445366	17	43932797	C	G	-0.089	0.0149	2.61E-09
rs7359501	17	56641200	T	C	0.063	0.0102	1.01E-09
rs4799950	18	35170045	G	C	0.063	0.0109	7.82E-09
rs60222682	18	36510587	T	C	-0.072	0.0127	1.21E-08
rs590076	18	53260732	A	G	-0.067	0.0103	1.06E-10
rs8110682	19	13285293	C	T	0.087	0.0119	2.59E-13

rs6079584	20	14840502	C	A	0.055	0.0100	3.55E-08
rs293566	20	31097877	C	T	-0.071	0.0108	4.42E-11
rs6030812	20	42001011	A	G	0.070	0.0128	4.93E-08
rs394608	21	46581798	C	T	-0.057	0.0102	1.82E-08

Notes. SNP: Single Nucleotide Polymorphism; SE: Standard Error

Table S6. Genetic variants used as instrumental variables for birth weight of first child .

SNP	Chromosome	Position	Effect allele	Other allele	Beta	SE	Pval
rs2964484	5	157897437	A	G	0.058	0.0050	5.51E-31
rs9856468	3	155856843	T	C	0.044	0.0045	3.46E-22
rs597808	12	111973358	G	A	0.042	0.0045	5.79E-21
rs10830963	11	92708710	G	C	0.046	0.0050	1.97E-20
rs2918301	19	8786918	T	C	-0.052	0.0062	2.00E-17
rs1015521	4	106115450	T	G	0.039	0.0047	2.19E-16
rs72760655	9	116916214	A	C	-0.039	0.0048	3.28E-16
rs12205495	6	35484850	A	T	0.042	0.0054	4.24E-15
rs1374204	2	46484205	T	C	0.039	0.0050	1.71E-14
rs34573359	10	96077576	T	C	-0.046	0.0061	3.52E-14
rs7970350	12	66360164	T	C	-0.033	0.0045	8.87E-14
rs45446698	7	99332948	G	T	0.080	0.0110	2.67E-13
rs17666239	12	47194757	A	T	0.065	0.0089	3.35E-13
rs7177338	15	91428636	A	G	0.032	0.0045	6.22E-13
rs12909648	15	86224570	A	G	-0.032	0.0045	1.32E-12
rs150459394	6	34185960	T	A	0.062	0.0088	1.72E-12
rs4677883	3	123052018	C	A	0.040	0.0057	1.86E-12
rs138715366	7	44246271	T	C	-0.167	0.0239	2.91E-12
rs62066209	17	17520784	C	T	0.032	0.0046	4.83E-12
rs17097560	11	102099495	C	G	0.032	0.0047	7.07E-12
rs2647873	12	103081192	G	A	-0.030	0.0044	7.45E-12
rs17367504	1	11862778	G	A	0.040	0.0060	2.38E-11
rs2131354	4	145599908	A	G	0.030	0.0045	2.51E-11
rs9379084	6	7231843	A	G	-0.048	0.0072	3.18E-11
rs2168101	11	8255408	A	C	-0.033	0.0050	5.20E-11
rs4559047	5	133848660	T	C	0.029	0.0045	8.29E-11
rs10762265	10	70986185	C	T	0.030	0.0046	1.06E-10
rs7772305	6	31328886	G	A	-0.032	0.0051	6.07E-10
rs16939343	8	77640595	C	T	0.037	0.0061	9.27E-10
rs34471628	5	172196752	G	A	-0.069	0.0113	9.59E-10

rs116786669	2	62005479	C	T	0.069	0.0113	1.13E-09
rs5030320	11	32410002	C	T	-0.028	0.0049	1.13E-08
rs7854962	9	96900505	G	C	-0.031	0.0055	1.27E-08
rs3918226	7	150690176	T	C	-0.047	0.0083	1.45E-08
rs151240644	12	133045180	A	C	-0.046	0.0081	1.49E-08
rs118117242	14	72971037	T	G	-0.096	0.0170	1.54E-08
rs34417222	20	47450063	T	C	0.027	0.0048	1.86E-08
rs3871468	6	29677641	C	T	0.030	0.0053	2.07E-08
rs10843815	12	30856304	T	C	0.029	0.0051	2.25E-08
rs9855294	3	14269650	T	G	0.029	0.0052	2.42E-08
rs11641308	16	75312023	C	T	-0.026	0.0047	2.45E-08
rs2782538	6	142595051	G	C	-0.025	0.0045	2.66E-08
rs62562578	9	94251654	C	T	0.027	0.0049	3.87E-08
rs4894405	3	139021139	C	T	0.027	0.0050	3.97E-08
rs2061456	4	17998426	A	C	0.028	0.0051	4.22E-08

Notes. SNP: Single Nucleotide Polymorphism; SE: Standard Error

Table S7. Genetic variants used as instrumental variables for number of live births.

SNP	Chromosome	Position	Effect allele	Other allele	Beta	SE	Pval
rs4305732	6	152240448	G	A	-0.026	0.0034	1.70E-14
rs4869737	6	151892135	T	C	-0.026	0.0036	9.90E-13
rs62250537	3	85638325	G	C	-0.023	0.0035	3.60E-11
rs10445367	17	43932798	T	G	0.029	0.0045	1.30E-10
rs12410251	1	22482629	T	G	-0.026	0.0042	5.10E-10
rs28456	11	61589481	G	A	-0.022	0.0036	1.10E-09
rs9862795	3	49915506	T	A	0.020	0.0033	2.00E-09
rs2627197	10	118622128	T	A	-0.022	0.0038	3.60E-09
rs35600310	1	177365960	C	T	-0.023	0.0040	9.50E-09
rs3904477	9	3085018	T	C	-0.028	0.0050	3.10E-08
rs4268748	16	90026512	C	T	-0.020	0.0036	4.00E-08

Notes. SNP: Single Nucleotide Polymorphism; SE: Standard Error

Table S8. Genetic variants used as instrumental variables for medical abortion.

SNP	Chromosome	Position	Effect allele	Other allele	Beta	SE	Pval
rs7078823	10	118885780	C	G	-0.082	0.0148	3.09E-08
rs587692088	1	146400186	T	A	-0.076	0.0138	3.24E-08
rs2240068	6	30076630	T	G	-0.063	0.0122	2.47E-07
rs145033590	19	11135189	A	G	0.317	0.0650	1.03E-06
rs113698798	5	168808905	T	G	-0.074	0.0152	1.36E-06
rs13004241	2	174338216	G	A	0.057	0.0118	1.57E-06
rs77419295	4	140770065	G	A	0.182	0.0379	1.66E-06
rs6035775	20	21060464	A	T	0.082	0.0171	1.81E-06
rs11243639	9	135021758	G	A	-0.056	0.0117	1.94E-06
rs71490987	10	55739294	C	T	-0.118	0.0247	2.03E-06
rs3852716	16	82613327	C	T	0.055	0.0116	2.17E-06
rs182407153	12	24513298	T	C	0.816	0.1731	2.45E-06
rs17573936	18	3962410	T	C	0.055	0.0117	2.84E-06
rs4680338	3	156794425	G	C	0.057	0.0123	3.34E-06
rs17297819	11	29352394	C	T	-0.151	0.0326	3.53E-06
rs73889315	3	192116600	A	G	-0.126	0.0273	3.85E-06
rs11602227	11	95501796	A	G	0.161	0.0348	4.05E-06
rs11079566	17	63439608	T	A	0.059	0.0128	4.06E-06
rs301800	1	8490603	C	T	0.071	0.0154	4.07E-06
rs8054505	16	83615856	A	G	0.059	0.0128	4.33E-06
rs116942581	13	49024551	A	G	-0.222	0.0483	4.48E-06

Notes. SNP: Single Nucleotide Polymorphism; SE: Standard Error

Table S9. Genetic variants used as instrumental variables for oral contraceptive pills.

SNP	Chromosome	Position	Effect allele	Other allele	Beta	SE	Pval
rs12097417	1	196174289	A	G	0.007	0.0014	2.50E-06
rs7605	1	167759281	C	T	0.005	0.0011	3.70E-06
rs73964524	2	144272756	C	A	0.006	0.0014	3.40E-06
rs72835975	2	82385438	T	A	-0.007	0.0015	6.60E-07
rs9837536	3	192583843	A	T	-0.006	0.0011	3.50E-07
rs2362972	3	158163272	A	C	-0.005	0.0011	3.90E-06
rs7427760	3	17988979	G	C	-0.005	0.0011	3.50E-06
rs2410943	5	106453343	G	A	0.005	0.0011	2.50E-06
rs1394616	5	174554046	T	C	-0.007	0.0016	4.90E-06
rs2243779	5	167084904	G	C	0.006	0.0013	1.60E-06
rs140345655	5	49662983	T	C	0.011	0.0023	8.70E-07
rs9270907	6	32572082	T	C	0.006	0.0012	3.30E-06
rs11765362	7	26525621	A	G	-0.006	0.0012	2.30E-06
rs7787856	7	110340600	G	C	-0.005	0.0011	3.40E-06
rs59592870	7	21183026	A	C	-0.013	0.0028	2.60E-06
rs11777472	8	142604448	C	T	-0.007	0.0015	4.10E-06
rs491303	9	1731861	G	T	-0.005	0.0011	9.30E-07
rs7041814	9	16068991	C	G	0.006	0.0012	3.00E-06
rs61910375	11	122415466	G	A	-0.026	0.0055	2.80E-06
rs10845767	12	13573634	C	A	-0.005	0.0012	4.30E-06
rs9537793	13	58331407	A	G	0.005	0.0011	2.30E-06
rs117933126	14	96038148	C	G	-0.025	0.0050	4.80E-07
rs2439355	15	66889053	T	G	-0.007	0.0015	7.10E-07
rs139957871	19	45283883	A	C	-0.014	0.0029	2.60E-06
rs62115110	19	15685472	G	T	0.007	0.0015	4.40E-06
rs695537	22	43769871	G	A	0.006	0.0012	2.30E-06

Notes. SNP: Single Nucleotide Polymorphism; SE: Standard Error

Table S10. Genetic variants used as instrumental variables for ever used hormone-replacement therapy.

SNP	Chromosome	Position	Effect allele	Other allele	Beta	SE	Pval
rs78154848	4	104562840	C	T	-0.033	0.0030	9.60E-29
rs16991615	20	5948227	A	G	-0.025	0.0028	9.40E-20
rs10425848	19	55834448	G	C	0.010	0.0014	1.90E-13
rs28794362	8	37876671	G	A	0.012	0.0017	4.40E-13
rs11586493	1	180961245	A	G	-0.009	0.0014	9.30E-10
rs1716021	11	30225170	C	G	-0.009	0.0014	9.50E-10
rs62338073	4	176733450	T	G	-0.019	0.0034	7.60E-09

Notes. SNP: Single Nucleotide Polymorphism; SE: Standard Error

Table S11. Cochran's Q tests for heterogeneity from MR-IVW and MR-Egger analyses of reproductive traits and cognitive function.

Exposure	Outcome	Method	Q	Q_df	P-value
Age at menarche	Cognitive performance	Inverse variance weighted	707.3537	179	6.82E-64
		MR Egger	697.1531	178	1.57E-62
	Fluid intelligence score	Inverse variance weighted	560.0162	181	2.44E-40
		MR Egger	548.5700	180	6.78E-39
	Memory performance	Inverse variance weighted	237.6035	181	3.02E-03
		MR Egger	237.5665	180	2.60E-03
	TM: Interval in trail 2 path	Inverse variance weighted	360.4756	181	5.26E-14
		MR Egger	360.2505	180	3.91E-14
	TM: Duration to complete trail 2 path	Inverse variance weighted	354.5595	181	2.34E-13
		MR Egger	354.1593	180	1.83E-13
	SDS: Number of correct matches	Inverse variance weighted	322.4515	181	4.97E-10
		MR Egger	321.2166	180	4.88E-10
	SDS: Number of matches attempted	Inverse variance weighted	334.9829	181	2.74E-11
		MR Egger	333.5394	180	2.81E-11
	SDS: Duration to entering value	Inverse variance weighted	311.7783	181	5.29E-09
		MR Egger	311.7158	180	4.04E-09
	PM: Number of incorrect matches	Inverse variance weighted	381.0549	181	2.45E-16
		MR Egger	377.5668	180	4.25E-16
	PM: Time to complete round	Inverse variance weighted	482.0662	181	3.33E-29
		MR Egger	481.9612	180	2.09E-29
	Reaction Time	Inverse variance weighted	102.2634	92	2.18E-01
		MR Egger	96.4836	91	3.27E-01
Age at menopause	Cognitive performance	Inverse variance weighted	367.0130	110	1.96E-29
		MR Egger	367.2383	109	1.15E-29
	Fluid intelligence score	Inverse variance weighted	308.7466	114	7.21E-20
		MR Egger	308.7326	113	4.35E-20
	Memory performance	Inverse variance weighted	136.5331	114	7.39E-02
		MR Egger	136.3266	113	6.68E-02
	TM: Interval in trail 2 path	Inverse variance weighted	156.4920	114	5.11E-03
		MR Egger	153.3143	113	6.96E-03

	TM: Duration to complete trail 2 path	Inverse variance weighted	154.8341	114	6.59E-03
		MR Egger	152.3956	113	7.99E-03
	SDS: Number of correct matches	Inverse variance weighted	208.1690	114	1.75E-07
		MR Egger	203.4491	113	3.88E-07
	SDS: Number of matches attempted	Inverse variance weighted	208.4446	114	1.64E-07
		MR Egger	203.5055	113	3.83E-07
	SDS: Duration to entering value	Inverse variance weighted	215.2977	114	3.16E-08
		MR Egger	211.1332	113	6.27E-08
	PM: Number of incorrect matches	Inverse variance weighted	257.9109	114	3.73E-13
		MR Egger	256.1820	113	4.01E-13
	PM: Time to complete round	Inverse variance weighted	333.1473	114	2.47E-23
		MR Egger	333.0600	113	1.47E-23
	Reaction time	Inverse variance weighted	39.9584	51	8.68E-01
		MR Egger	37.1242	50	9.12E-01
Age at first sexual intercourse	Cognitive performance	Inverse variance weighted	231.2966	58	1.50E-22
		MR Egger	231.2147	57	7.63E-23
	Fluid intelligence score	Inverse variance weighted	189.5740	58	7.06E-16
		MR Egger	188.8238	57	5.00E-16
	Memory Performance	Inverse variance weighted	80.5363	58	2.68E-02
		MR Egger	80.4248	57	2.22E-02
	TM: Interval in trail 2 path	Inverse variance weighted	137.3322	58	2.21E-08
		MR Egger	137.3227	57	1.40E-08
	TM: Duration to complete trail 2 path	Inverse variance weighted	129.6249	58	2.15E-07
		MR Egger	129.5042	57	1.45E-07
	SDS: Number of correct matches	Inverse variance weighted	160.0410	58	1.72E-11
		MR Egger	159.7195	57	1.13E-11
	SDS: Number of matches attempted	Inverse variance weighted	171.2722	58	4.05E-13
		MR Egger	170.9379	57	2.59E-13
	SDS: Duration to entering value	Inverse variance weighted	152.2446	58	2.15E-10
		MR Egger	152.0710	57	1.37E-10
	PM: Number of incorrect matches	Inverse variance weighted	172.9054	58	2.32E-13
		MR Egger	172.9054	57	1.21E-12

	PM: Time to complete round	Inverse variance weighted	216.1735	58	4.45E-20
		MR Egger	216.1506	57	2.28E-20
	Reaction Time	Inverse variance weighted	25.6159	25	4.28E-01
		MR Egger	25.4285	24	3.83E-01
Age at first birth	Cognitive performance	Inverse variance weighted	284.7706	50	1.36E-34
		MR Egger	281.8975	49	1.85E-34
	Fluid intelligence score	Inverse variance weighted	179.1563	50	1.95E-16
		MR Egger	178.2461	49	1.41E-16
	Memory Performance	Inverse variance weighted	82.7877	50	2.43E-03
		MR Egger	82.7526	49	1.83E-03
	TM: Interval in trail 2 path	Inverse variance weighted	86.2870	50	1.09E-03
		MR Egger	83.1182	49	1.68E-03
	TM: Duration to complete trail 2 path	Inverse variance weighted	87.5471	50	8.11E-04
		MR Egger	81.2128	49	2.60E-03
	SDS: Number of correct matches	Inverse variance weighted	68.4498	50	4.25E-02
		MR Egger	64.5166	49	6.77E-02
	SDS: Number of matches attempted	Inverse variance weighted	77.4481	50	7.68E-03
		MR Egger	72.9434	49	1.49E-02
	SDS: Duration to entering value	Inverse variance weighted	75.4676	50	1.15E-02
		MR Egger	70.7806	49	2.25E-02
	PM: Number of incorrect matches	Inverse variance weighted	137.3326	50	4.46E-10
		MR Egger	136.0719	49	3.99E-10
	PM: Time to complete round	Inverse variance weighted	156.6291	50	6.36E-13
		MR Egger	156.4806	49	3.70E-13
	Reaction Time	Inverse variance weighted	32.3874	23	9.24E-02
		MR Egger	31.1851	22	9.24E-02
Birth weight of first child	Cognitive performance	Inverse variance weighted	166.7040	41	4.36E-17
		MR Egger	155.3046	40	1.67E-15
	Fluid intelligence score	Inverse variance weighted	158.0916	43	4.58E-15
		MR Egger	151.6397	42	2.58E-14
	Memory Performance	Inverse variance weighted	60.8946	43	3.73E-02
		MR Egger	60.8593	42	2.99E-02

	TM: Interval in trail 2 path	Inverse variance weighted	89.3876	43	4.25E-05
		MR Egger	89.2814	42	2.93E-05
	TM: Duration to complete trail 2 path	Inverse variance weighted	94.5480	43	9.78E-06
		MR Egger	94.3992	42	6.65E-06
	SDS: Number of correct matches	Inverse variance weighted	71.1105	43	4.47E-03
		MR Egger	70.9485	42	3.45E-03
	SDS: Number of matches attempted	Inverse variance weighted	73.85656	43	2.37E-03
		MR Egger	73.67557	42	1.81E-03
	SDS: Duration to entering value	Inverse variance weighted	65.8726	43	1.40E-02
		MR Egger	65.8281	42	1.08E-02
	PM: Number of incorrect matches	Inverse variance weighted	131.2154	43	7.39E-11
		MR Egger	131.1084	42	4.27E-11
	PM: Time to complete round	Inverse variance weighted	182.6184	43	3.98E-19
		MR Egger	181.8292	42	2.56E-19
	Reaction Time	Inverse variance weighted	14.9213	17	6.01E-01
		MR Egger	14.3089	16	5.76E-01
Medical abortion	Cognitive performance	Inverse variance weighted	41.8300	17	7.10E-04
		MR Egger	41.3693	16	4.90E-04
	Fluid intelligence score	Inverse variance weighted	36.3312	18	6.40E-03
		MR Egger	36.0662	17	4.49E-03
	Memory Performance	Inverse variance weighted	18.5556	18	4.20E-01
		MR Egger	18.2149	17	3.75E-01
	TM: Interval in trail 2 path	Inverse variance weighted	27.9079	18	6.35E-02
		MR Egger	27.6045	17	4.98E-02
	TM: Duration to complete trail 2 path	Inverse variance weighted	29.0851	18	4.73E-02
		MR Egger	28.6923	17	3.75E-02
	SDS: Number of correct matches	Inverse variance weighted	23.2652	18	1.81E-01
		MR Egger	22.4205	17	1.69E-01
	SDS: Number of matches attempted	Inverse variance weighted	24.2344	18	1.47E-01
		MR Egger	23.0597	17	1.47E-01
	SDS: Duration to entering value	Inverse variance weighted	19.8586	18	3.41E-01
		MR Egger	19.8435	17	2.82E-01

	PM: Number of incorrect matches	Inverse variance weighted	22.2686	18	2.20E-01
		MR Egger	15.8508	17	5.34E-01
	PM: Time to complete round	Inverse variance weighted	42.1124	18	1.07E-03
		MR Egger	37.8825	17	2.55E-03
	Reaction Time	Inverse variance weighted	13.9509	8	8.31E-02
		MR Egger	13.7923	7	5.50E-02
Number of live births	Cognitive performance	Inverse variance weighted	42.4382	8	1.12E-06
		MR Egger	33.0268	7	2.62E-05
	Fluid intelligence score	Inverse variance weighted	18.6047	9	2.88E-02
		MR Egger	18.2551	8	1.94E-02
	Memory Performance	Inverse variance weighted	4.7017	9	8.59E-01
		MR Egger	4.6975	8	7.89E-01
	TM: Interval in trail 2 path	Inverse variance weighted	17.8501	9	3.70E-02
		MR Egger	17.8267	8	2.26E-02
	TM: Duration to complete trail 2 path	Inverse variance weighted	14.7903	9	9.69E-02
		MR Egger	14.7621	8	6.39E-02
	SDS: Number of correct matches	Inverse variance weighted	21.6707	9	9.98E-03
		MR Egger	21.5332	8	5.86E-03
	SDS: Number of matches attempted	Inverse variance weighted	25.9138	9	2.11E-03
		MR Egger	25.8376	8	1.12E-03
	SDS: Duration to entering value	Inverse variance weighted	30.0585	9	4.29E-04
		MR Egger	29.7999	8	2.29E-04
	PM: Number of incorrect matches	Inverse variance weighted	18.6203	9	2.86E-02
		MR Egger	15.4546	8	5.09E-02
	PM: Time to complete round	Inverse variance weighted	28.4060	9	8.16E-04
		MR Egger	27.2452	8	6.41E-04
	Reaction Time	Inverse variance weighted	0.0771	1	7.81E-01
		MR Egger	/	/	/
Ever taken oral contraceptive pill	Cognitive performance	Inverse variance weighted	33.8809	23	6.69E-02
		MR Egger	30.0040	22	1.18E-01
	Fluid intelligence score	Inverse variance weighted	28.5218	23	1.97E-01
		MR Egger	28.3989	22	1.63E-01

	Memory Performance	Inverse variance weighted	20.6401	23	6.03E-01
		MR Egger	20.4782	22	5.53E-01
	TM: Interval in trail 2 path	Inverse variance weighted	24.8125	23	3.60E-01
		MR Egger	24.8058	22	3.06E-01
	TM: Duration to complete trail 2 path	Inverse variance weighted	26.9647	23	2.57E-01
		MR Egger	26.9325	22	2.14E-01
	SDS: Number of correct matches	Inverse variance weighted	33.7975	23	6.81E-02
		MR Egger	33.7340	22	5.23E-02
	SDS: Number of matches attempted	Inverse variance weighted	35.6967	23	4.43E-02
		MR Egger	35.5752	22	3.37E-02
	SDS: Duration to entering value	Inverse variance weighted	36.3438	23	3.80E-02
		MR Egger	36.1778	22	2.91E-02
	PM: Number of incorrect matches	Inverse variance weighted	42.0888	23	8.86E-03
		MR Egger	37.9496	22	1.86E-02
	PM: Time to complete round	Inverse variance weighted	34.7782	23	5.47E-02
		MR Egger	34.6227	22	4.24E-02
	Reaction Time	Inverse variance weighted	1.0566	6	9.83E-01
		MR Egger	0.8335	5	9.75E-01
Ever used hormone-replacement therapy	Cognitive performance	Inverse variance weighted	10.5675	5	6.07E-02
		MR Egger	9.0217	4	6.06E-02
	Fluid intelligence score	Inverse variance weighted	14.0925	6	2.86E-02
		MR Egger	11.9880	5	3.50E-02
	Memory Performance	Inverse variance weighted	11.3385	6	7.85E-02
		MR Egger	9.5022	5	9.06E-02
	TM: Interval in trail 2 path	Inverse variance weighted	14.1532	6	2.80E-02
		MR Egger	12.4458	5	2.92E-02
	TM: Duration to complete trail 2 path	Inverse variance weighted	13.7395	6	3.27E-02
		MR Egger	11.4503	5	4.31E-02
	SDS: Number of correct matches	Inverse variance weighted	24.1490	6	4.90E-04
		MR Egger	23.7662	5	2.41E-04
	SDS: Number of matches attempted	Inverse variance weighted	22.8756	6	8.39E-04
		MR Egger	22.4064	5	4.38E-04

	SDS: Duration to entering value	Inverse variance weighted	25.0365	6	3.36E-04
		MR Egger	24.7698	5	1.54E-04
	PM: Number of incorrect matches	Inverse variance weighted	17.4339	6	7.81E-03
		MR Egger	17.3273	5	3.92E-03
	PM: Time to complete round	Inverse variance weighted	8.1648	6	2.26E-01
		MR Egger	5.4277	5	3.66E-01
	Reaction Time	Inverse variance weighted	0.8142	1	3.67E-01
		MR Egger	/	/	/

Notes. MR: Mendelian Randomization; IVW: Inverse Variance Weighted; df: degrees of freedom; TM: Trail Making; SDS: Symbol Digit Substitution; PM: Pairs Matching.

Table S12. MR-Egger intercept tests for horizontal pleiotropy from MR analysis of reproductive traits and cognitive function.

Exposure	Outcome	Egger_intercept	SE	P
Age at menarche	Cognitive performance	-0.0023	0.0015	0.1083
	Fluid intelligence score	-0.0066	0.0034	0.0542
	Memory performance	-0.0003	0.0016	0.8672
	TM: Interval in trail 2 path	-0.0005	0.0016	0.7377
	TM: Duration to complete trail 2 path	-0.0007	0.0016	0.6525
	SDS: Number of correct matches	0.0012	0.0014	0.4066
	SDS: Number of matches attempted	0.0013	0.0015	0.3786
	SDS: Duration to entering value	-0.0003	0.0014	0.8495
	PM: Number of incorrect matches	0.0010	0.0008	0.1989
	PM: Time to complete round	0.0002	0.0009	0.8432
	Reaction Time	-0.0219	0.0094	0.0753
Age at menopause	Cognitive performance	0.0003	0.0012	0.7964
	Fluid intelligence score	-0.0002	0.0029	0.9430
	Memory performance	0.0006	0.0013	0.6799
	TM: Interval in trail 2 path	-0.0018	0.0011	0.1287
	TM: Duration to complete trail 2 path	-0.0016	0.0012	0.1814
	SDS: Number of correct matches	0.0021	0.0013	0.1082
	SDS: Number of matches attempted	0.0021	0.0013	0.1005
	SDS: Duration to entering value	-0.0019	0.0013	0.1382
	PM: Number of incorrect matches	-0.0006	0.0007	0.3844
	PM: Time to complete round	-0.0001	0.0008	0.8637
	Reaction Time	-0.0159	0.0094	0.0985
Age at first sexual intercourse	Cognitive performance	0.0006	0.0043	0.8875
	Fluid intelligence score	0.0049	0.0103	0.6360
	Memory performance	0.0013	0.0048	0.7797
	TM: Interval in trail 2 path	-0.0003	0.0050	0.9503
	TM: Duration to complete trail 2 path	-0.0012	0.0050	0.8185
	SDS: Number of correct matches	-0.0018	0.0052	0.7360
	SDS: Number of matches attempted	-0.0018	0.0054	0.7397
	SDS: Duration to entering value	-0.0013	0.0051	0.7996

	PM: Number of incorrect matches	-0.0039	0.0026	0.1410
	PM: Time to complete round	-0.0002	0.0030	0.9383
	Reaction Time	0.0124	0.0295	0.6778
Age at first birth	Cognitive performance	0.0035	0.0050	0.4831
	Fluid intelligence score	0.0053	0.0106	0.6192
	Memory performance	0.0007	0.0051	0.8859
	TM: Interval in trail 2 path	-0.0056	0.0041	0.1779
	TM: Duration to complete trail 2 path	-0.0082	0.0042	0.0563
	SDS: Number of correct matches	0.0061	0.0035	0.0902
	SDS: Number of matches attempted	0.0065	0.0037	0.0882
	SDS: Duration to entering value	-0.0066	0.0037	0.0778
	PM: Number of incorrect matches	-0.0017	0.0025	0.5036
	PM: Time to complete round	-0.0006	0.0027	0.8302
	Reaction Time	0.0320	0.0348	0.3671
Birth weight of first child	Cognitive performance	0.0062	0.0036	0.0944
	Fluid intelligence score	0.0122	0.0091	0.1885
	Memory performance	-0.0006	0.0041	0.8767
	TM: Interval in trail 2 path	0.0009	0.0040	0.8242
	TM: Duration to complete trail 2 path	0.0011	0.0043	0.7982
	SDS: Number of correct matches	-0.0011	0.0035	0.7584
	SDS: Number of matches attempted	-0.0011	0.0035	0.7496
	SDS: Duration to entering value	0.0006	0.0033	0.8669
	PM: Number of incorrect matches	0.0004	0.0023	0.8540
	PM: Time to complete round	0.0012	0.0027	0.6716
	Reaction Time	-0.0245	0.0313	0.4453
Medical abortion	Cognitive performance	0.0010	0.0023	0.6786
	Fluid intelligence score	-0.0019	0.0055	0.7281
	Memory performance	0.0015	0.0027	0.5802
	TM: Interval in trail 2 path	-0.0012	0.0027	0.6710
	TM: Duration to complete trail 2 path	-0.0014	0.0029	0.6356
	SDS: Number of correct matches	0.0019	0.0024	0.4346
	SDS: Number of matches attempted	0.0022	0.0024	0.3651

	SDS: Duration to entering value	-0.0003	0.0022	0.9107
	PM: Number of incorrect matches	-0.0025	0.0010	0.2143
	PM: Time to complete round	-0.0021	0.0015	0.1861
	Reaction Time	-0.0162	0.0570	0.7848
Number of live births	Cognitive performance	-0.0324	0.0229	0.2007
	Fluid intelligence score	-0.0148	0.0377	0.7057
	Memory performance	0.0011	0.0176	0.9500
	TM: Interval in trail 2 path	-0.0022	0.0212	0.9208
	TM: Duration to complete trail 2 path	0.0025	0.0199	0.9048
	SDS: Number of correct matches	0.0051	0.0225	0.8268
	SDS: Number of matches attempted	0.0038	0.0247	0.8817
	SDS: Duration to entering value	-0.0070	0.0264	0.7989
	PM: Number of incorrect matches	-0.0119	0.0093	0.2364
	PM: Time to complete round	-0.0073	0.0125	0.5754
	Reaction Time	/	/	/
Ever taken oral contraceptive pill	Cognitive performance	0.0045	0.0026	0.1059
	Fluid intelligence score	0.0022	0.0070	0.7605
	Memory performance	0.0018	0.0044	0.6913
	TM: Interval in trail 2 path	0.0003	0.0037	0.9394
	TM: Duration to complete trail 2 path	0.0007	0.0040	0.8726
	SDS: Number of correct matches	-0.0009	0.0042	0.8406
	SDS: Number of matches attempted	-0.0012	0.0043	0.7865
	SDS: Duration to entering value	0.0014	0.0043	0.7537
	PM: Number of incorrect matches	-0.0034	0.0022	0.1356
	PM: Time to complete round	-0.0007	0.0021	0.7562
	Reaction Time	-0.0654	0.1386	0.6566
Ever used hormone-replacement therapy	Cognitive performance	-0.0038	0.0045	0.4543
	Fluid intelligence score	-0.0116	0.0124	0.3918
	Memory performance	0.0076	0.0077	0.3708
	TM: Interval in trail 2 path	0.0060	0.0072	0.4453
	TM: Duration to complete trail 2 path	0.0071	0.0071	0.3633
	SDS: Number of correct matches	-0.0027	0.0096	0.7880

	SDS: Number of matches attempted	-0.0030	0.0093	0.7593
	SDS: Duration to entering value	0.0023	0.0098	0.8257
	PM: Number of incorrect matches	-0.0007	0.0040	0.8677
	PM: Time to complete round	0.0036	0.0023	0.1732
	Reaction Time	/	/	/

Notes. MR: Mendelian Randomization; SE: Standard Error; TM: Trail Making; SDS: Symbol Digit Substitution; PM: Pairs Matching.

Table S13. The results of MR analyses using the Outlier-corrected method in MR-PRESSO with the results of the original MR analyses.

Exposure	Outcome	MR Analysis	Causal Estimate	Sd	T-stat	P-value	Global Test$P-value	Distortion Test$P-value
Age at menarche	Cognitive performance	Raw	-0.0170	0.0126	-1.3509	0.1784	<0.0003	0.6563
		Outlier-corrected	-0.0136	0.0102	-1.3343	0.1839		
	Fluid intelligence score	Raw	-0.0438	0.0295	-1.4857	0.1391	<0.0003	0.3123
		Outlier-corrected	-0.0287	0.0257	-1.1177	0.2653		
	Memory performance	Raw	-0.0134	0.0136	-0.9846	0.3261	0.0023	0.8037
		Outlier-corrected	-0.0109	0.0132	-0.8284	0.4085		
	TM: Interval in trail 2 path	Raw	-0.0126	0.0135	-0.9311	0.3531	<0.0003	0.8620
		Outlier-corrected	-0.0153	0.0130	-1.1705	0.2434		
	TM: Duration to complete trail 2 path	Raw	-0.0149	-0.0199	-1.0828	0.2803	<0.0003	0.7600
		Outlier-corrected	-0.0199	0.0131	-1.5253	0.1290		
	SDS: Number of correct matches	Raw	-0.0062	0.0123	-0.5004	0.6174	<0.0003	0.0257
		Outlier-corrected	0.0004	0.0118	0.0348	0.9723		
	SDS: Number of matches attempted	Raw	-0.0042	0.0126	-0.3380	0.7358	<0.0003	0.1083
		Outlier-corrected	0.0022	0.0120	0.1861	0.8526		
	SDS: Duration to entering value	Raw	0.0103	0.0121	0.8535	0.3945	<0.0003	0.3873
		Outlier-corrected	0.0061	0.0113	0.5386	0.5908		
	PM: Number of incorrect matches	Raw	-0.0068	0.0065	-1.0336	0.3027	<0.0003	0.8670
		Outlier-corrected	-0.0080	0.0060	-1.3293	0.1854		
	PM: Time to complete round	Raw	-0.0018	0.0074	-0.2478	0.8046	<0.0003	0.9293
		Outlier-corrected	-0.0014	0.0068	-0.2025	0.8398		
	Reaction Time	Raw	0.1434	0.0871	1.6468	0.1030	0.1887	/
		Outlier-corrected	NA	NA	NA	NA		
Age at menopause	Cognitive performance	Raw	0.0085	0.0143	0.5953	0.5529	<0.0003	0.6937
		Outlier-corrected	0.0146	0.0113	1.2909	0.1995		
	Fluid intelligence score	Raw	0.0026	0.0339	0.0782	0.9378	<0.0003	0.8690
		Outlier-corrected	0.0098	0.0273	0.3605	0.7192		
	Memory performance	Raw	-0.0294	0.0159	-1.8447	0.0677	0.0650	/
		Outlier-corrected	NA	NA	NA	NA		
	TM: Interval in trail 2 path	Raw	-0.0011	0.0138	-0.0825	0.9344	0.0040	/
		Outlier-corrected	NA	NA	NA	NA		

	TM: Duration to complete trail 2 path	Raw	-0.0069	0.0141	-0.4868	0.6274	0.0067	/
		Outlier-corrected	NA	NA	NA	NA		
	SDS: Number of correct matches	Raw	0.0005	0.0153	0.0339	0.9730	<0.0003	0.0563
		Outlier-corrected	-0.0021	0.0148	-0.1388	0.8899		
	SDS: Number of matches attempted	Raw	0.0039	0.0153	0.2541	0.7999	<0.0003	0.6343
		Outlier-corrected	0.0022	0.0142	0.1543	0.8776		
	SDS: Duration to entering value	Raw	-0.0030	0.0155	-0.1945	0.8461	<0.0003	0.9937
		Outlier-corrected	-0.0029	0.0143	-0.2013	0.8408		
	PM: Number of incorrect matches	Raw	0.0030	0.0083	0.3547	0.7235	<0.0003	0.1763
		Outlier-corrected	-0.0024	0.0068	-0.3516	0.7258		
	PM: Time to complete round	Raw	0.0031	0.0096	0.3194	0.7500	<0.0003	0.8707
		Outlier-corrected	0.0045	0.0080	0.5590	0.5773		
	Reaction Time	Raw	-0.0134	0.1058	-0.1264	0.8999	0.8497	/
		Outlier-corrected	NA	NA	NA	NA		
Age at first sexual intercourse	Cognitive performance	Raw	0.2796	0.0422	6.6229	<0.0001	<0.0003	0.4610
		Outlier-corrected	0.2558	0.0381	6.7132	<0.0001		
	Fluid intelligence score	Raw	0.6278	0.1019	6.1618	<0.0001	<0.0003	0.9803
		Outlier-corrected	0.6258	0.0860	7.2789	<0.0001		
	Memory performance	Raw	0.0885	0.0469	1.8874	0.0641	0.0237	0.5920
		Outlier-corrected	0.1168	0.0445	2.6222	0.0112		
	TM: Interval in trail 2 path	Raw	-0.1564	0.0495	-3.1613	0.0025	<0.0003	0.6553
		Outlier-corrected	-0.1378	0.0462	-2.9791	0.0042		
	TM: Duration to complete trail 2 path	Raw	-0.1484	0.0495	-2.9978	0.0040	<0.0003	0.6557
		Outlier-corrected	-0.1311	0.0469	-2.7971	0.0070		
	SDS: Number of correct matches	Raw	0.1051	0.0515	2.0381	0.0461	<0.0003	0.7850
		Outlier-corrected	0.1182	0.0446	2.6519	0.0461		
	SDS: Number of matches attempted	Raw	0.0945	0.0533	1.7718	0.0817	<0.0003	0.7790
		Outlier-corrected	0.1081	0.0461	2.3457	0.0227		
	SDS: Duration to entering value	Raw	-0.0853	0.0501	-1.7018	0.0941	<0.0003	0.5403
		Outlier-corrected	-0.0669	0.0435	-1.5378	0.1299		
	PM: Number of incorrect matches	Raw	-0.0232	0.0261	-0.8887	0.3778	<0.0003	0.0423
		Outlier-corrected	-0.0021	0.0234	-0.0898	0.9287		

	PM: Time to complete round	Raw	-0.0056	0.0296	-0.1880	0.8515	<0.0003	0.1817
		Outlier-corrected	0.0094	0.0225	0.4182	0.6775		
	Reaction Time	Raw	-0.4030	0.2897	-1.3912	0.1764	0.4543	/
		Outlier-corrected	NA	NA	NA	NA		
Age at first birth	Cognitive performance	Raw	0.1165	0.0149	7.8079	<0.0001	<0.0003	0.1703
		Outlier-corrected	0.1038	0.0120	8.6775	<0.0001		
	Fluid intelligence score	Raw	0.2476	0.0315	7.8542	<0.0001	<0.0003	0.2517
		Outlier-corrected	0.2224	0.0267	8.3294	<0.0001		
	Memory performance	Raw	0.0418	0.0151	2.7623	0.0080	0.0030	/
		Outlier-corrected	NA	NA	NA	NA		
	TM: Interval in trail 2 path	Raw	-0.0594	0.0125	-4.7572	<0.0001	0.0013	/
		Outlier-corrected	NA	NA	NA	NA		
	TM: Duration to complete trail 2 path	Raw	-0.0555	0.0129	-4.2828	0.0001	0.0007	/
		Outlier-corrected	NA	NA	NA	NA		
	SDS: Number of correct matches	Raw	0.0520	0.0107	4.8415	<0.0001	0.0517	/
		Outlier-corrected	NA	NA	NA	NA		
	SDS: Number of matches attempted	Raw	0.0474	0.0114	4.1549	0.0001	0.0080	/
		Outlier-corrected	NA	NA	NA	NA		
	SDS: Duration to entering value	Raw	-0.0466	0.0112	-4.1488	0.0001	0.0143	/
		Outlier-corrected	NA	NA	NA	NA		
	PM: Number of incorrect matches	Raw	-0.0019	0.0074	-0.2583	0.7972	<0.0003	0.7677
		Outlier-corrected	-0.0049	0.0064	-0.7563	0.4533		
	PM: Time to complete round	Raw	0.0068	0.0080	0.8512	0.3987	<0.0003	0.2130
		Outlier-corrected	0.0024	0.0062	0.3884	0.6995		
	Reaction Time	Raw	-0.1198	0.1020	-1.1740	0.2524	0.1007	/
		Outlier-corrected	NA	NA	NA	NA		
Birth weight of first child	Cognitive performance	Raw	0.0541	0.0282	1.9164	0.0623	<0.0003	0.0337
		Outlier-corrected	0.0213	0.0199	1.0706	0.2911		
	Fluid intelligence score	Raw	0.1210	0.0703	1.7221	0.0922	<0.0003	0.0430
		Outlier-corrected	0.0463	0.0492	0.9410	0.3526		
	Memory performance	Raw	0.0249	0.0308	0.8102	0.4223	0.0293	/
		Outlier-corrected	NA	NA	NA	NA		

	TM: Interval in trail 2 path	Raw	0.0210	0.0302	0.6965	0.4899	<0.0003	0.8020
		Outlier-corrected	0.0296	0.0288	1.0282	0.3098		
	TM: Duration to complete trail 2 path	Raw	0.0129	0.0320	0.4033	0.6887	<0.0003	0.9547
		Outlier-corrected	0.0115	0.0289	0.3975	0.6931		
	SDS: Number of correct matches	Raw	-0.0104	0.0260	-0.4017	0.6899	0.0037	0.0987
		Outlier-corrected	-0.0024	0.0243	-0.1005	0.9205		
	SDS: Number of matches attempted	Raw	-0.0167	0.0265	-0.6306	0.5317	0.0017	0.3657
		Outlier-corrected	-0.0086	0.0248	-0.3469	0.7304		
	SDS: Duration to entering value	Raw	0.0166	0.0249	0.6664	0.5087	0.0137	/
		Outlier-corrected	NA	NA	NA	NA		
	PM: Number of incorrect matches	Raw	-0.0071	0.0172	-0.4143	0.6807	<0.0003	0.5300
		Outlier-corrected	-0.0210	0.0114	-1.8531	0.0715		
	PM: Time to complete round	Raw	-0.0035	0.0205	-0.1706	0.8654	<0.0003	0.5150
		Outlier-corrected	-0.0286	0.0133	-2.1461	0.0380		
	Reaction Time	Raw	0.2948	0.1786	1.6512	0.1171	0.6120	/
		Outlier-corrected	NA	NA	NA	NA		
Medical abortion	Cognitive performance	Raw	-0.0102	0.0148	-0.6870	0.5014	0.0020	/
		Outlier-corrected	NA	NA	NA	NA		
	Fluid intelligence score	Raw	-0.0180	0.0353	-0.5093	0.6167	0.0120	/
		Outlier-corrected	NA	NA	NA	NA		
	Memory performance	Raw	-0.0132	0.0178	-0.7408	0.4684	0.4310	/
		Outlier-corrected	NA	NA	NA	NA		
	TM: Interval in trail 2 path	Raw	-0.0002	0.0176	-0.0098	0.9923	0.0777	/
		Outlier-corrected	NA	NA	NA	NA		
	TM: Duration to complete trail 2 path	Raw	0.0103	0.0185	0.5580	0.5837	0.0630	/
		Outlier-corrected	NA	NA	NA	NA		
	SDS: Number of correct matches	Raw	-0.0309	0.0155	-1.9903	0.0620	0.2357	/
		Outlier-corrected	NA	NA	NA	NA		
	SDS: Number of matches attempted	Raw	-0.0307	0.0158	-1.9394	0.0683	0.1997	/
		Outlier-corrected	NA	NA	NA	NA		
	SDS: Duration to entering value	Raw	0.0229	0.0143	1.5998	0.1271	0.3773	/
		Outlier-corrected	NA	NA	NA	NA		

	PM: Number of incorrect matches	Raw	-0.0015	0.0074	-0.2001	0.8436	0.2350	/
		Outlier-corrected	NA	NA	NA	NA		
	PM: Time to complete round	Raw	-0.0082	0.0103	-0.7935	0.4378	0.0033	0.1680
		Outlier-corrected	-0.0023	0.0084	-0.2792	0.7835		
	Reaction Time	Raw	0.0162	0.2143	0.0757	0.9415	0.1003	/
		Outlier-corrected	NA	NA	NA	NA		
Number of live births	Cognitive performance	Raw	-0.2074	0.1047	-1.9808	0.0830	<0.0003	0.9999
		Outlier-corrected	-0.3577	0.0441	-8.1126	0.0013		
	Fluid intelligence score	Raw	-0.6545	0.1648	-3.9708	0.0033	0.0597	/
		Outlier-corrected	NA	NA	NA	NA		
	Memory performance	Raw	-0.0751	0.0585	-1.2844	0.2311	0.8510	/
		Outlier-corrected	NA	NA	NA	NA		
	TM: Interval in trail 2 path	Raw	0.1104	0.0920	1.1994	0.2610	0.0403	0.4227
		Outlier-corrected	0.1878	0.0709	2.6472	0.0294		
	TM: Duration to complete trail 2 path	Raw	0.0735	0.0863	0.8518	0.4164	0.0907	/
		Outlier-corrected	NA	NA	NA	NA		
	SDS: Number of correct matches	Raw	-0.2270	0.0979	-2.3196	0.0455	0.0140	0.1013
		Outlier-corrected	-0.1516	0.0750	-2.0218	0.0778		
	SDS: Number of matches attempted	Raw	-0.2034	0.1070	-1.9006	0.0898	0.0050	0.1440
		Outlier-corrected	-0.1263	0.0877	-1.4393	0.1880		
	SDS: Duration to entering value	Raw	0.2267	0.1149	1.9723	0.0800	0.0010	0.7753
		Outlier-corrected	0.2097	0.0821	2.5531	0.0379		
	PM: Number of incorrect matches	Raw	0.0989	0.0443	2.2338	0.0524	0.0393	0.4117
		Outlier-corrected	0.1301	0.0320	4.0655	0.0036		
	PM: Time to complete round	Raw	0.0304	0.0553	0.5496	0.5960	0.0003	0.6787
		Outlier-corrected	0.0444	0.0321	1.3820	0.2095		
	Reaction Time	Raw	NA	NA	NA	NA	Not enough intrumental variables	
		Outlier-corrected	NA	NA	NA	NA		
Ever taken oral contraceptive pill	Cognitive performance	Raw	0.2507	0.1360	1.8441	0.0781	0.0747	/
		Outlier-corrected	NA	NA	NA	NA		
	Fluid intelligence score	Raw	0.7854	0.3355	2.3408	0.0283	0.2140	/
		Outlier-corrected	NA	NA	NA	NA		

	Memory performance	Raw	0.1454	0.2017	0.7205	0.4785	0.5910	/
		Outlier-corrected	NA	NA	NA	NA		
	TM: Interval in trail 2 path	Raw	-0.1685	0.1787	-0.9427	0.3556	0.3607	/
		Outlier-corrected	NA	NA	NA	NA		
	TM: Duration to complete trail 2 path	Raw	-0.1280	0.1918	-0.6675	0.5111	0.2467	/
		Outlier-corrected	NA	NA	NA	NA		
	SDS: Number of correct matches	Raw	0.2738	0.2013	1.3600	0.1870	0.0627	/
		Outlier-corrected	NA	NA	NA	NA		
	SDS: Number of matches attempted	Raw	0.2335	0.2068	1.1289	0.2706	0.0427	/
		Outlier-corrected	NA	NA	NA	NA		
	SDS: Duration to entering value	Raw	-0.3495	0.2082	-1.6788	0.1067	0.0410	0.3200
		Outlier-corrected	-0.2378	0.1868	-1.2732	0.2163		
	PM: Number of incorrect matches	Raw	-0.1998	0.1096	-1.8238	0.0812	0.0103	/
		Outlier-corrected	NA	NA	NA	NA		
	PM: Time to complete round	Raw	-0.3919	0.1008	-3.8876	0.0007	0.0640	/
		Outlier-corrected	NA	NA	NA	NA		
	Reaction Time	Raw	1.6402	0.8906	1.8417	0.1151	0.9797	/
		Outlier-corrected	NA	NA	NA	NA		
Ever used hormone-replacement therapy	Cognitive performance	Raw	-0.1670	0.1526	-1.0944	0.3237	0.0890	/
		Outlier-corrected	NA	NA	NA	NA		
	Fluid intelligence score	Raw	-0.3406	0.4109	-0.8289	0.4389	0.0447	0.4430
		Outlier-corrected	-0.7359	0.4239	-1.7362	0.1431		
	Memory performance	Raw	0.0906	0.2592	0.3493	0.7388	0.1130	/
		Outlier-corrected	NA	NA	NA	NA		
	TM: Interval in trail 2 path	Raw	0.3939	0.2354	1.6735	0.1453	0.0540	/
		Outlier-corrected	NA	NA	NA	NA		
	TM: Duration to complete trail 2 path	Raw	0.4329	0.2387	1.8135	0.1197	0.0543	/
		Outlier-corrected	NA	NA	NA	NA		
	SDS: Number of correct matches	Raw	-0.5888	0.2968	-1.9838	0.0945	0.0040	0.4333
		Outlier-corrected	-0.4566	0.2664	-1.7139	0.1472		
	SDS: Number of matches attempted	Raw	-0.5852	0.2888	-2.0262	0.0891	0.0050	0.4333
		Outlier-corrected	-0.4586	0.2617	-1.7519	0.1402		

	SDS: Duration to entering value	Raw	0.5153	0.3013	1.7103	0.1381	0.0030	0.9293
		Outlier-corrected	0.5211	0.2496	2.0881	0.1051		
	PM: Number of incorrect matches	Raw	0.1907	0.1227	1.5543	0.1711	0.0200	0.2613
		Outlier-corrected	0.1291	0.1062	1.2155	0.2784		
	PM: Time to complete round	Raw	0.2335	0.0850	2.7482	0.0334	0.3233	/
		Outlier-corrected	NA	NA	NA	NA		
	Reaction Time	Raw	NA	NA	NA	NA	Not enough intrumental variables	
		Outlier-corrected	NA	NA	NA	NA		

Notes. MR: Mendelian Randomization; MR-PRESSO: Mendelian Randomization Pleiotropy RESidual Sum and Outlier; SE: Standard Error; TM: Trail Making; SDS: Symbol Digit Substitution; PM: Pairs Matching.

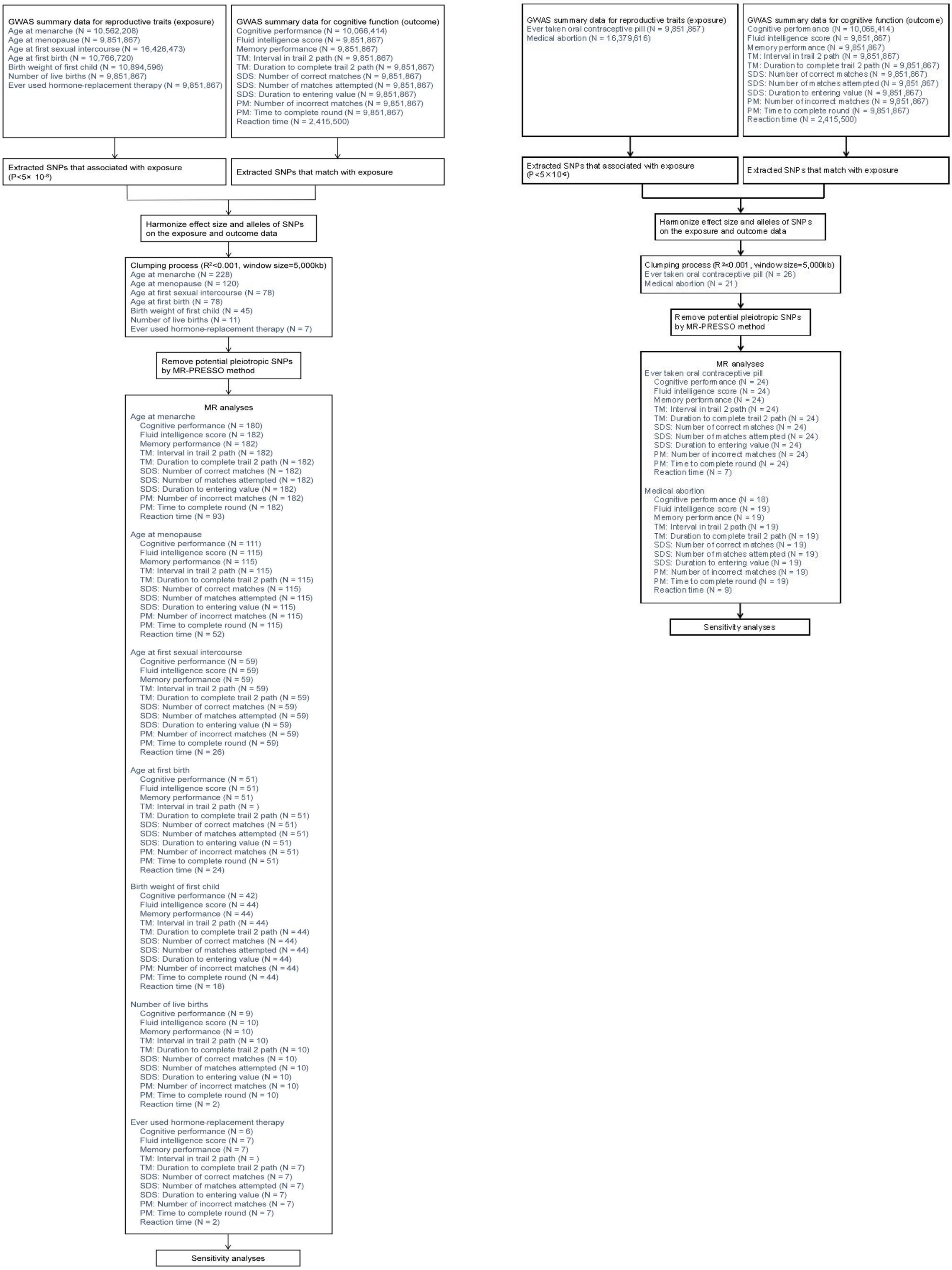

Figure S1 Study workflow IV selection for analysis of the effect of reproductive traits on cognitive function.

Figure S2. Scatter plot of SNPs impacts on reproductive traits and cognitive function.

Exposure: Age at menarche

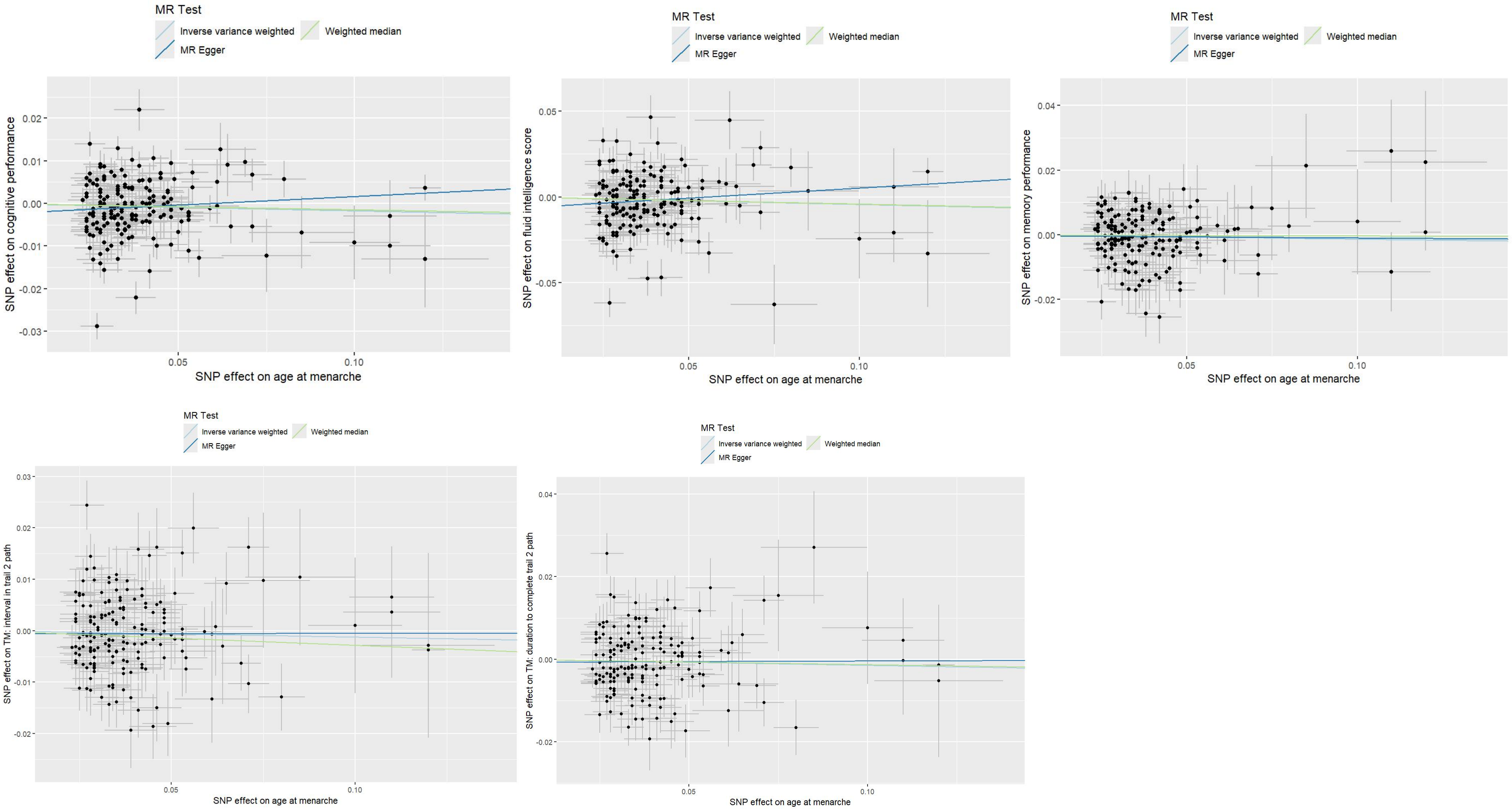

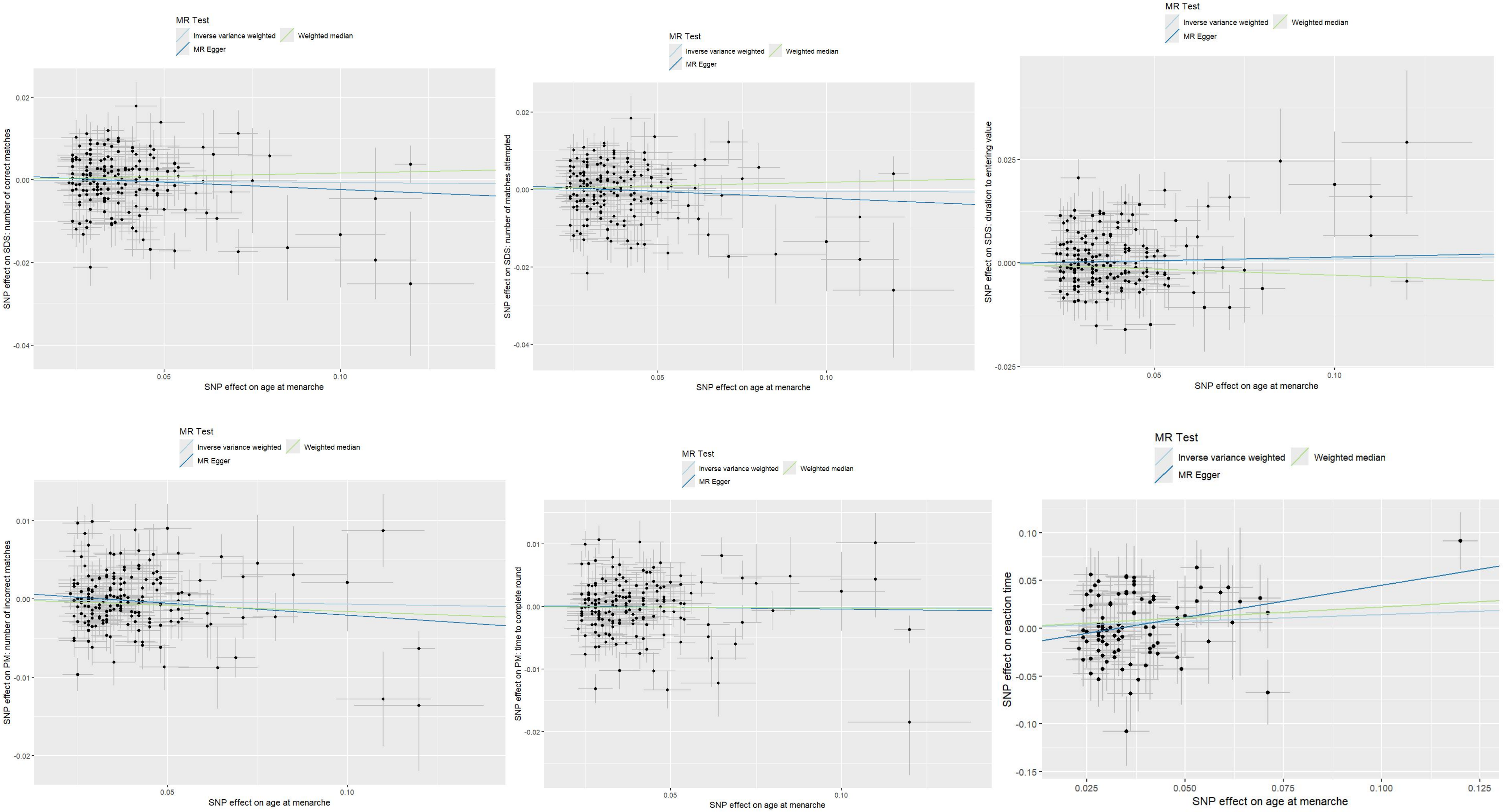
MR Test
Inverse variance weighted
Weighted median
MR Egger
SNP effect on SDS: number of correct matches
SNP effect on age at menarche
0.02
0.00
-0.02
-0.04
0.05
0.10
MR Test
Inverse variance weighted
Weighted median
MR Egger
SNP effect on SDS: number of matches attempted
SNP effect on age at menarche
0.02
0.00
-0.02
-0.04
0.05
0.10
MR Test
Inverse variance weighted
Weighted median
MR Egger
SNP effect on SDS: duration to entering value
SNP effect on age at menarche
0.025
0.000
-0.025
0.05
0.10
MR Test
Inverse variance weighted
Weighted median
MR Egger
SNP effect on PM: number of incorrect matches
SNP effect on age at menarche
0.01
0.00
-0.01
-0.02
0.05
0.10
MR Test
Inverse variance weighted
Weighted median
MR Egger
SNP effect on PM: time to complete round
SNP effect on age at menarche
0.01
0.00
-0.01
-0.02
0.05
0.10
MR Test
Inverse variance weighted
Weighted median
MR Egger
SNP effect on reaction time
SNP effect on age at menarche
0.10
0.05
0.00
-0.05
-0.10
-0.15
0.025
0.050
0.075
0.100
0.125

Exposure: Age at menopause

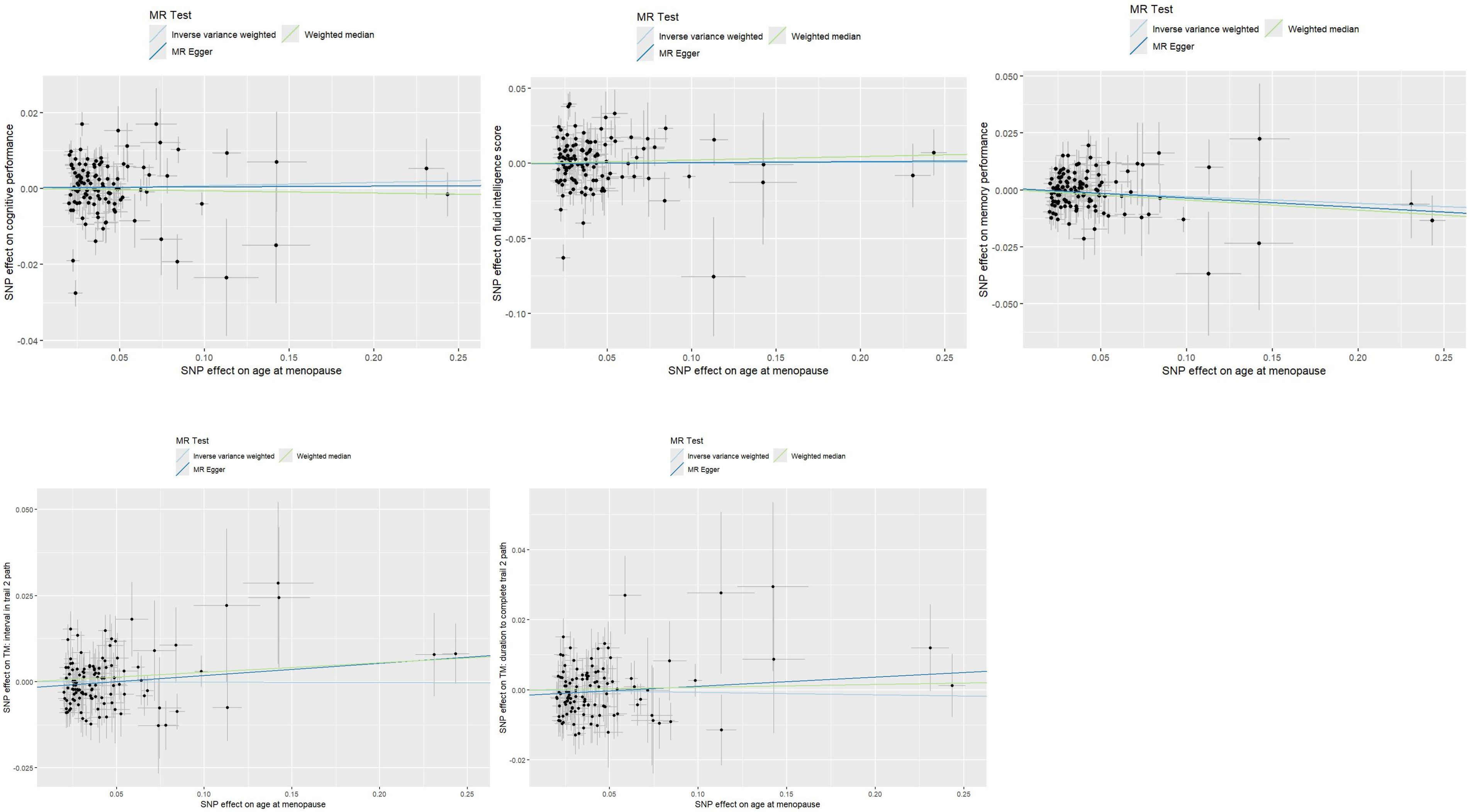

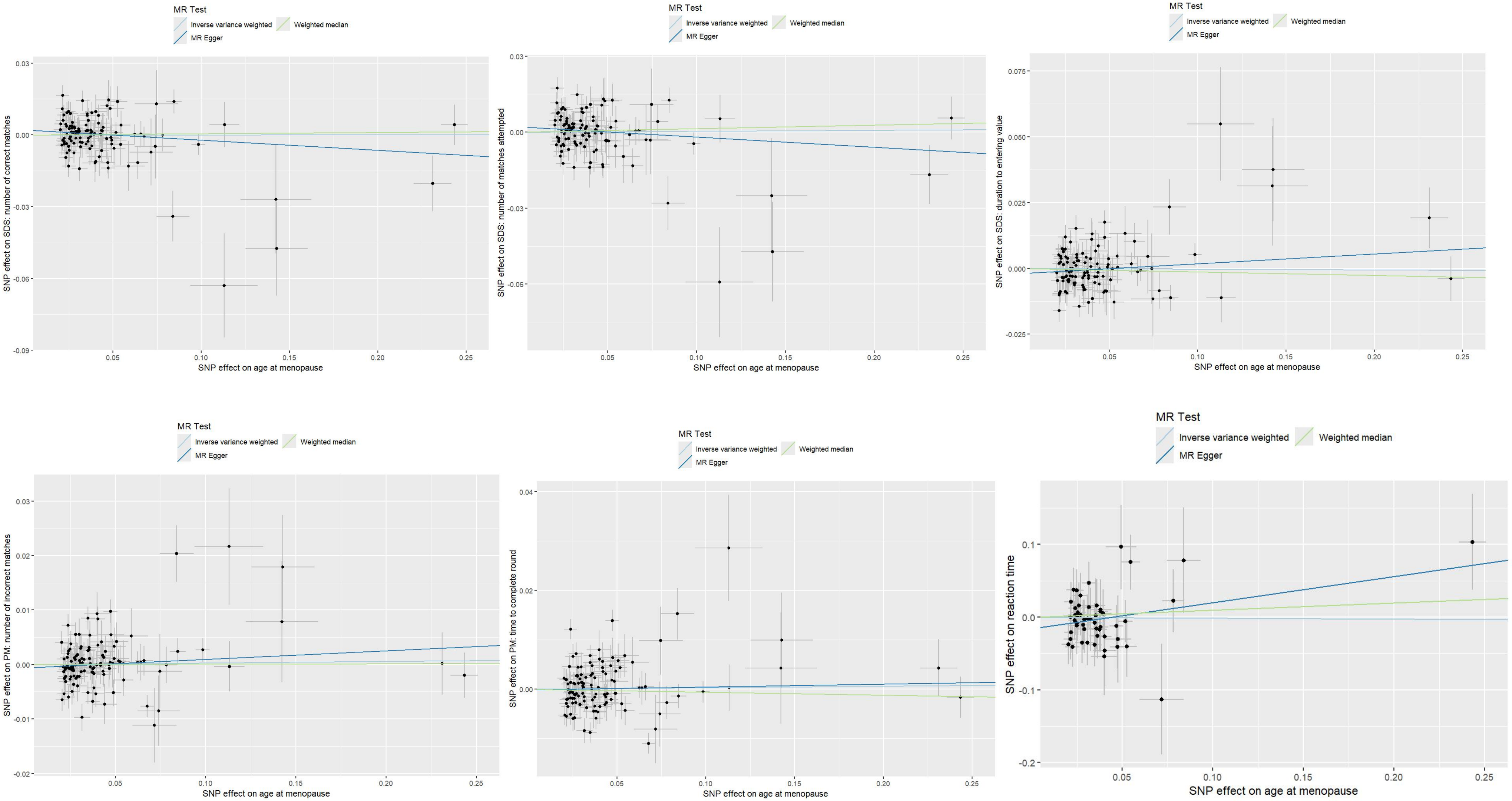
MR Test
Inverse variance weighted
Weighted median
MR Egger
SNP effect on SDS: number of correct matches
SNP effect on age at menopause
MR Test
Inverse variance weighted
Weighted median
MR Egger
SNP effect on SDS: number of matches attempted
SNP effect on age at menopause
MR Test
Inverse variance weighted
Weighted median
MR Egger
SNP effect on SDS: duration to entering value
SNP effect on age at menopause
MR Test
Inverse variance weighted
Weighted median
MR Egger
SNP effect on PM: number of incorrect matches
SNP effect on age at menopause
MR Test
Inverse variance weighted
Weighted median
MR Egger
SNP effect on PM: time to complete round
SNP effect on age at menopause
MR Test
Inverse variance weighted
Weighted median
MR Egger
SNP effect on reaction time
SNP effect on age at menopause

Exposure: Age at first sexual intercourse

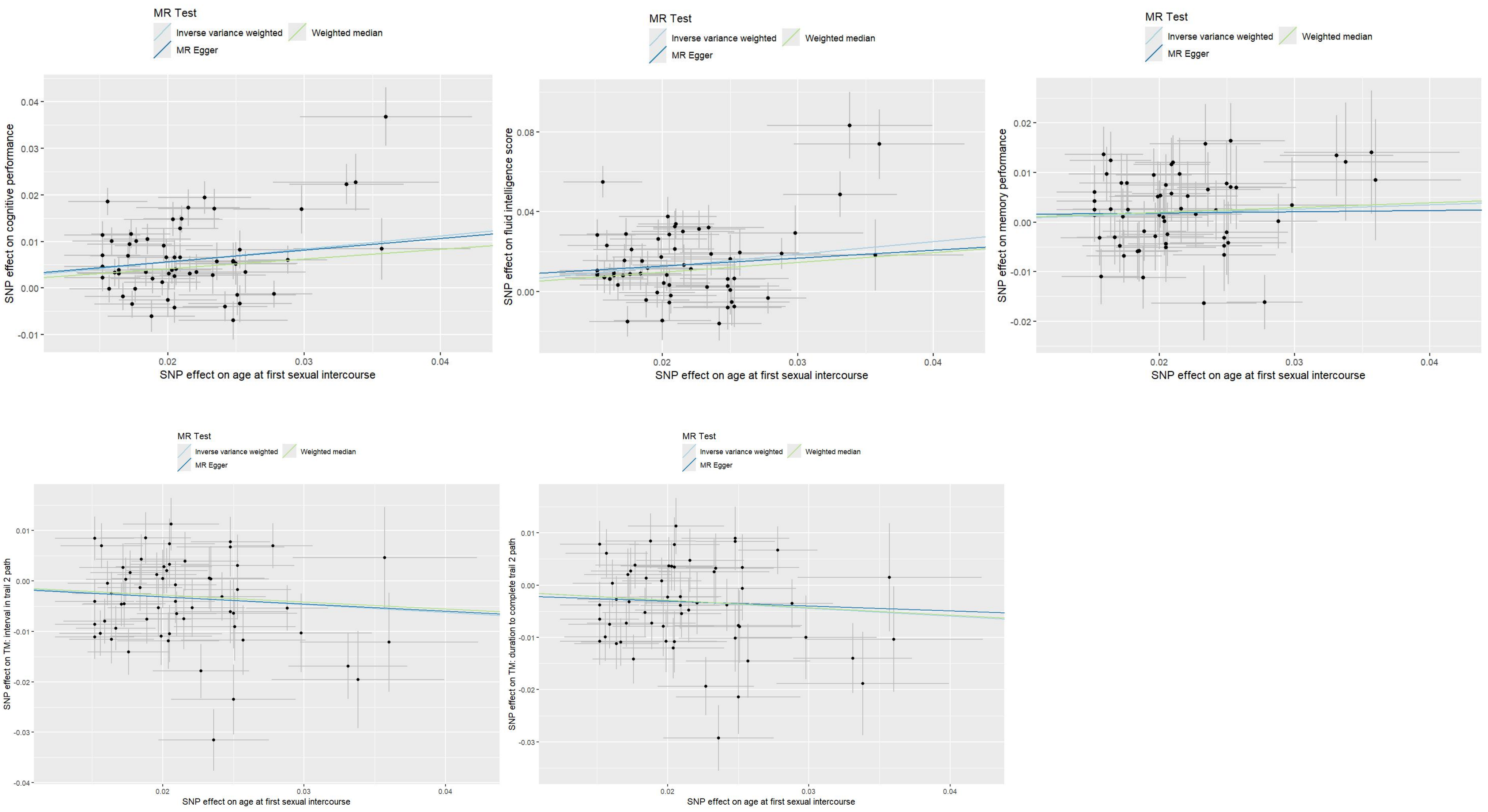

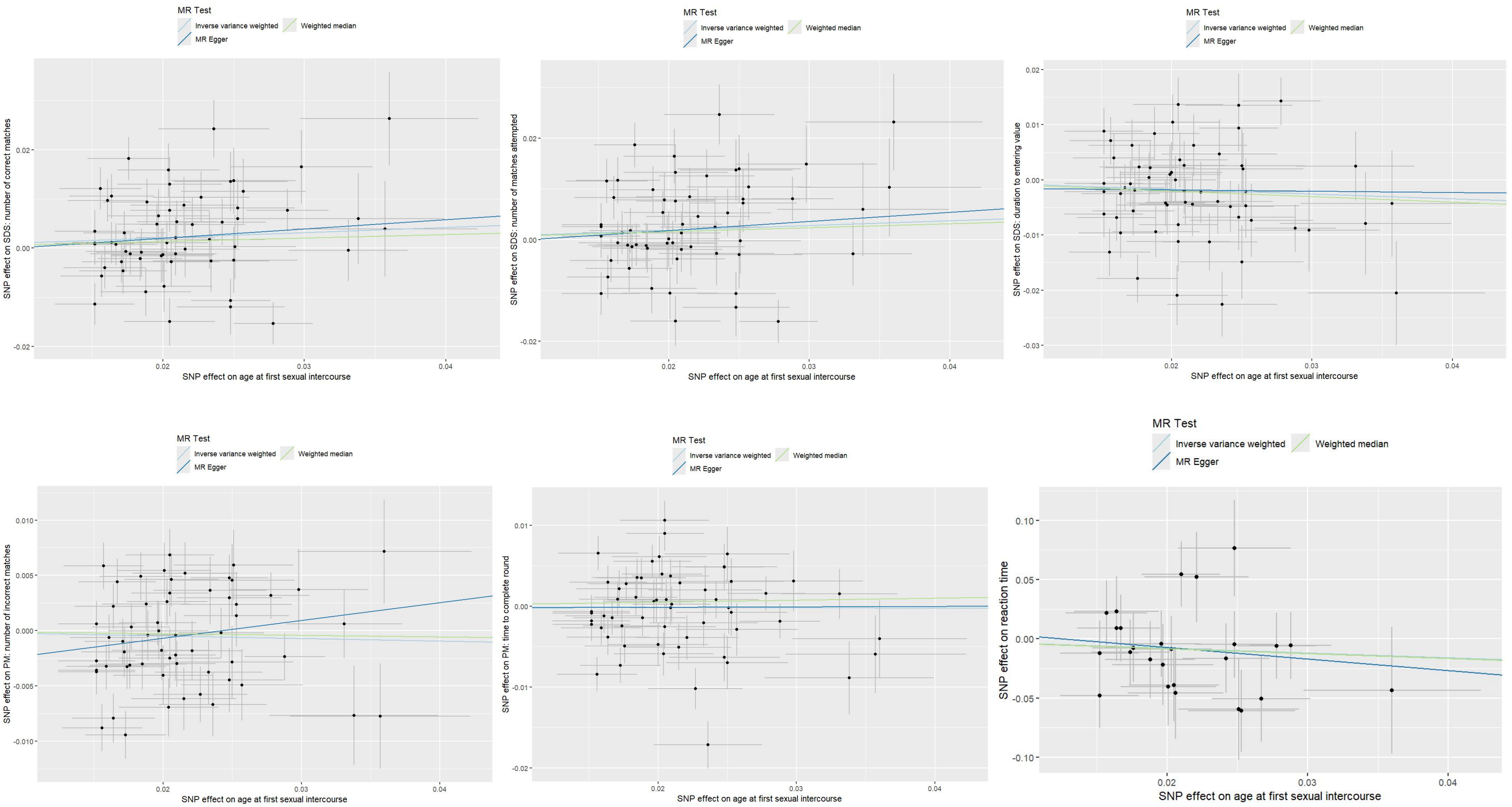

MR Test
Inverse variance weighted
Weighted median
MR Egger
SNP effect on SDS: number of correct matches
SNP effect on age at first sexual intercourse
SNP effect on SDS: number of matches attempted
SNP effect on SDS: duration to entering value
SNP effect on PM: number of incorrect matches
SNP effect on PM: time to complete round
SNP effect on reaction time

Exposure: Age at first birth

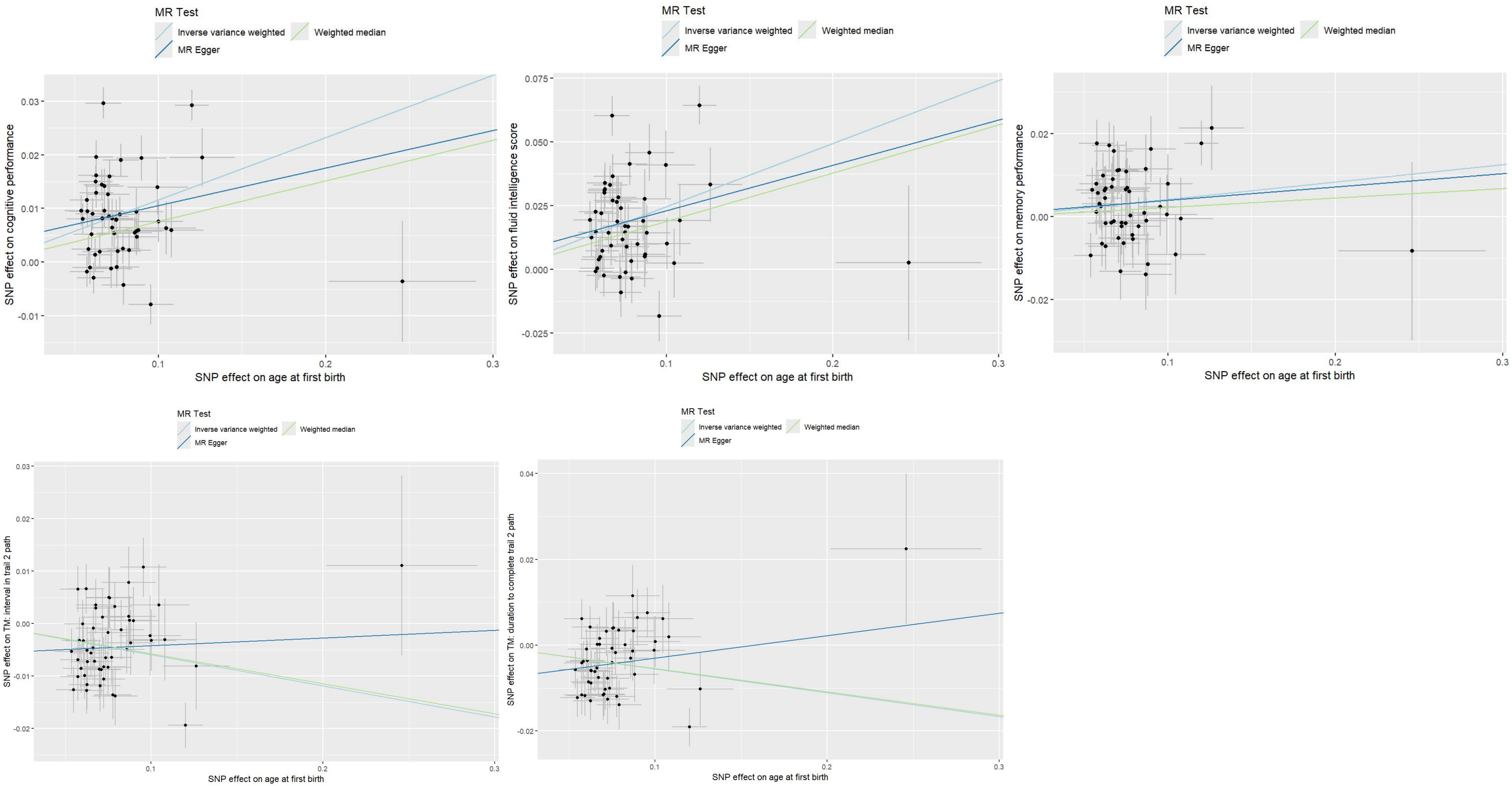

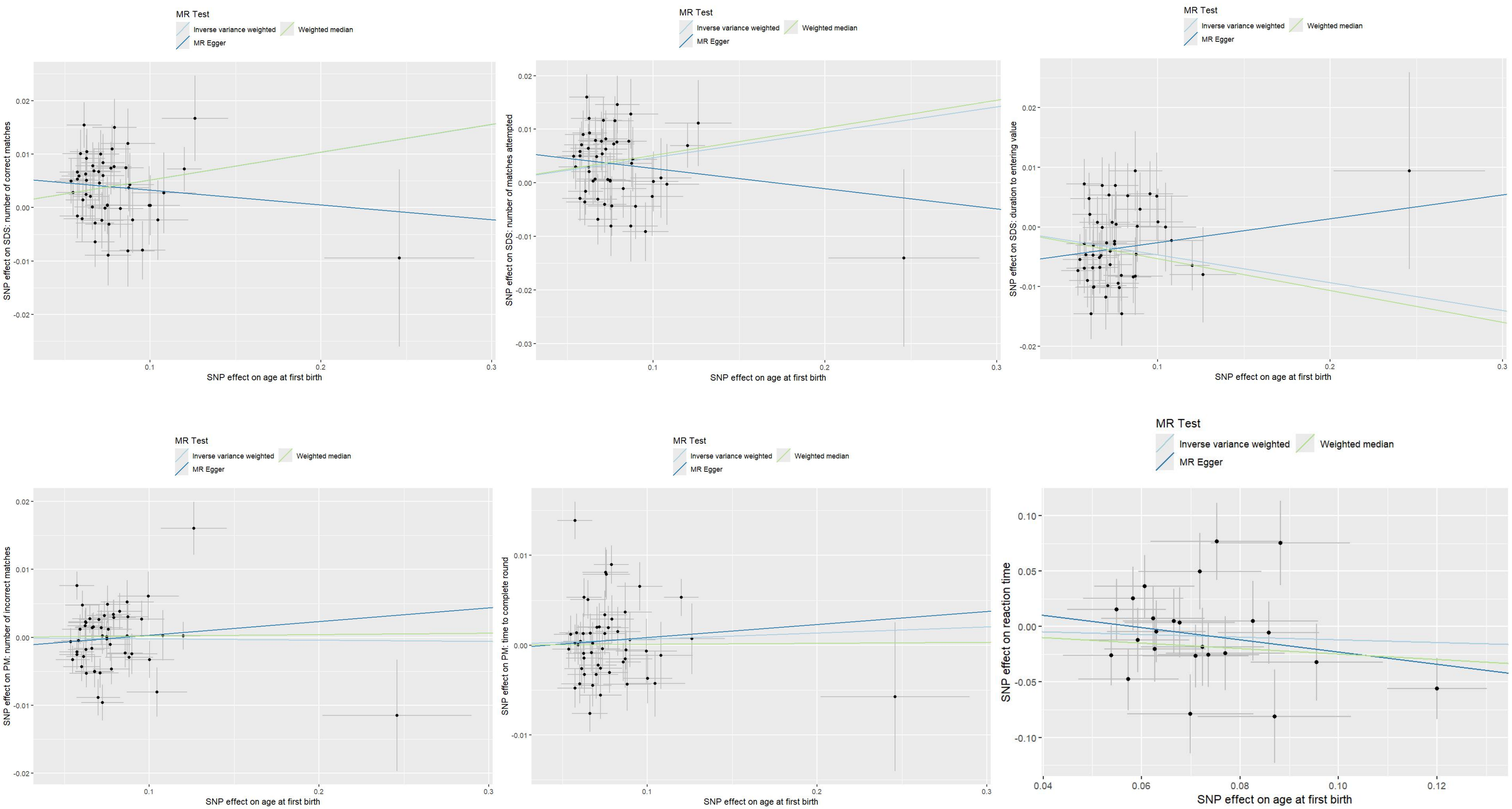
MR Test
Inverse variance weighted
Weighted median
MR Egger
SNP effect on SDS: number of correct matches
SNP effect on age at first birth
MR Test
Inverse variance weighted
Weighted median
MR Egger
SNP effect on SDS: number of matches attempted
SNP effect on age at first birth
MR Test
Inverse variance weighted
Weighted median
MR Egger
SNP effect on SDS: duration to entering value
SNP effect on age at first birth
MR Test
Inverse variance weighted
Weighted median
MR Egger
SNP effect on PM: number of incorrect matches
SNP effect on age at first birth
MR Test
Inverse variance weighted
Weighted median
MR Egger
SNP effect on PM: time to complete round
SNP effect on age at first birth
MR Test
Inverse variance weighted
Weighted median
MR Egger
SNP effect on reaction time
SNP effect on age at first birth

Exposure: Birth weight of first child

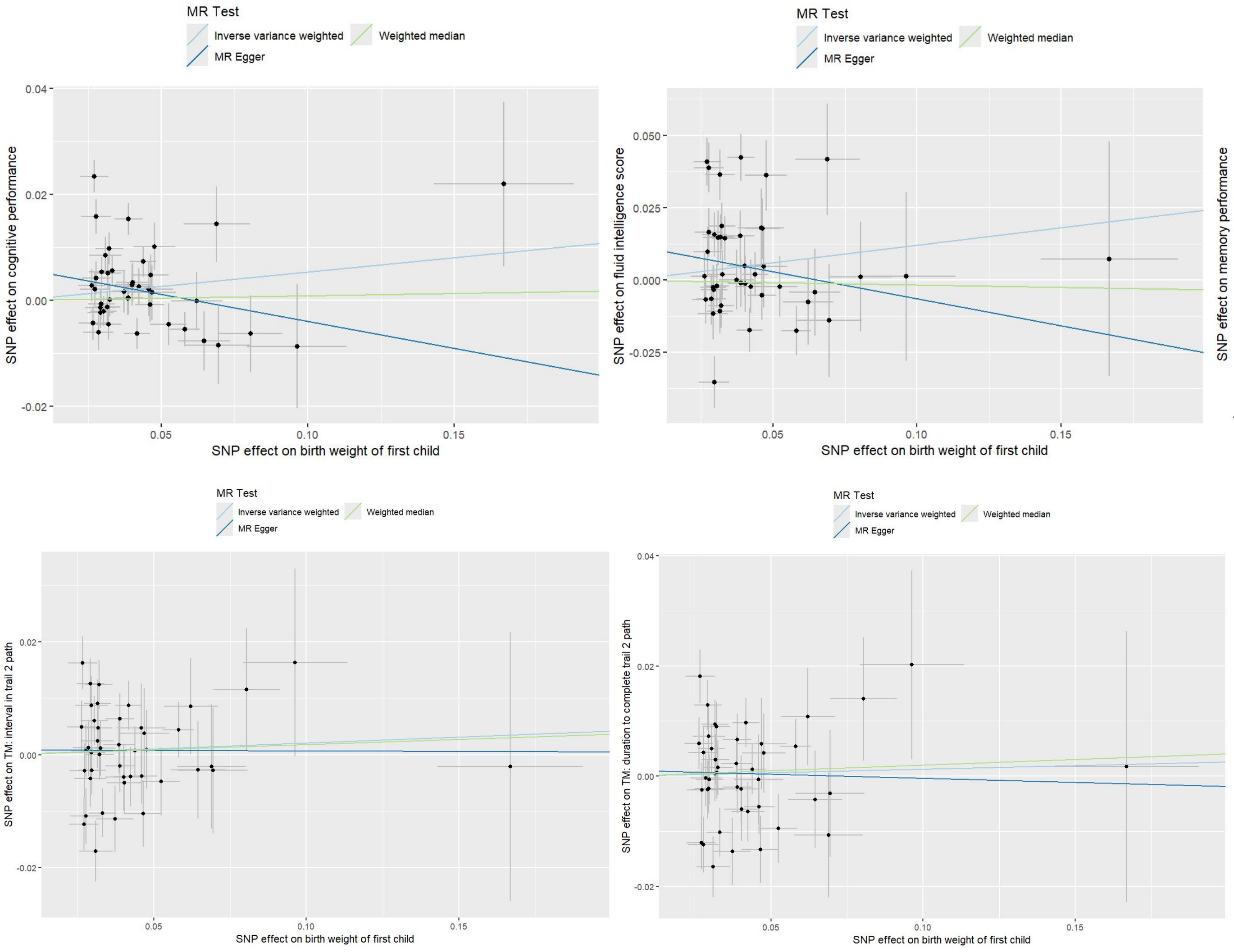

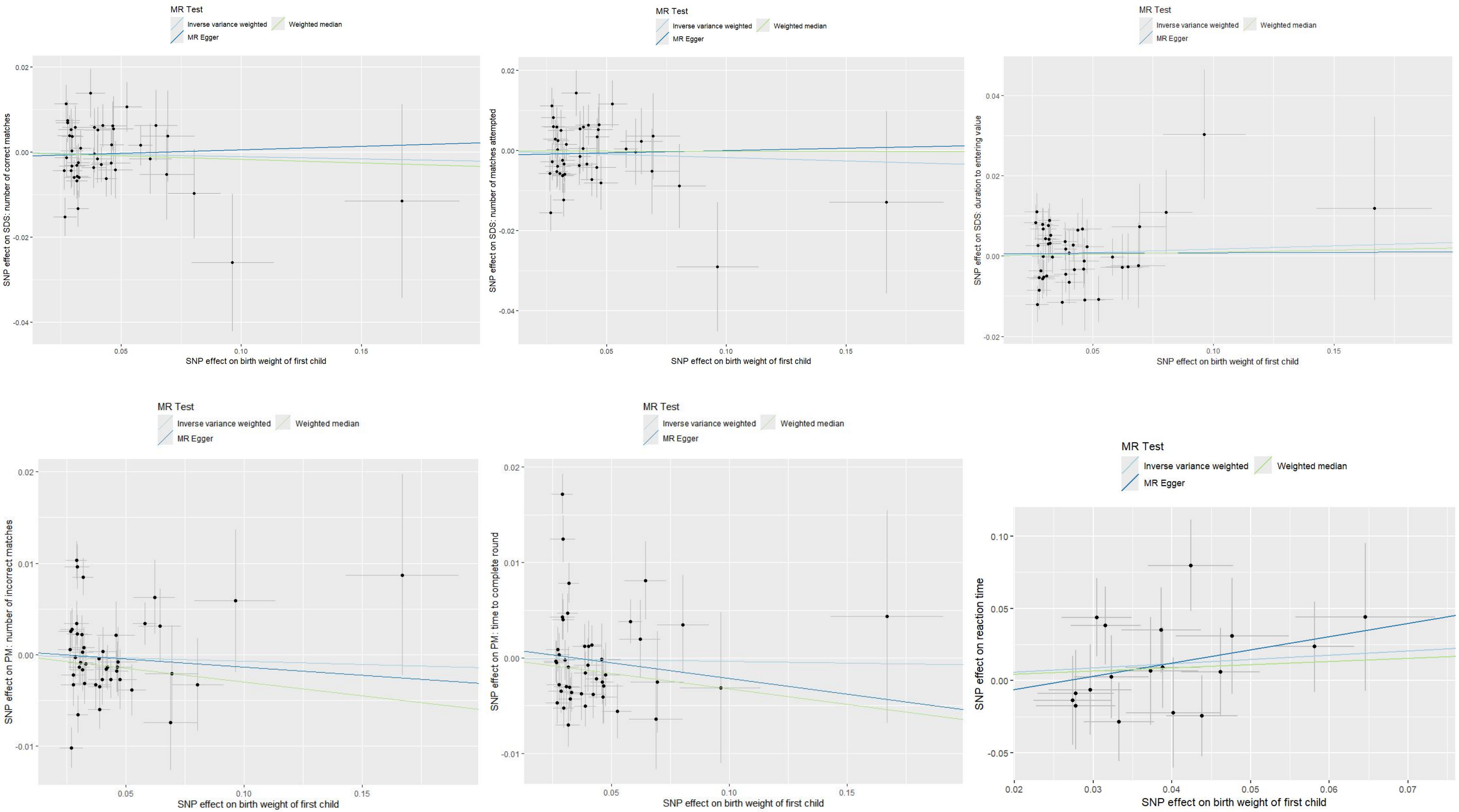
MR Test
Inverse variance weighted
MR Egger
Weighted median
SNP effect on SDS: number of correct matches
SNP effect on SDS: number of matches attempted
SNP effect on SDS: duration to entering value
SNP effect on PM: number of incorrect matches
SNP effect on PM: time to complete round
SNP effect on reaction time
SNP effect on birth weight of first child

Exposure: Number of live births

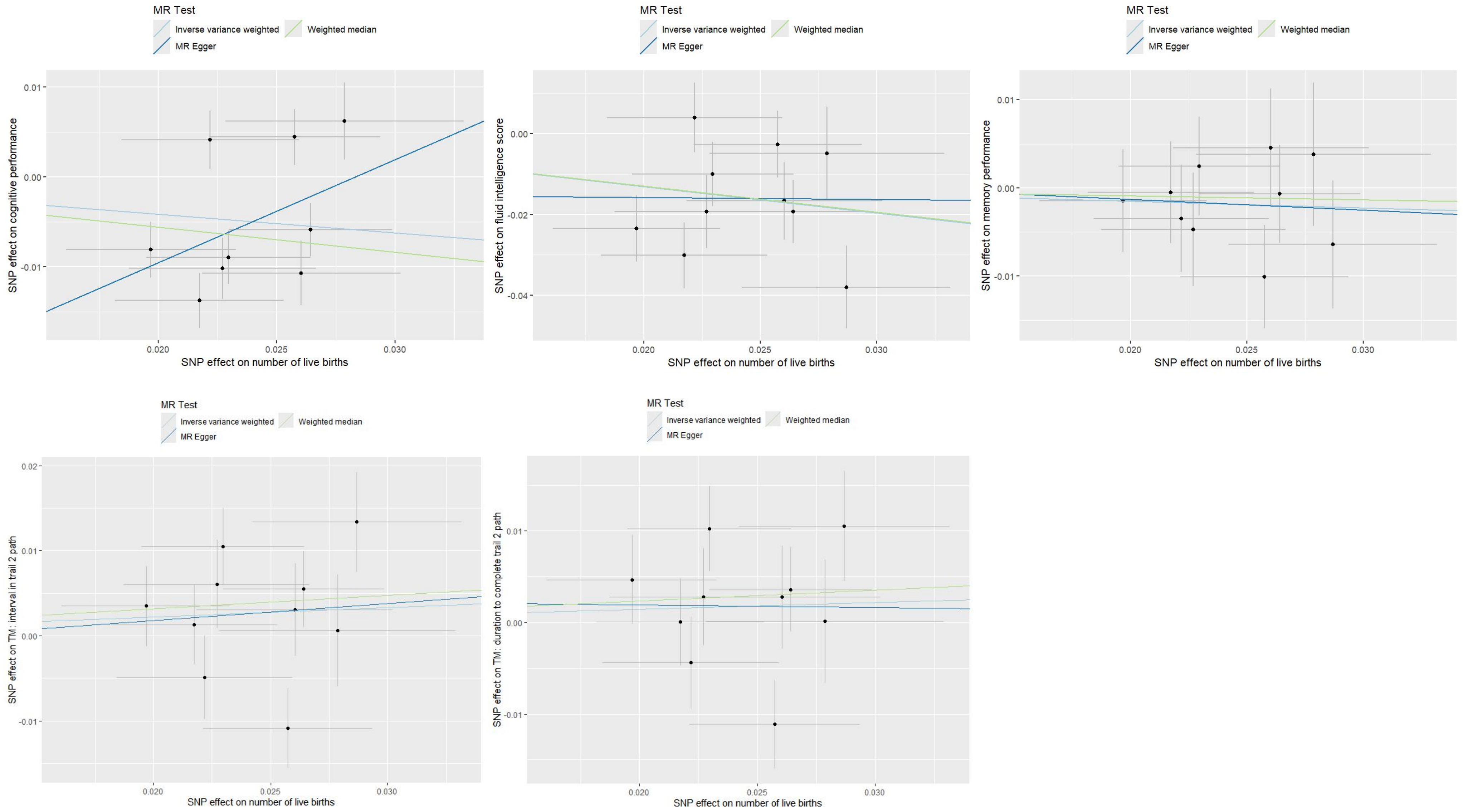

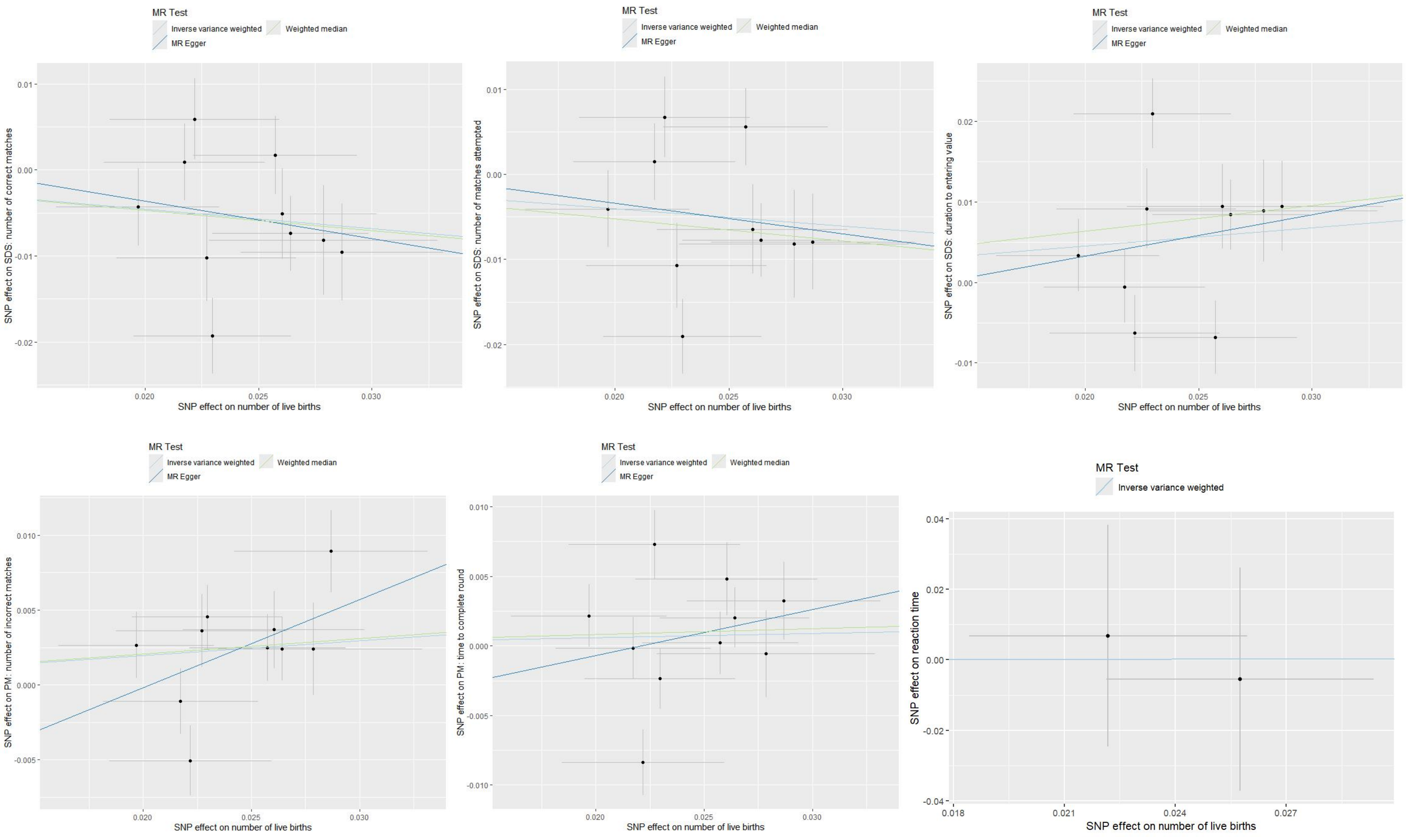

MR Test
Inverse variance weighted
Weighted median
MR Egger
SNP effect on SDS: number of correct matches
SNP effect on number of live births
SNP effect on SDS: number of matches attempted
SNP effect on SDS: duration to entering value
SNP effect on PM: number of incorrect matches
SNP effect on PM: time to complete round
SNP effect on reaction time
0.01
0.00
-0.01
-0.02
0.020
0.025
0.030
0.010
0.005
0.000
-0.005
-0.010
0.04
0.02
0.00
-0.02
-0.04
0.018
0.021
0.024
0.027

Exposure: Medical abortion

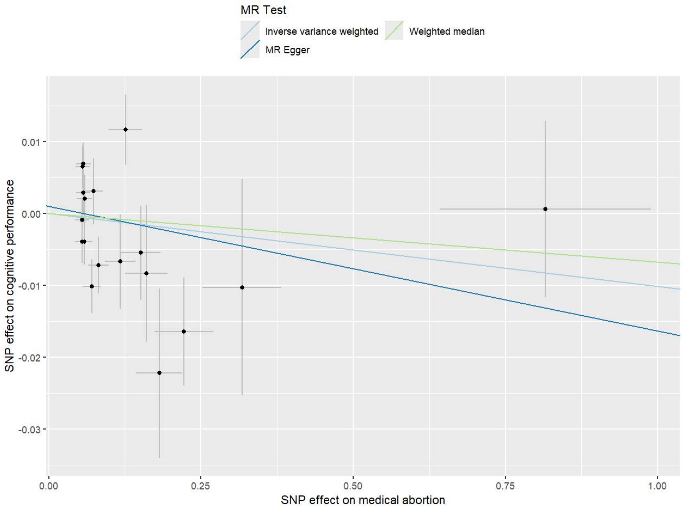

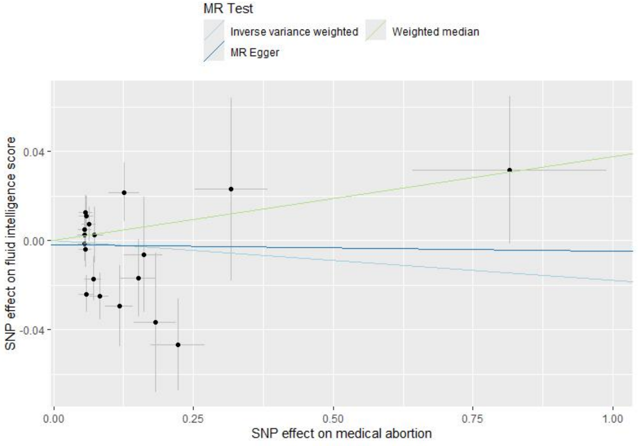

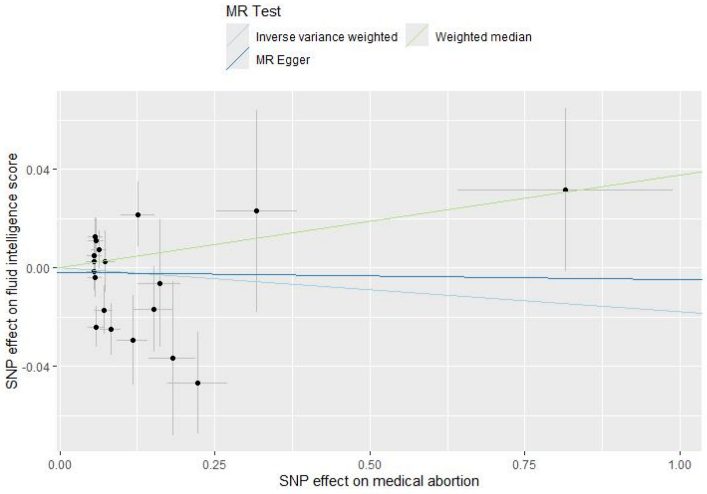

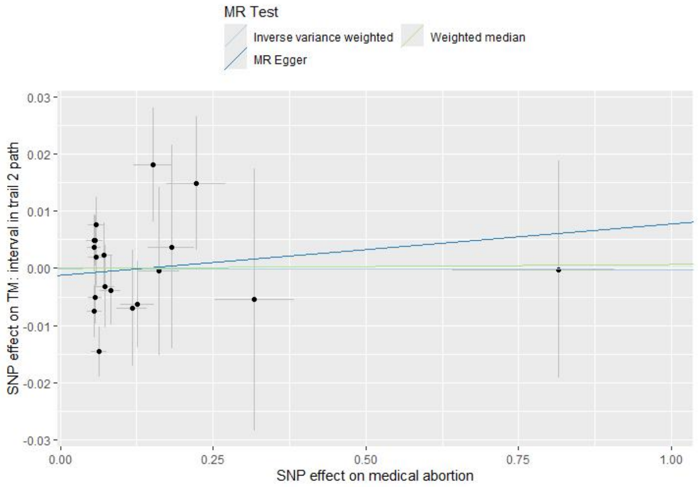

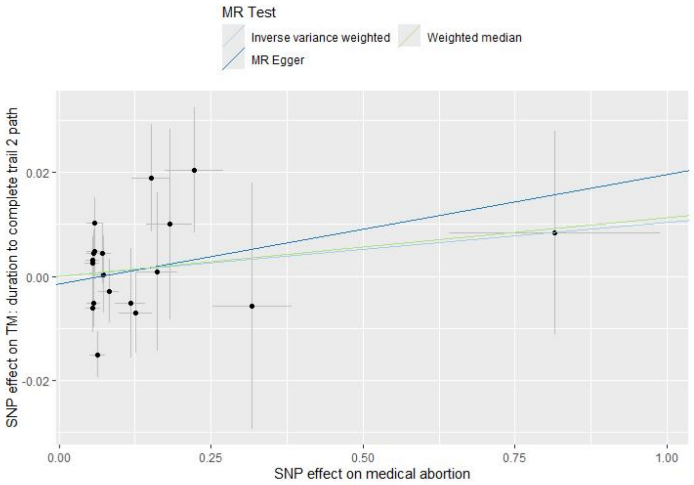

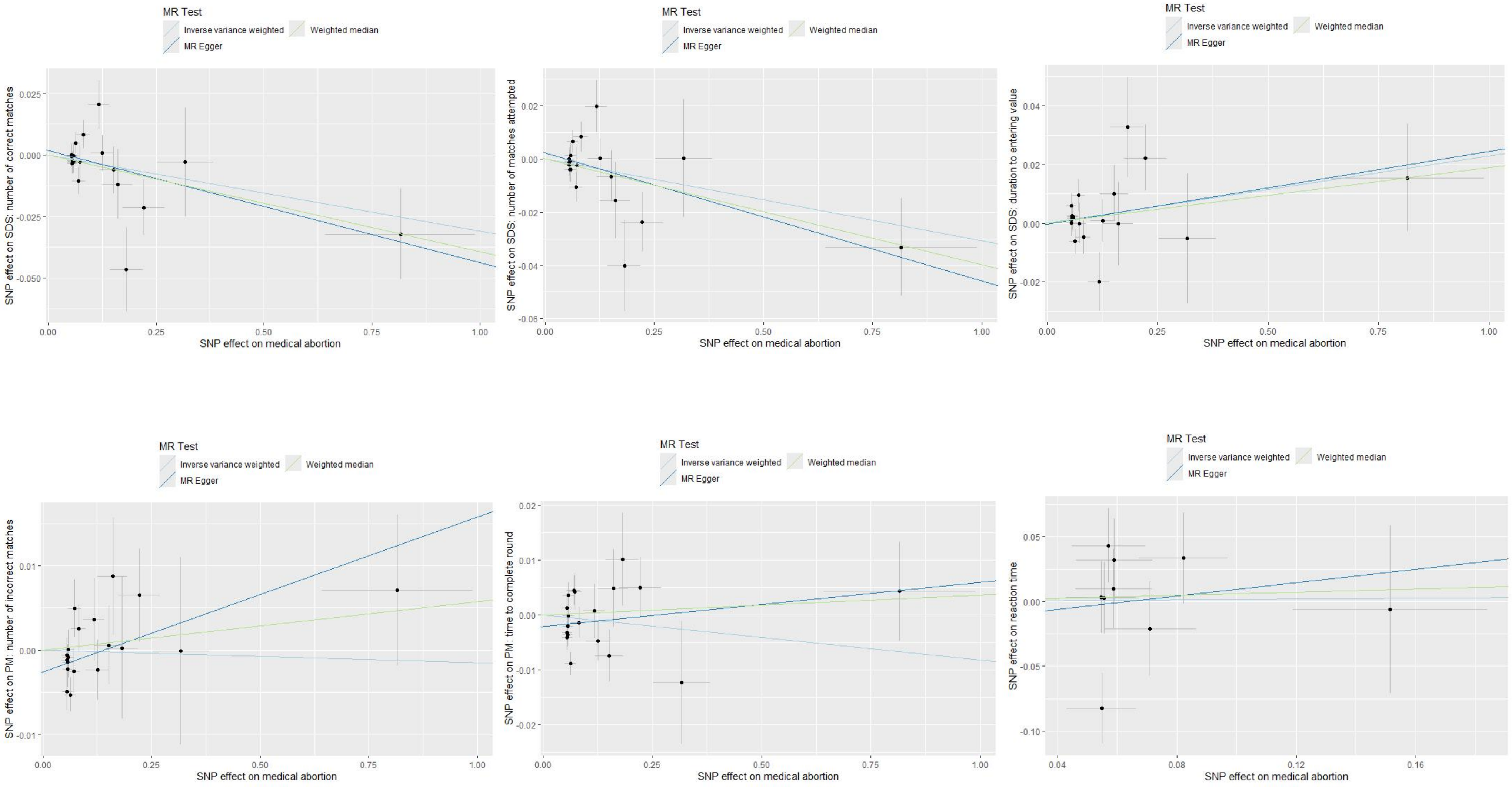
MR Test
Inverse variance weighted
Weighted median
MR Egger
SNP effect on SDS: number of correct matches
SNP effect on medical abortion
SNP effect on SDS: number of matches attempted
SNP effect on medical abortion
SNP effect on SDS: duration to entering value
SNP effect on medical abortion
SNP effect on PM: number of incorrect matches
SNP effect on medical abortion
SNP effect on PM: time to complete round
SNP effect on medical abortion
SNP effect on reaction time
SNP effect on medical abortion

Exposure: Ever taken oral contraceptive pill

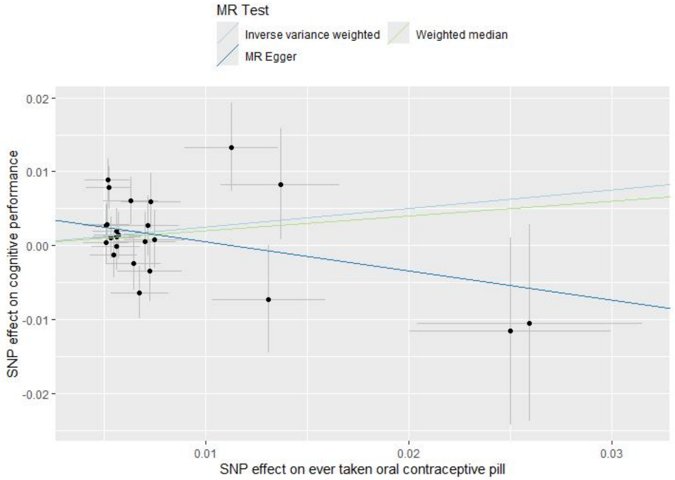

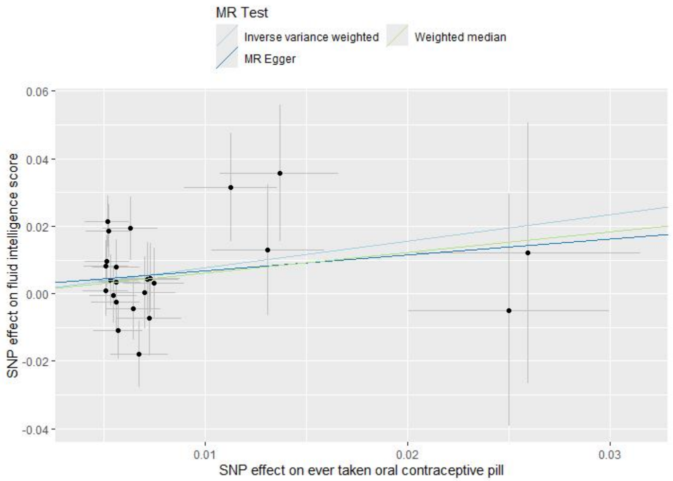

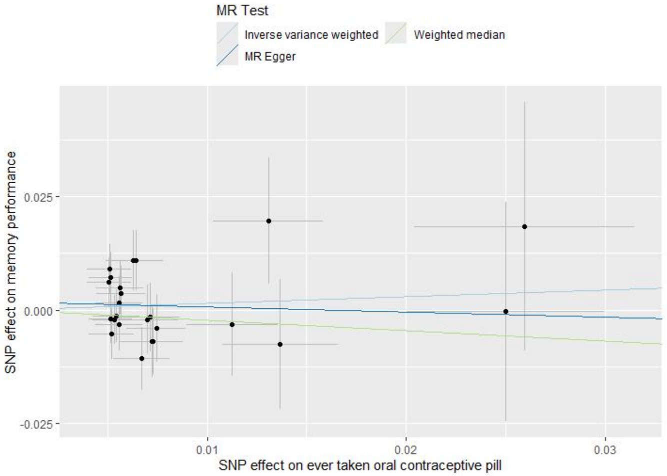

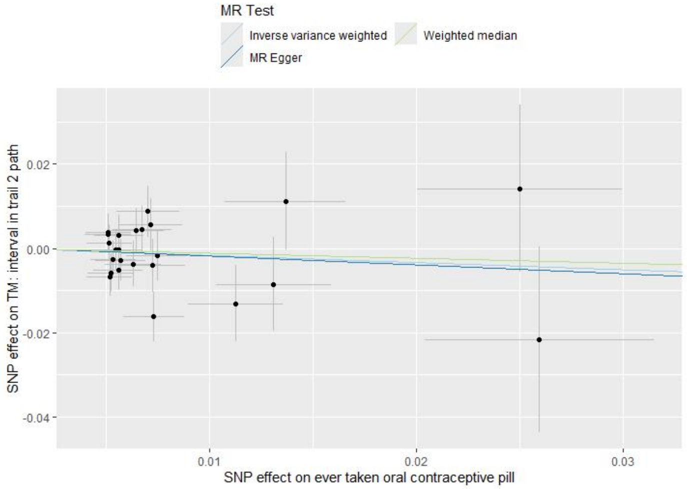

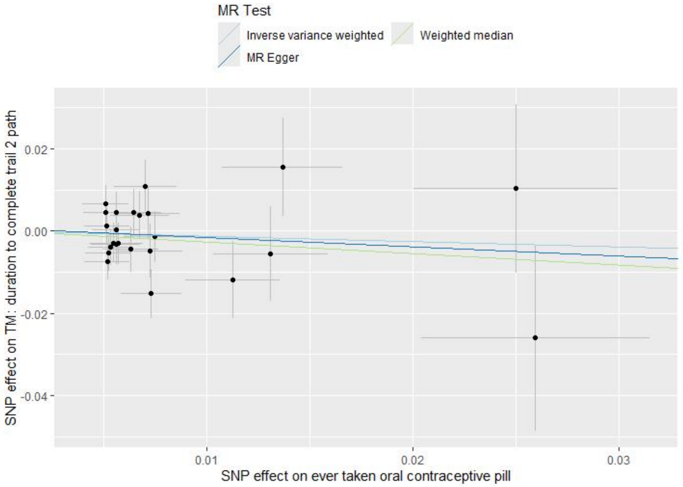

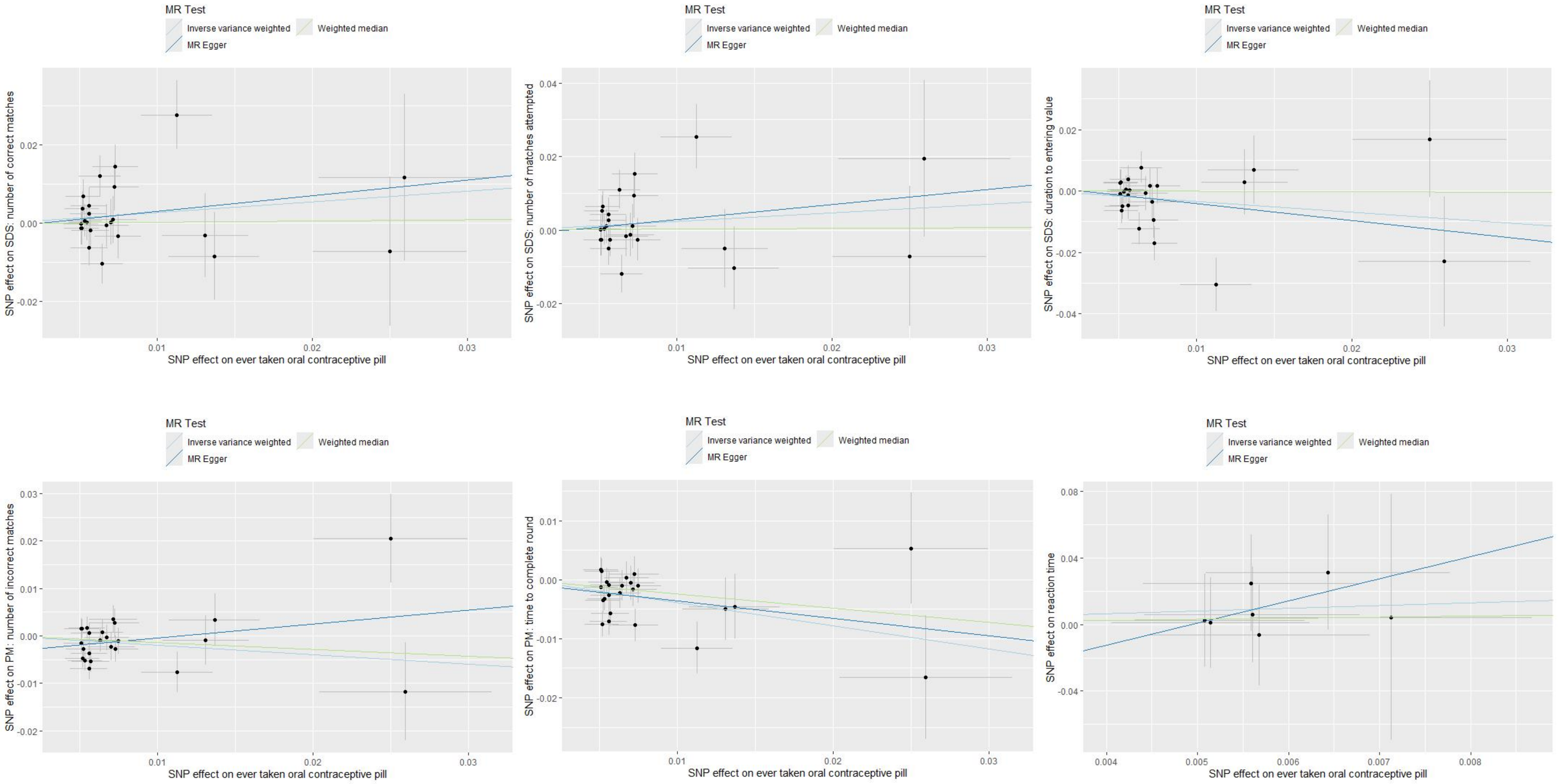
MR Test
Inverse variance weighted
Weighted median
MR Egger
SNP effect on SDS: number of correct matches
SNP effect on SDS: number of matches attempted
SNP effect on SDS: duration to entering value
SNP effect on PM: number of incorrect matches
SNP effect on PM: time to complete round
SNP effect on reaction time
SNP effect on ever taken oral contraceptive pill

Exposure: Ever used hormone-replacement therapy

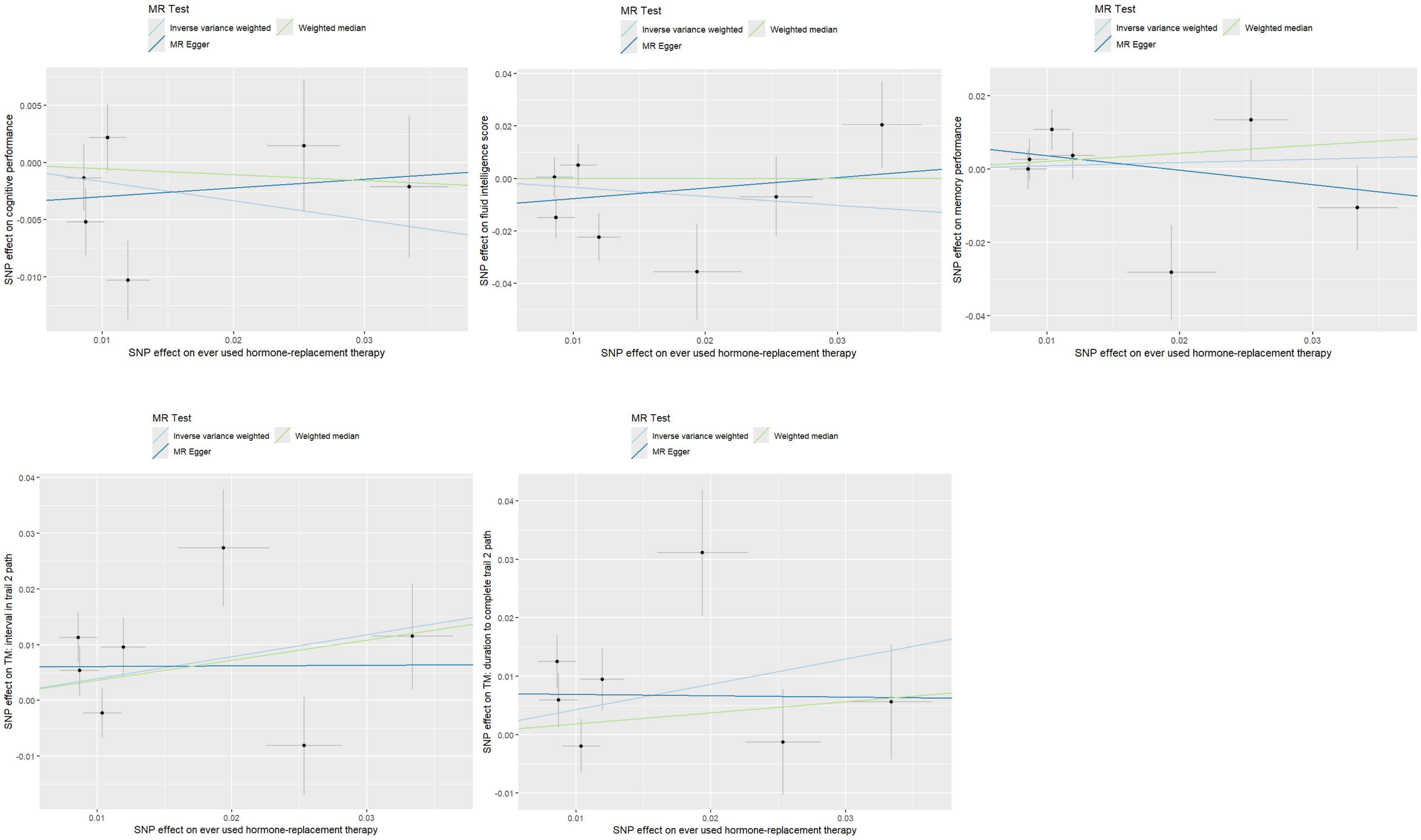

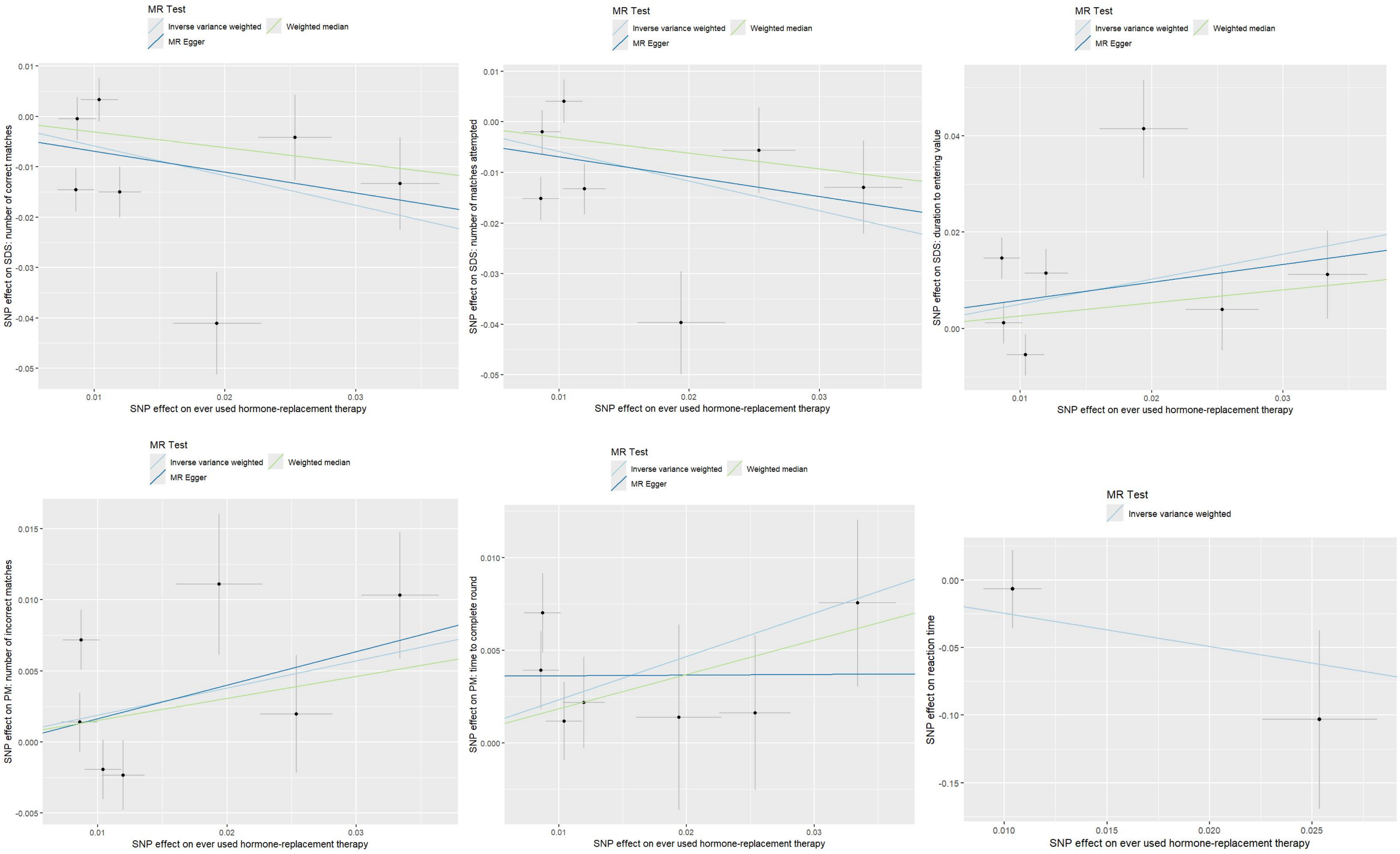
MR Test
Inverse variance weighted
Weighted median
MR Egger
SNP effect on SDS: number of correct matches
SNP effect on ever used hormone-replacement therapy
SNP effect on SDS: number of matches attempted
SNP effect on SDS: duration to entering value
SNP effect on PM: number of incorrect matches
SNP effect on PM: time to complete round
SNP effect on reaction time

Figure S3. Funnel plot for cogitive function regarding reproductive traits.

Exposure: Age at menarche

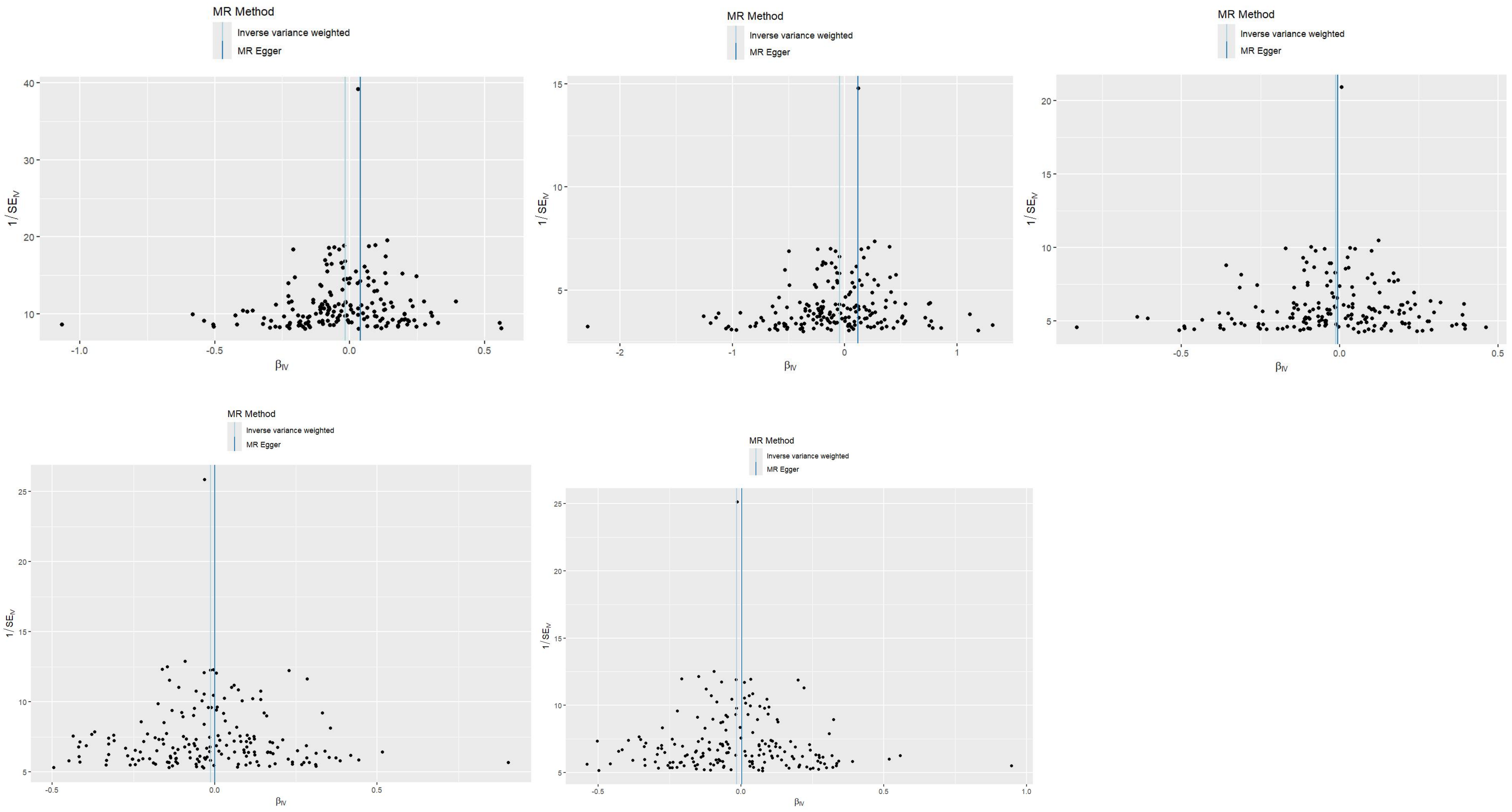

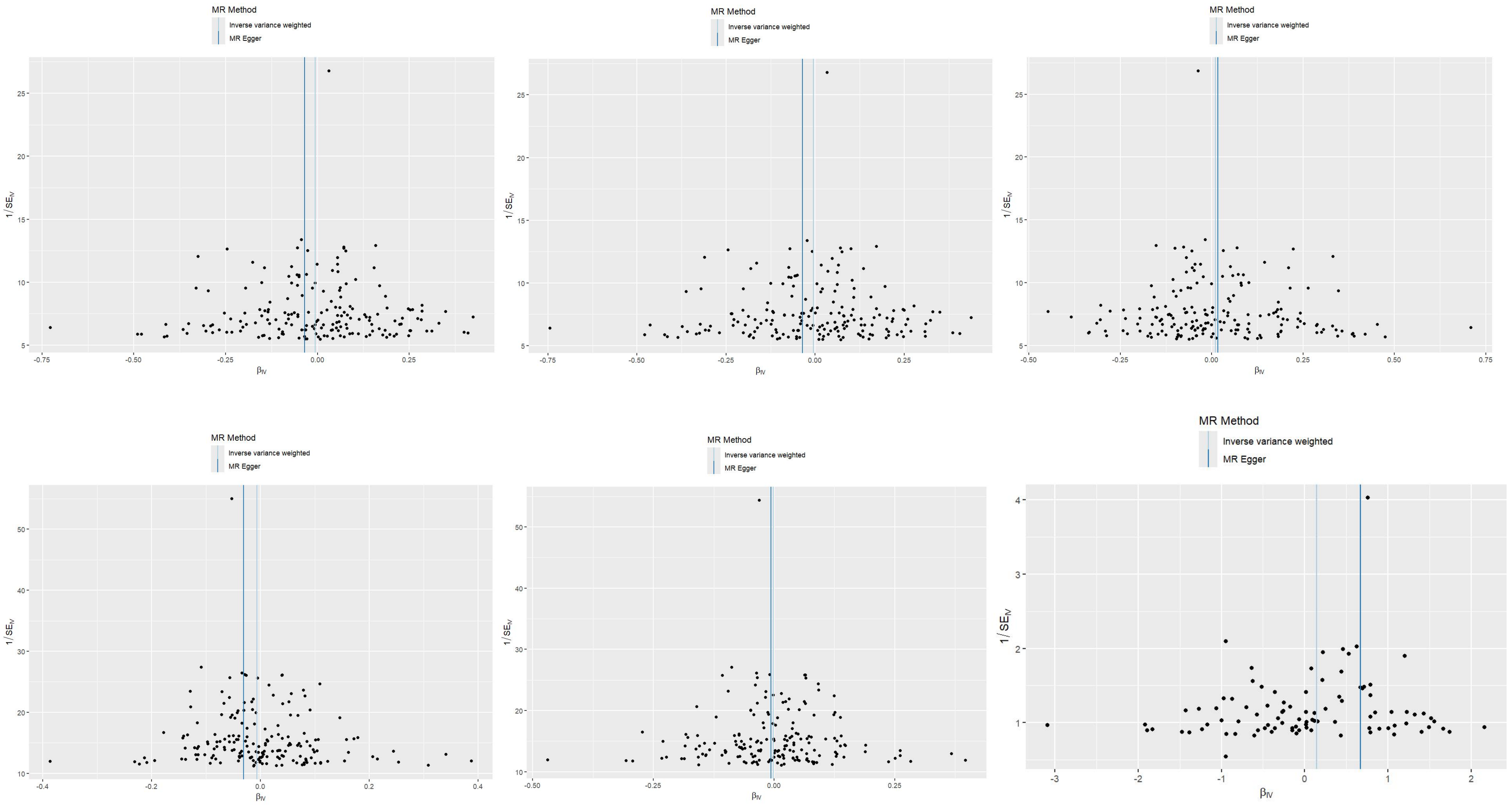

MR Method
Inverse variance weighted
MR Egger
$1/SE_{IV}$
β_{IV}

Exposure: Age at menopause

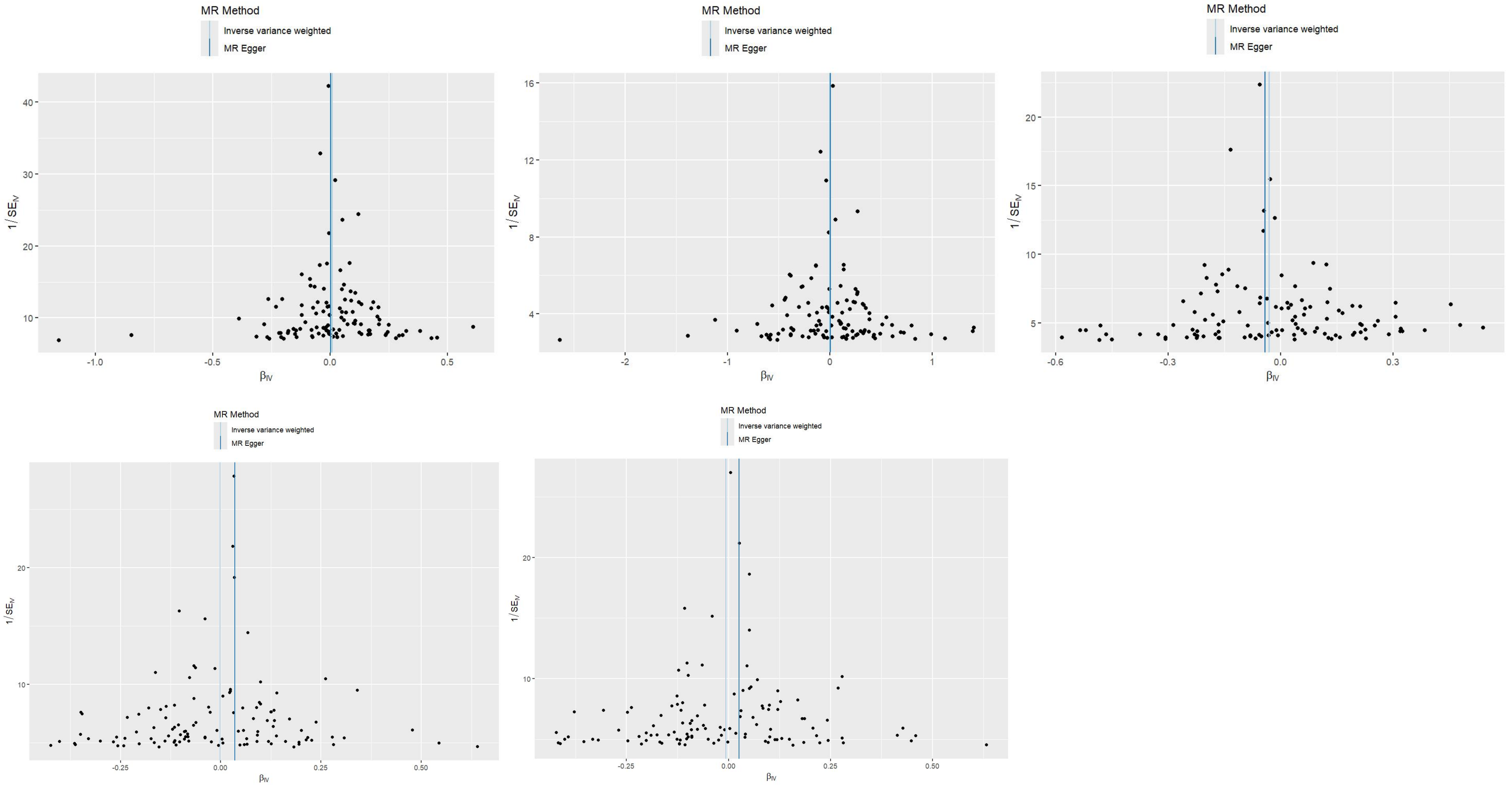

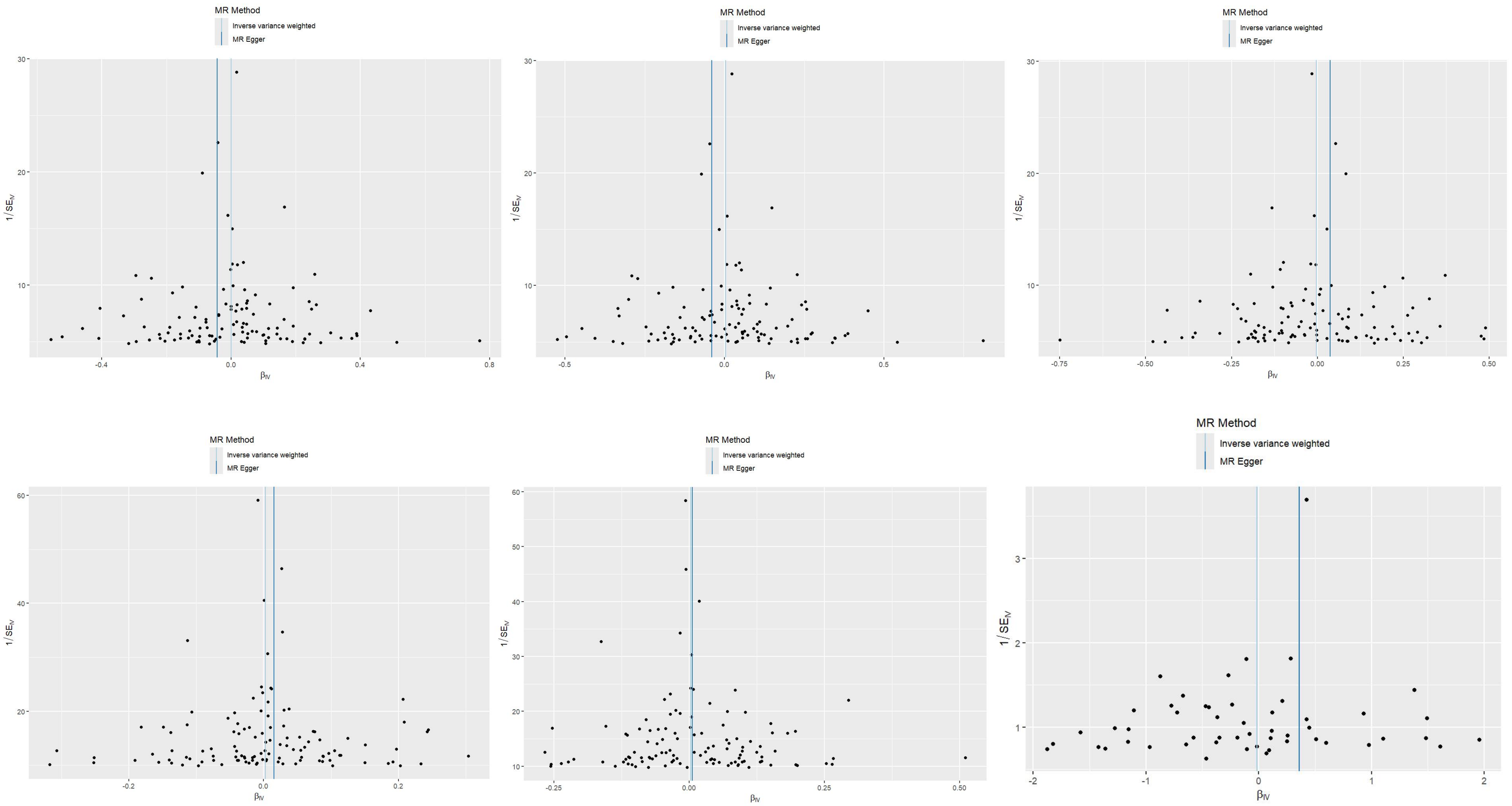

MR Method
Inverse variance weighted
MR Egger
1/SE_IV
β_IV

Exposure: Age at first sexual intercourse

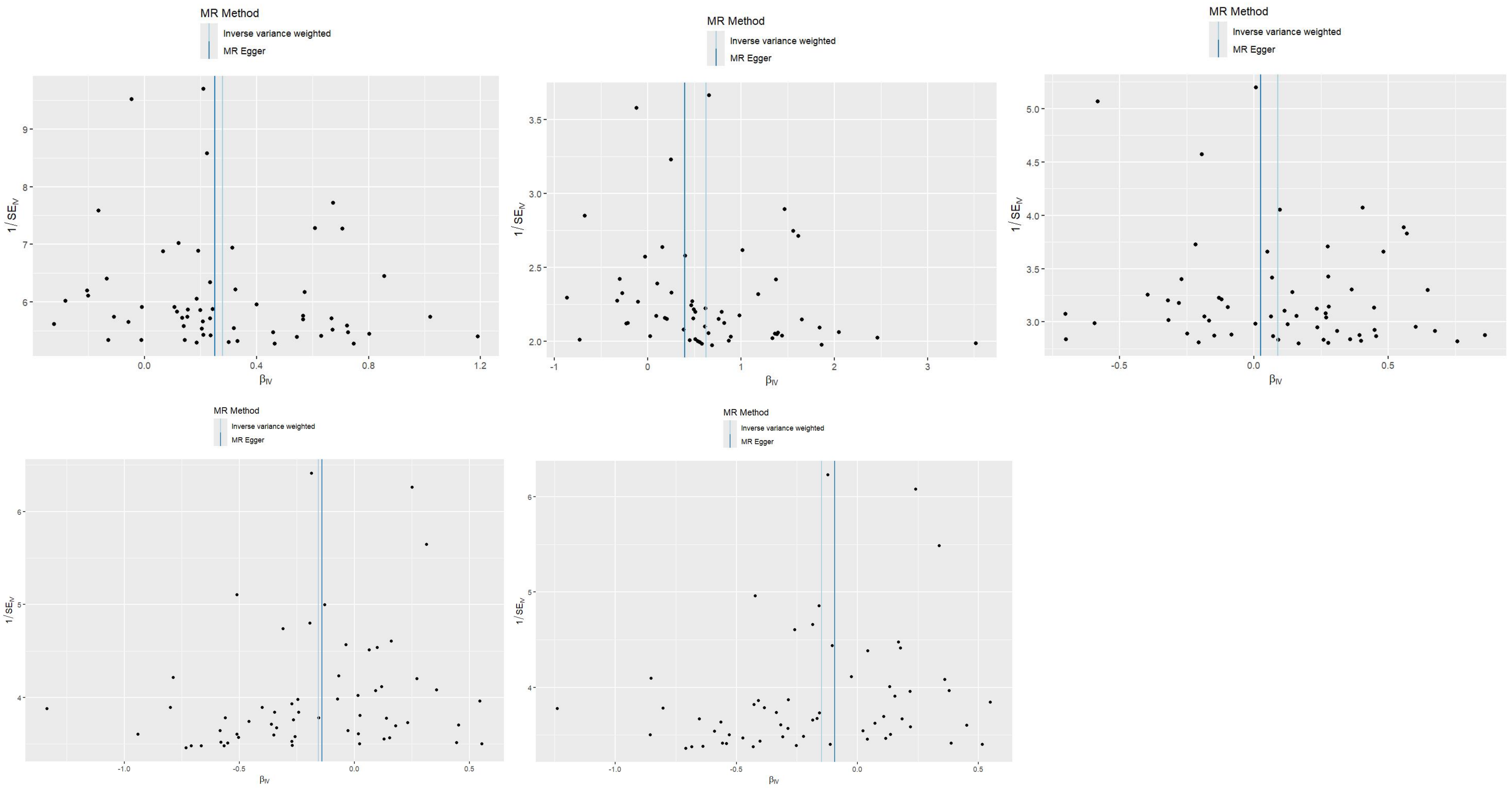

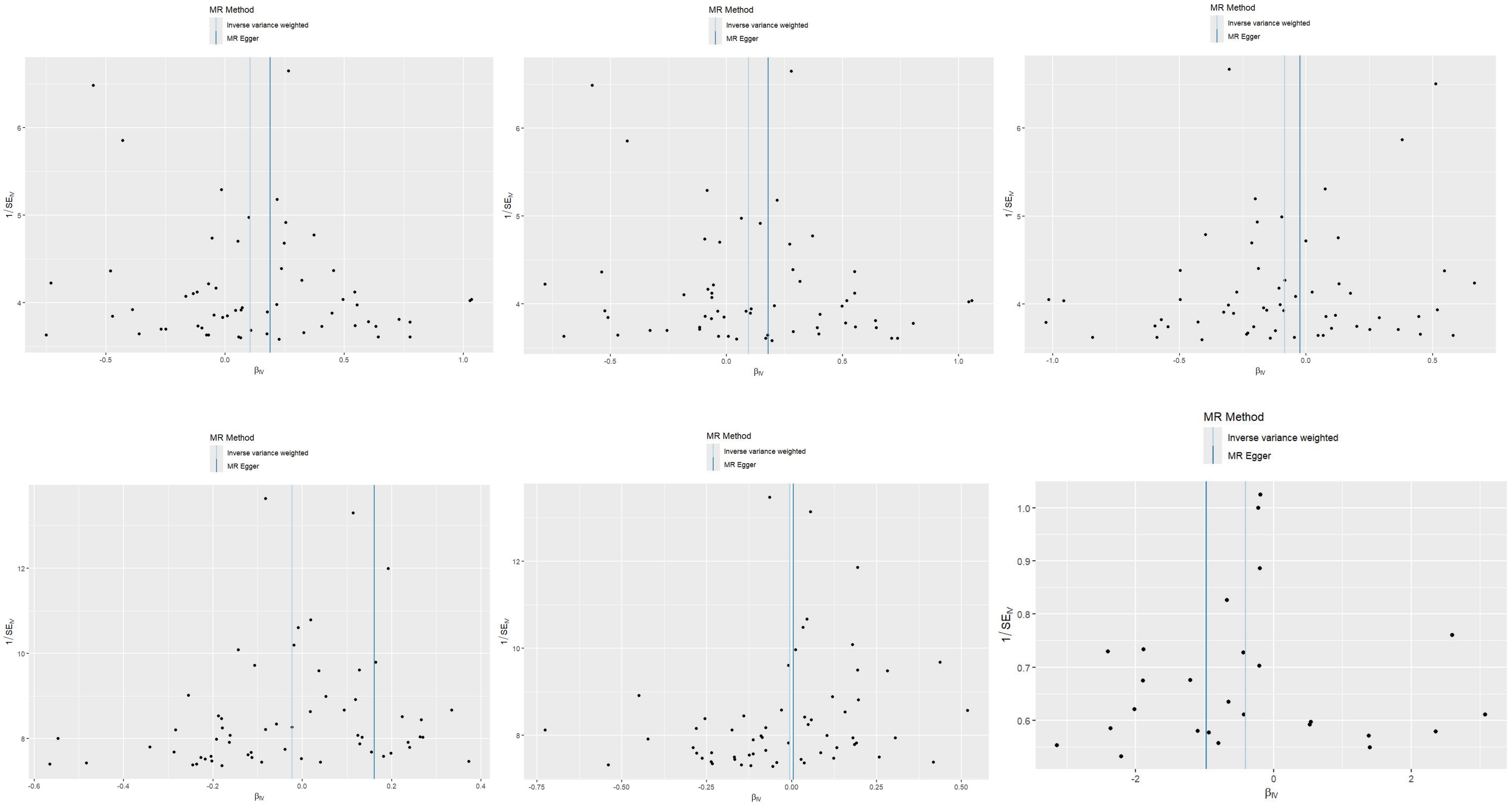
MR Method
Inverse variance weighted
MR Egger
$1/SE_{IV}$
β_{IV}

Exposure: Age at first birth

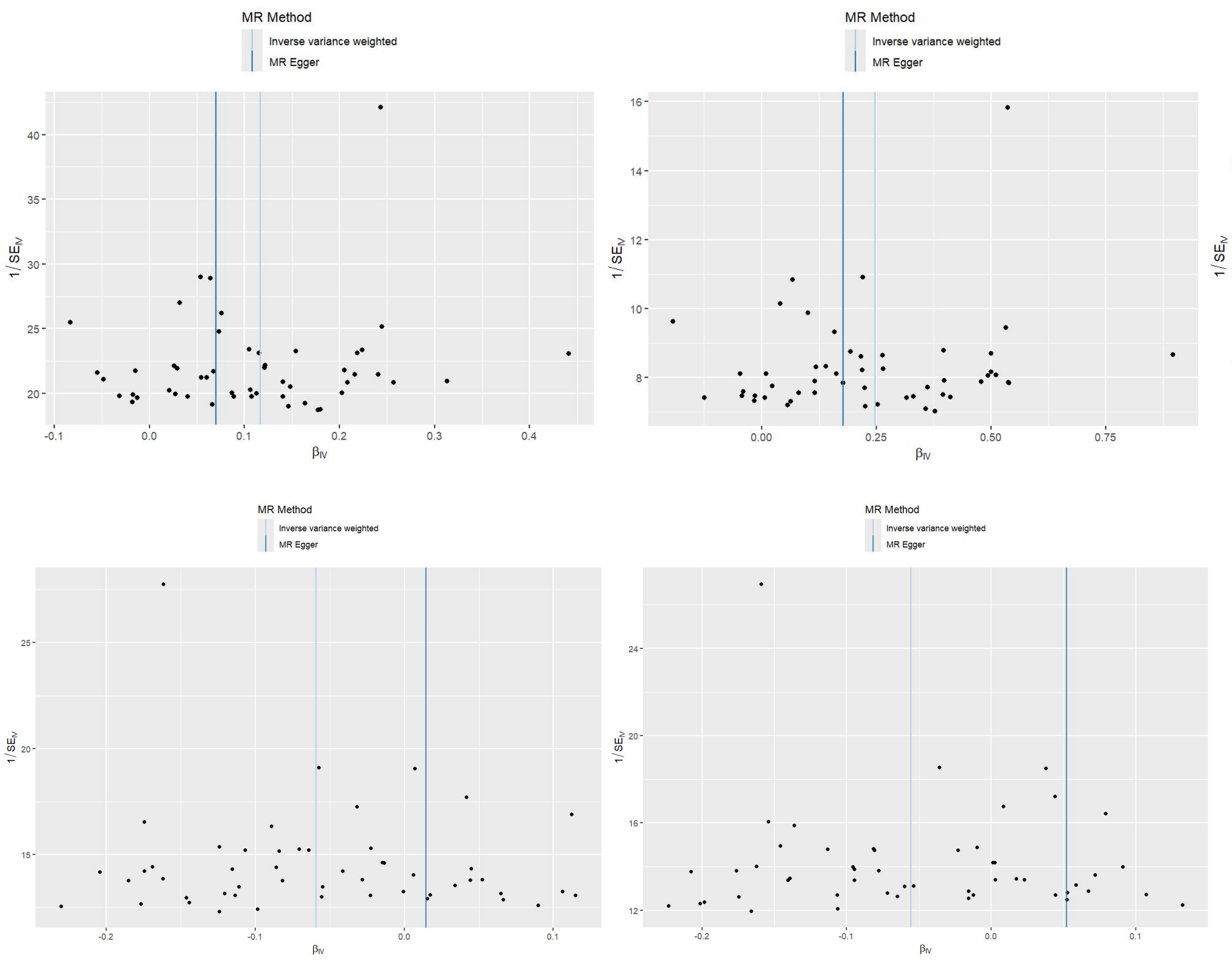

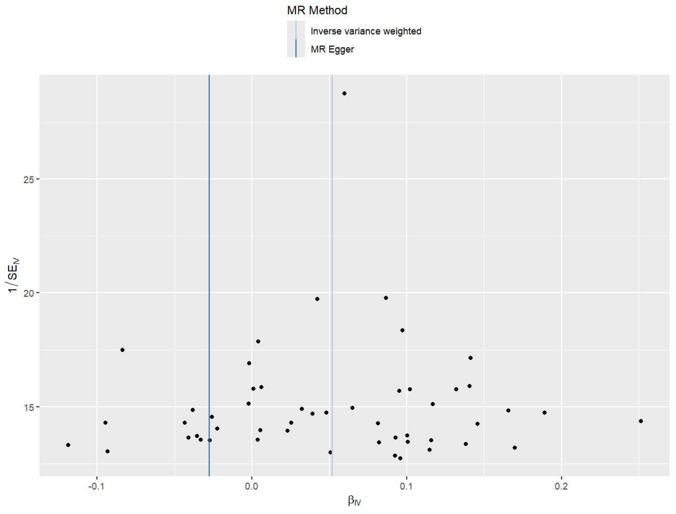
MR Method
Inverse variance weighted
MR Egger
$1/SE_{IV}$
β_{IV}
15
20
25
-0.1
0.0
0.1
0.2

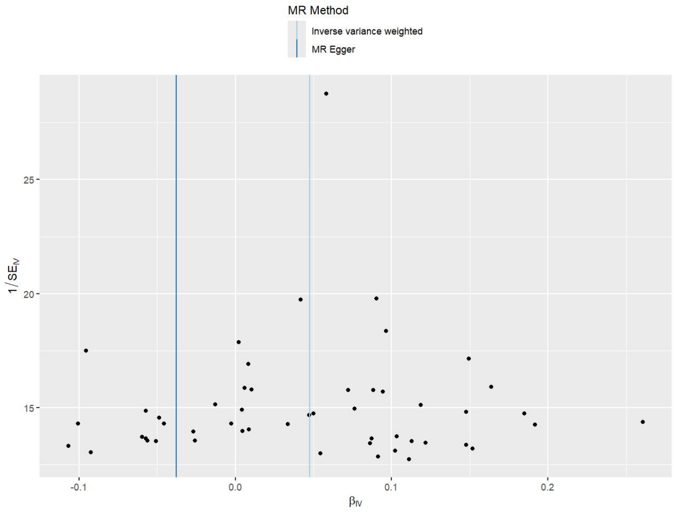
MR Method
Inverse variance weighted
MR Egger
$1/SE_{IV}$
β_{IV}
15
20
25
-0.1
0.0
0.1
0.2

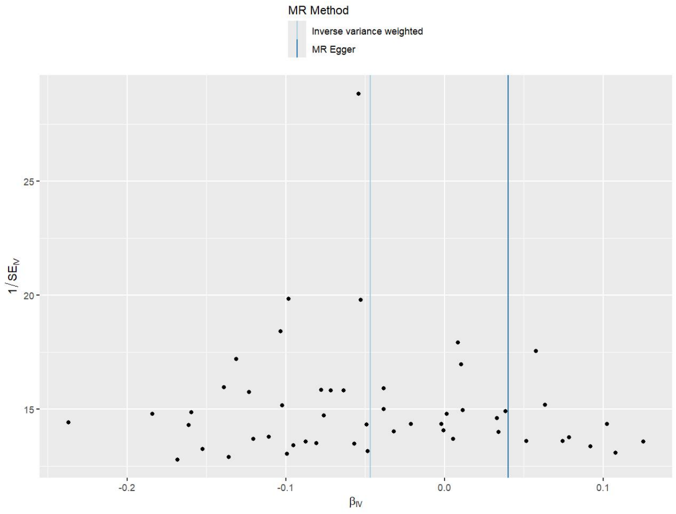
MR Method
Inverse variance weighted
MR Egger
$1/SE_{IV}$
β_{IV}
15
20
25
-0.2
-0.1
0.0
0.1

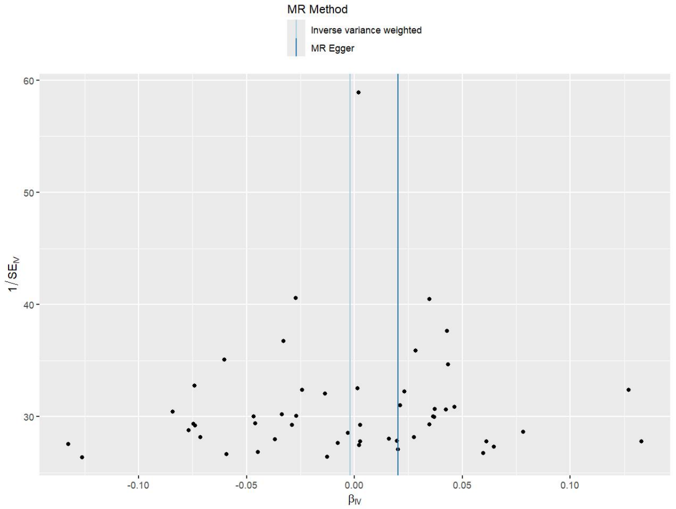
MR Method
Inverse variance weighted
MR Egger
$1/SE_{IV}$
β_{IV}
30
40
50
60
-0.10
-0.05
0.00
0.05
0.10

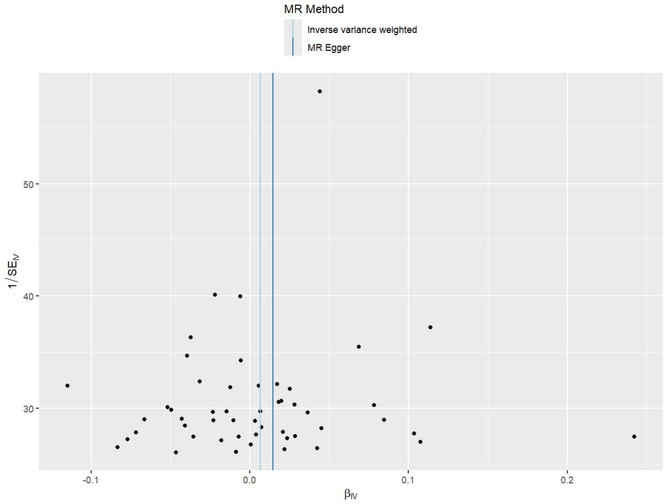
MR Method
Inverse variance weighted
MR Egger
$1/SE_{IV}$
β_{IV}
30
40
50
-0.1
0.0
0.1
0.2

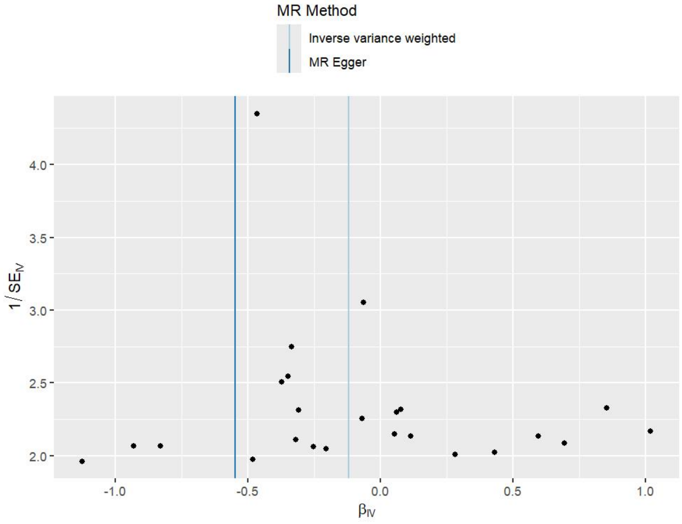
MR Method
Inverse variance weighted
MR Egger
$1/SE_{IV}$
β_{IV}
2.0
2.5
3.0
3.5
4.0
-1.0
-0.5
0.0
0.5
1.0

Exposure: Birth weight of first child

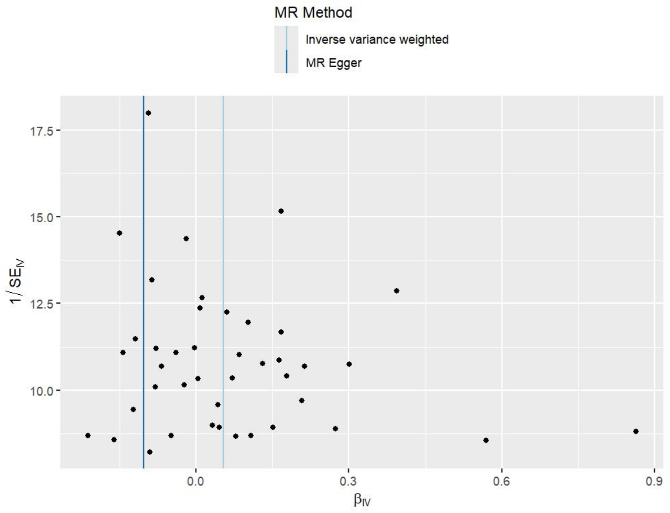

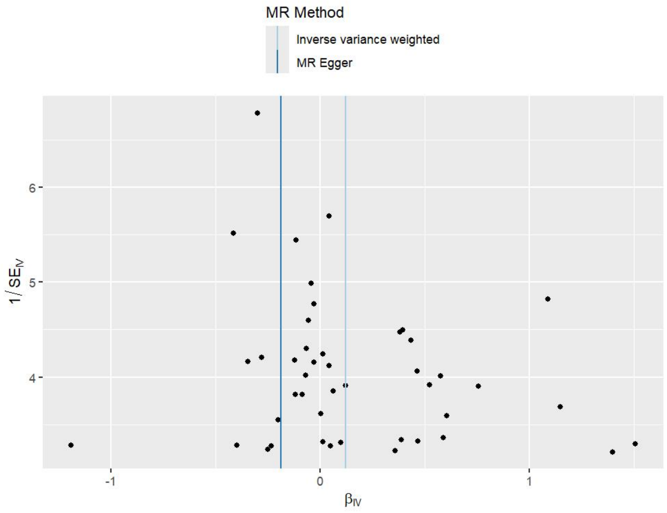

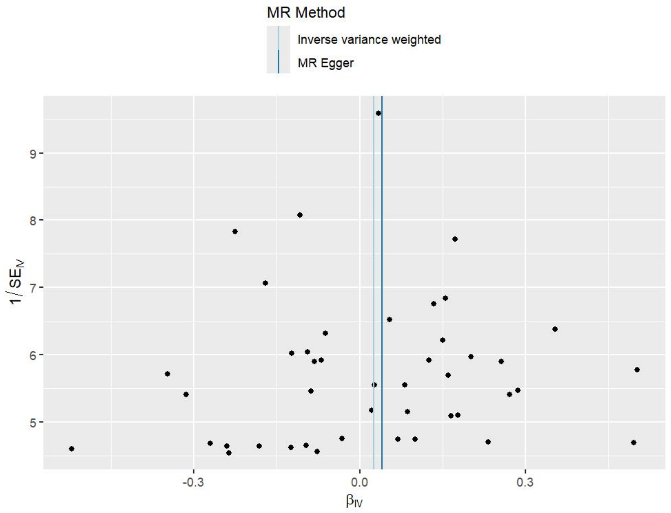

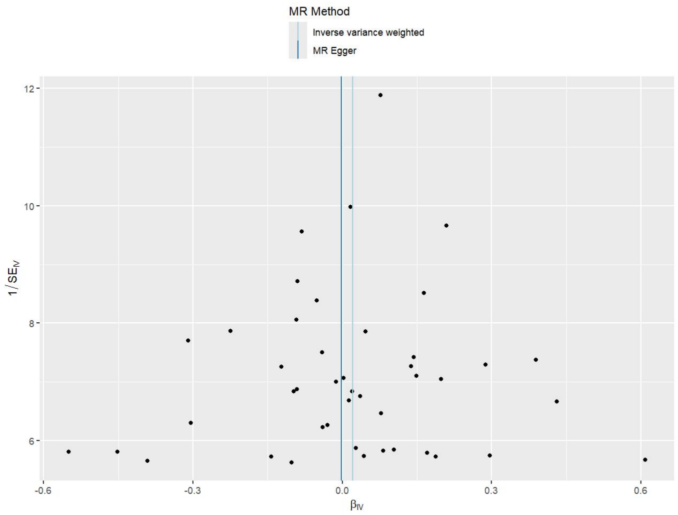

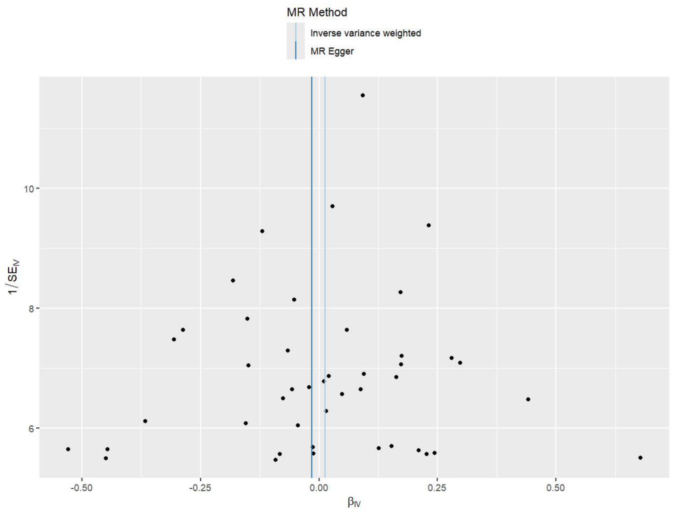

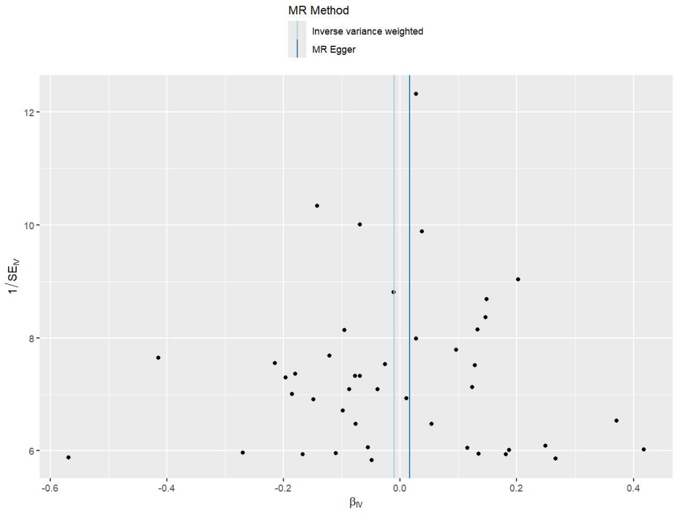
MR Method
Inverse variance weighted
MR Egger
$1/SE_{IV}$
β_{IV}

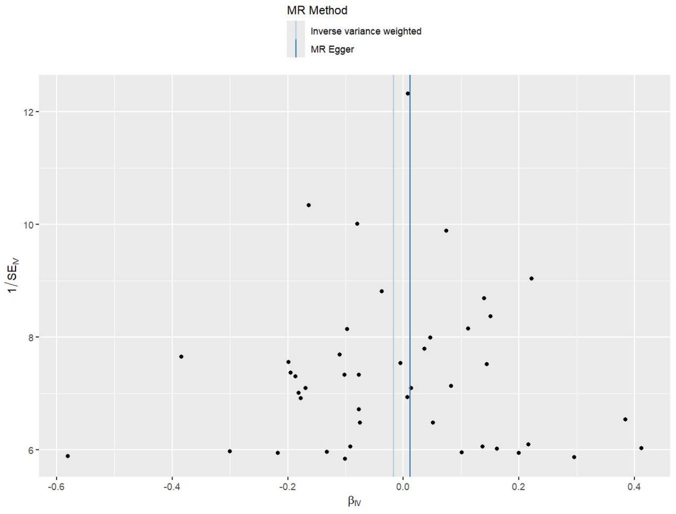
MR Method
Inverse variance weighted
MR Egger
$1/SE_{IV}$
β_{IV}

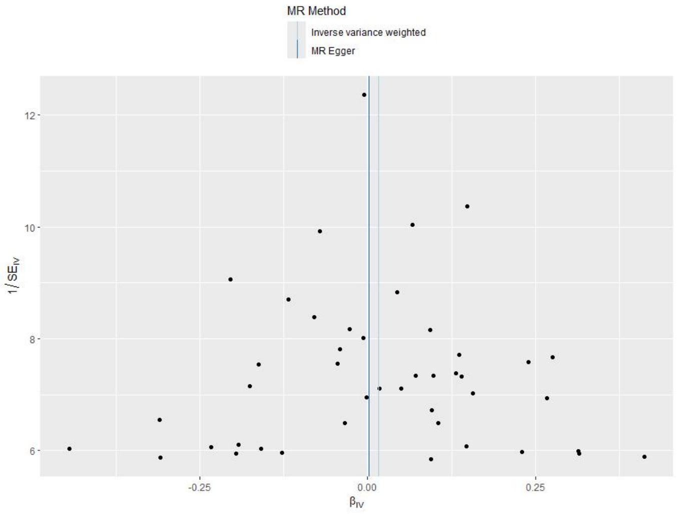
MR Method
Inverse variance weighted
MR Egger
$1/SE_{IV}$
β_{IV}

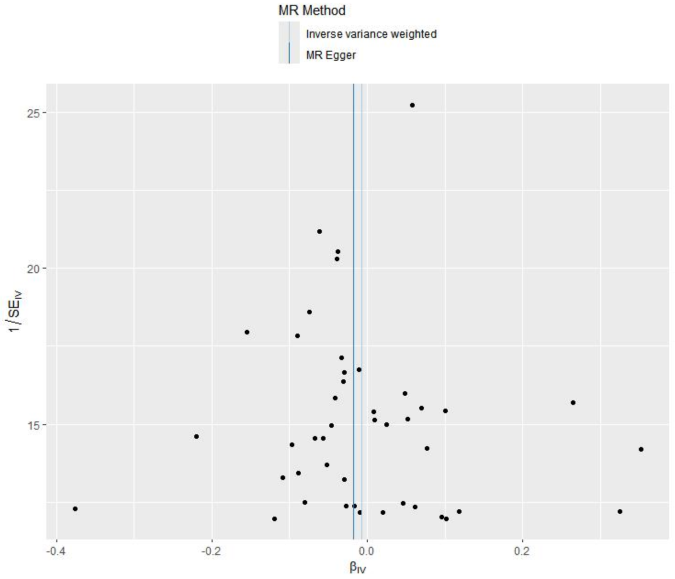
MR Method
Inverse variance weighted
MR Egger
$1/SE_{IV}$
β_{IV}

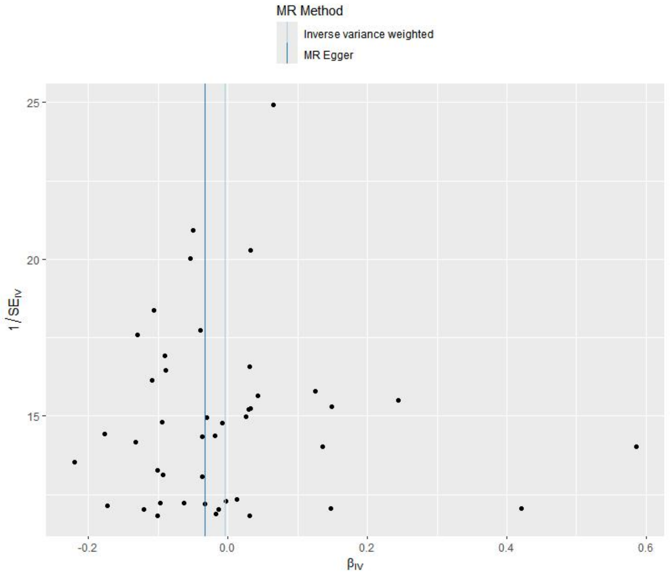
MR Method
Inverse variance weighted
MR Egger
$1/SE_{IV}$
β_{IV}

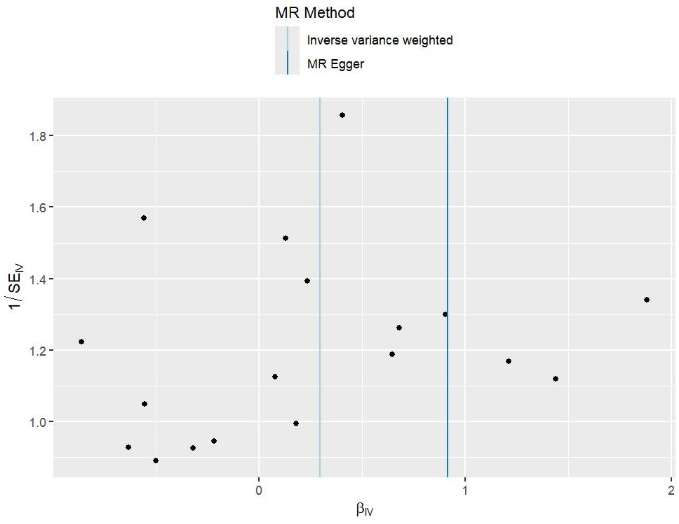
MR Method
Inverse variance weighted
MR Egger
$1/SE_{IV}$
β_{IV}

Exposure: Number of live births

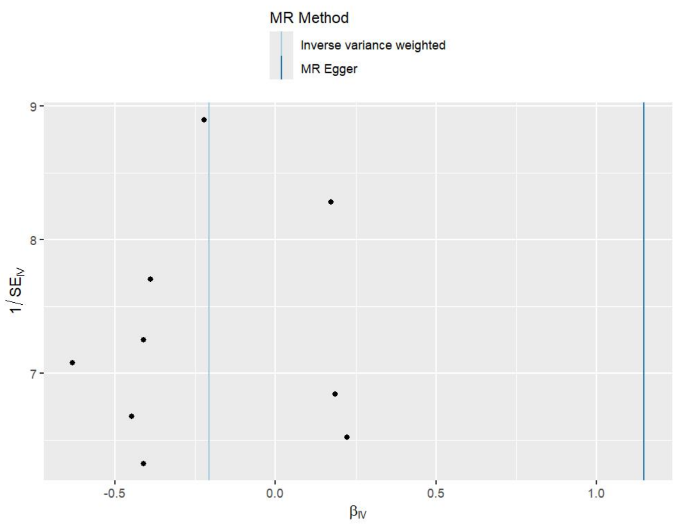

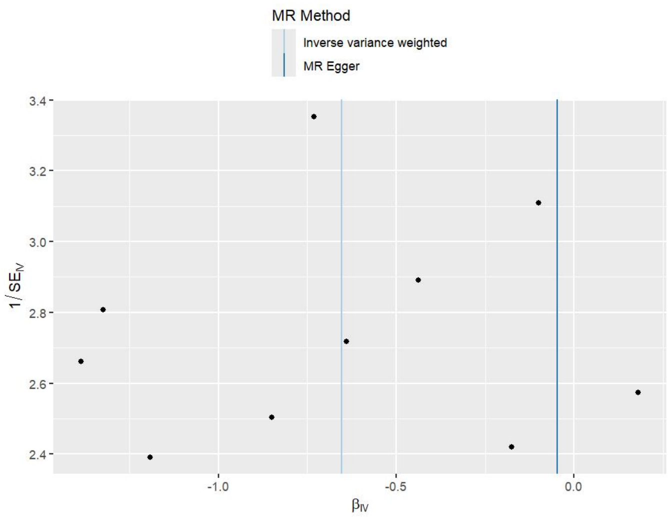

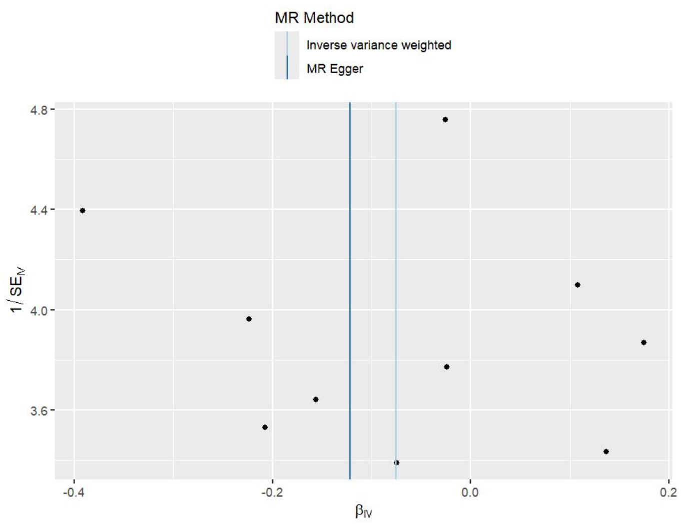

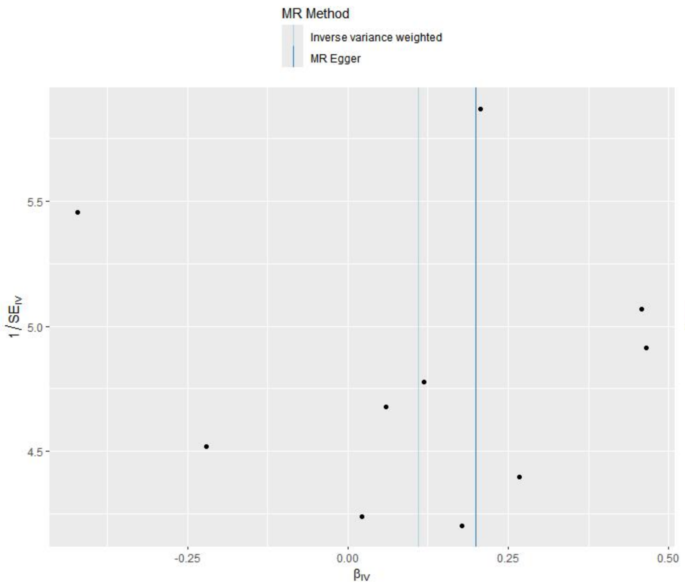

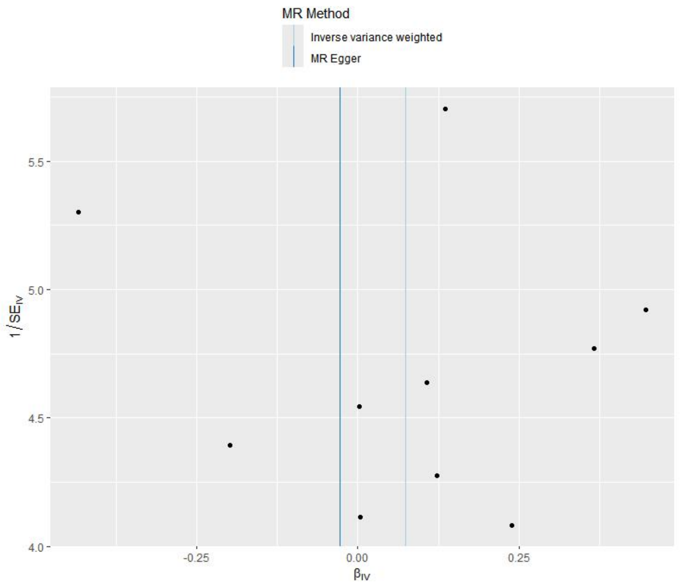

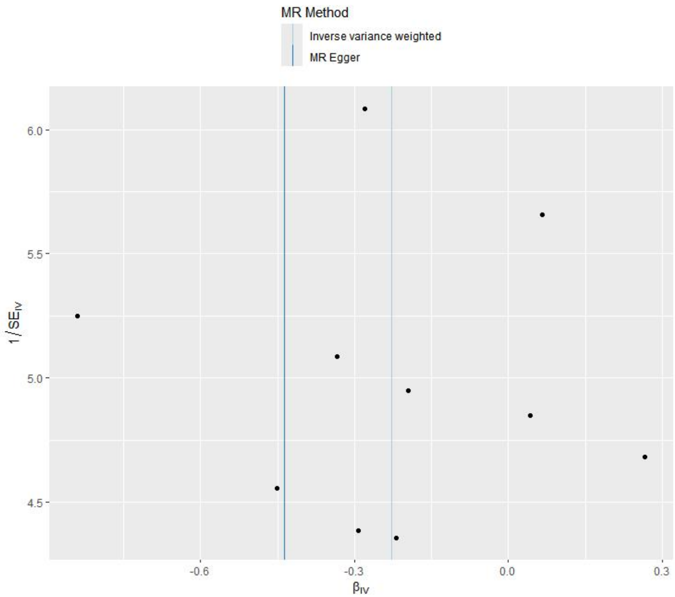
MR Method
Inverse variance weighted
MR Egger
1/SE$_{IV}$
6.0
5.5
5.0
4.5
-0.6
-0.3
0.0
0.3
β$_{IV}$

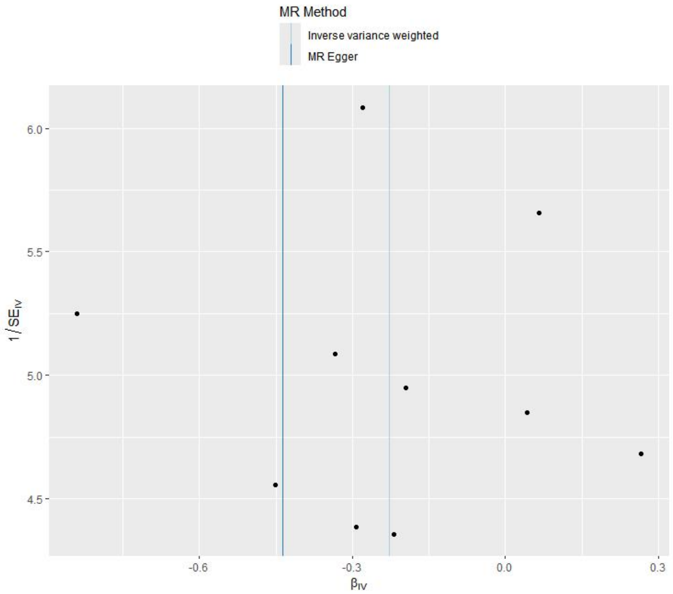
MR Method
Inverse variance weighted
MR Egger
1/SE$_{IV}$
6.0
5.5
5.0
4.5
-0.6
-0.3
0.0
0.3
β$_{IV}$

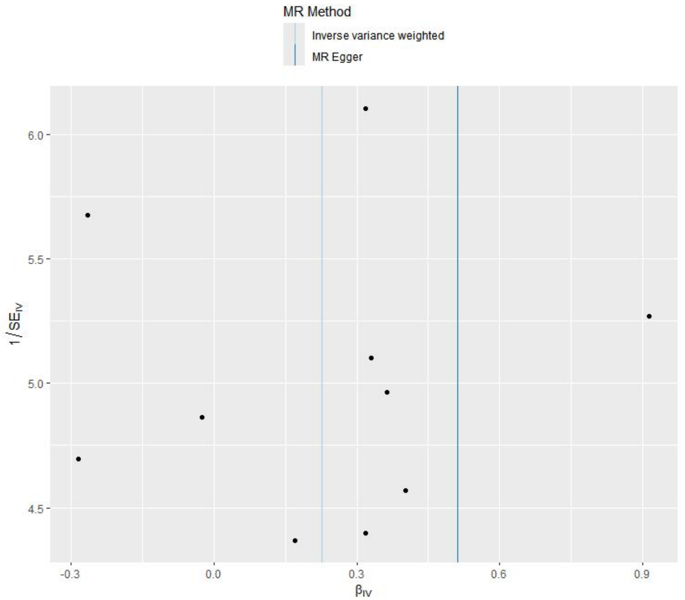
MR Method
Inverse variance weighted
MR Egger
1/SE$_{IV}$
6.0
5.5
5.0
4.5
-0.3
0.0
0.3
0.6
0.9
β$_{IV}$

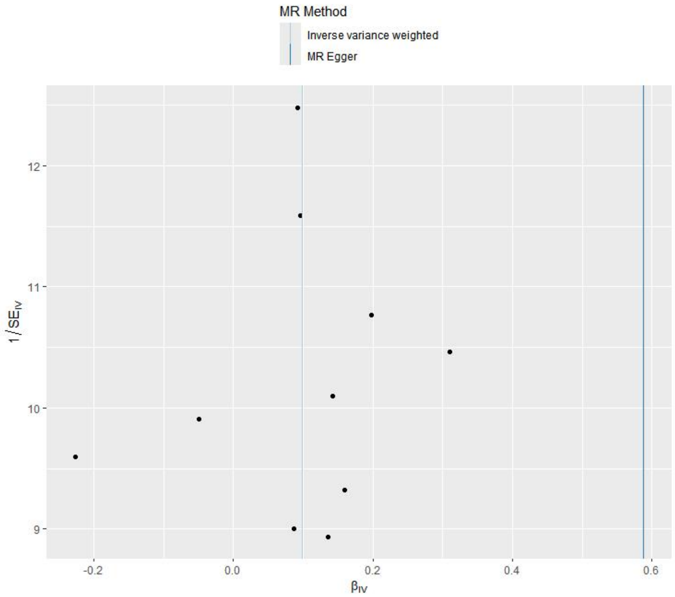
MR Method
Inverse variance weighted
MR Egger
1/SE$_{IV}$
12
11
10
9
-0.2
0.0
0.2
0.4
0.6
β$_{IV}$

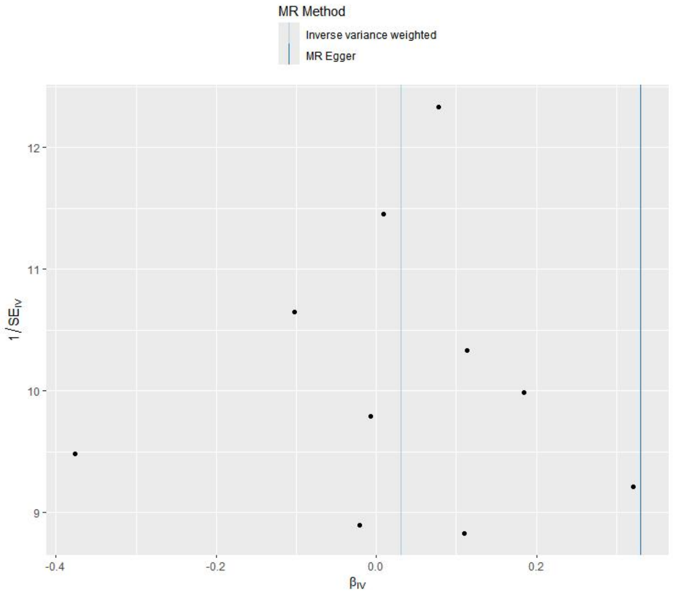
MR Method
Inverse variance weighted
MR Egger
1/SE$_{IV}$
12
11
10
9
-0.4
-0.2
0.0
0.2
β$_{IV}$

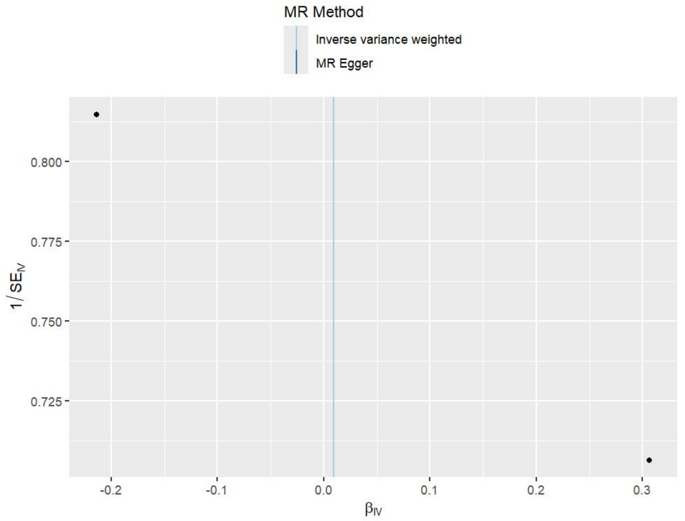
MR Method
Inverse variance weighted
MR Egger
1/SE$_{IV}$
0.800
0.775
0.750
0.725
-0.2
-0.1
0.0
0.1
0.2
0.3
β$_{IV}$

Exposure: Medical abortion

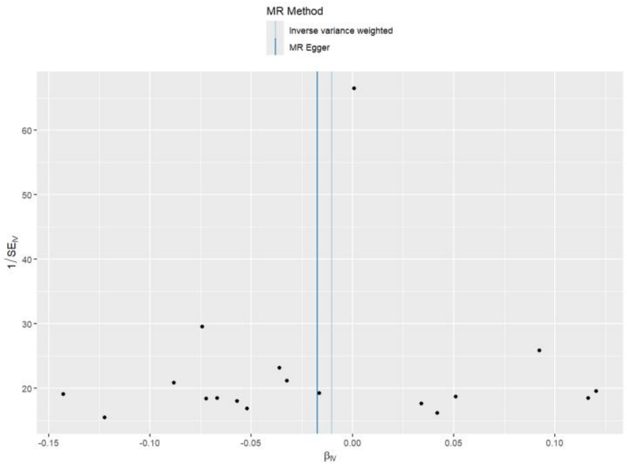

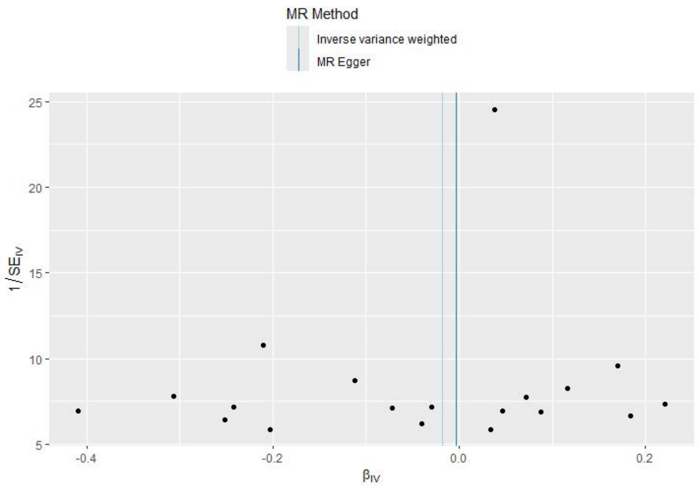

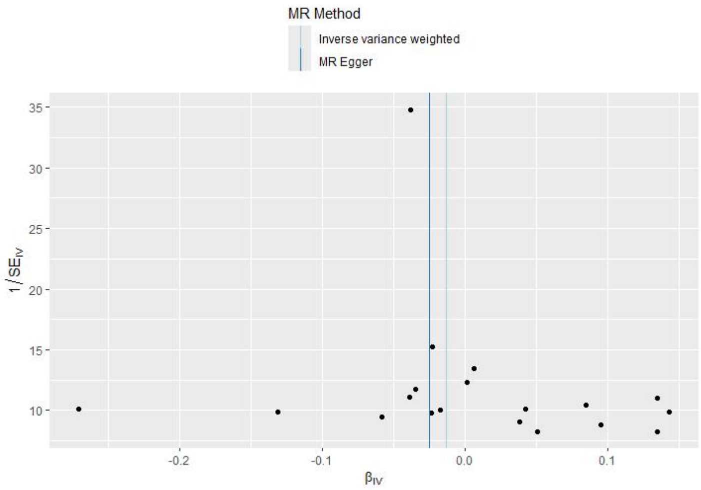

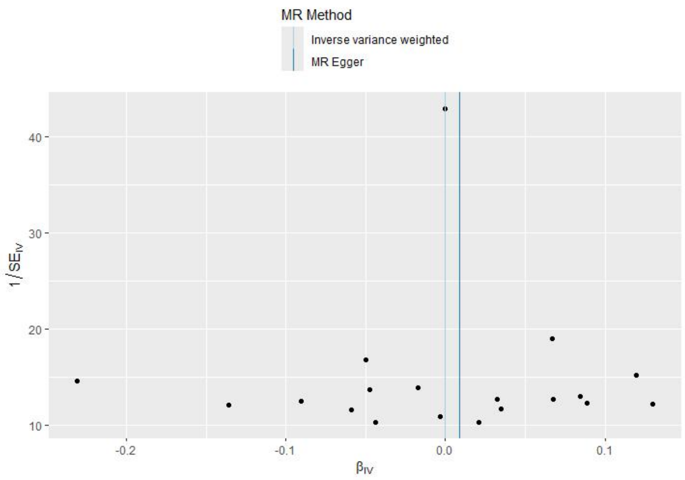

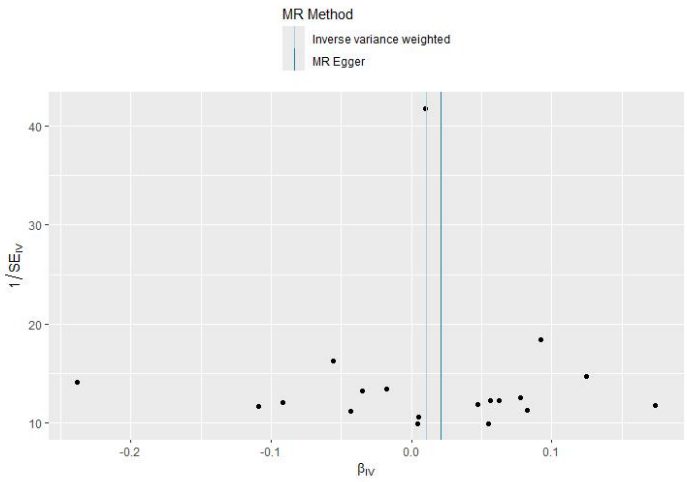

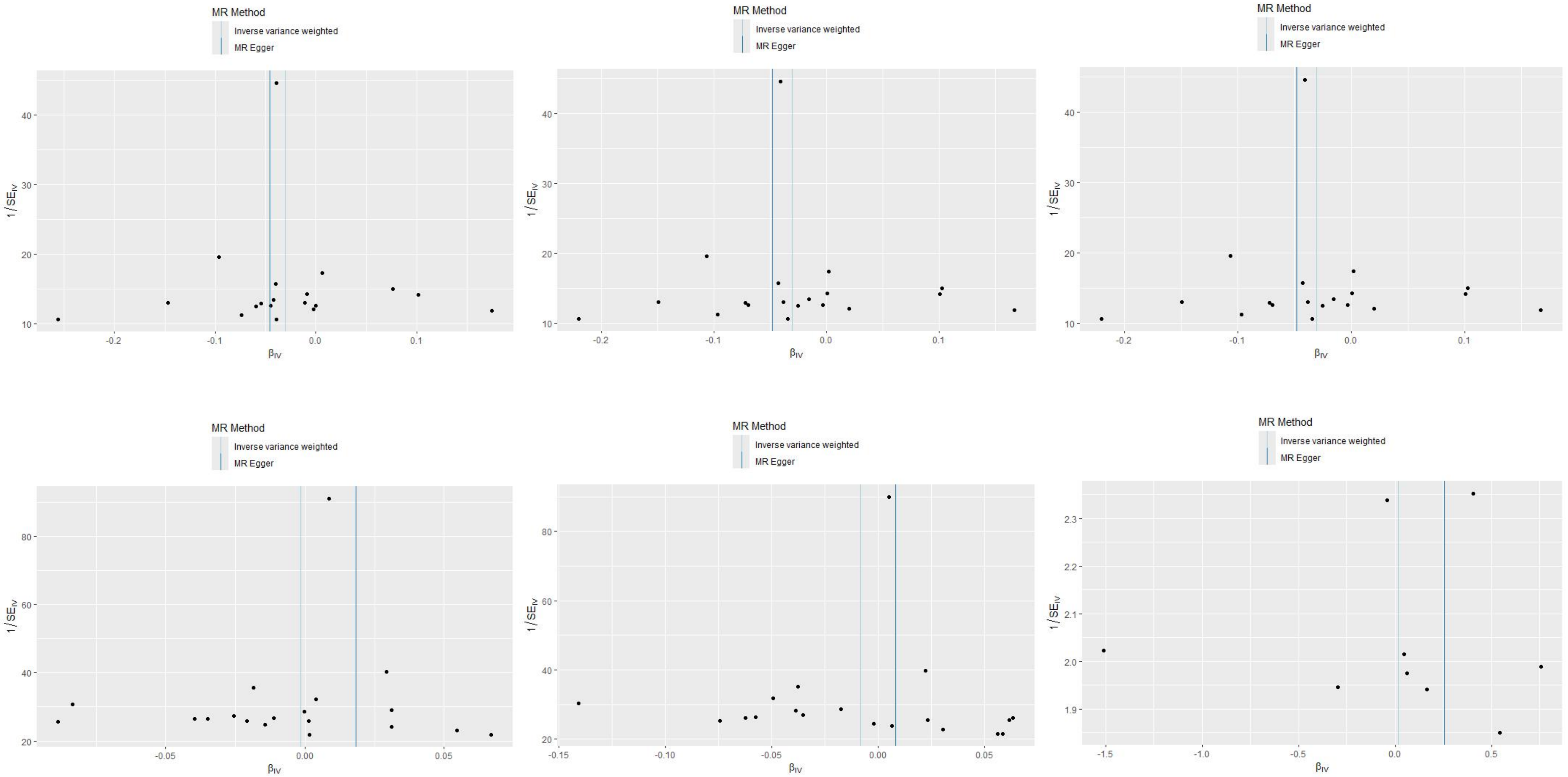
MR Method
Inverse variance weighted
MR Egger
$1/SE_{IV}$
β_{IV}
MR Method
Inverse variance weighted
MR Egger
$1/SE_{IV}$
β_{IV}
MR Method
Inverse variance weighted
MR Egger
$1/SE_{IV}$
β_{IV}
MR Method
Inverse variance weighted
MR Egger
$1/SE_{IV}$
β_{IV}
MR Method
Inverse variance weighted
MR Egger
$1/SE_{IV}$
β_{IV}
MR Method
Inverse variance weighted
MR Egger
$1/SE_{IV}$
β_{IV}

Exposure: Ever taken oral contraceptive pill

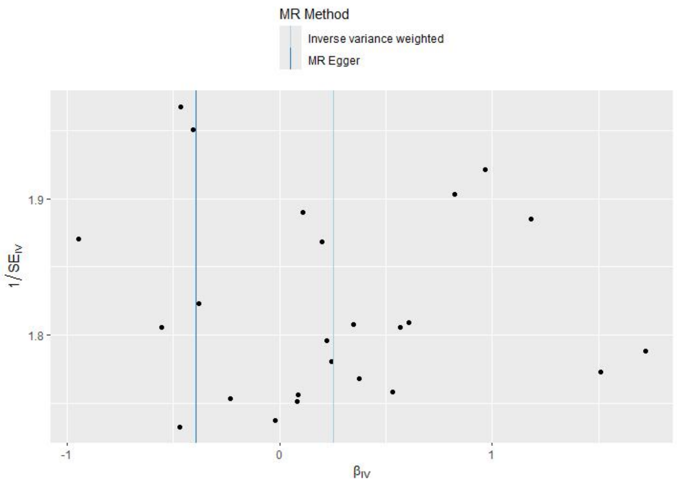

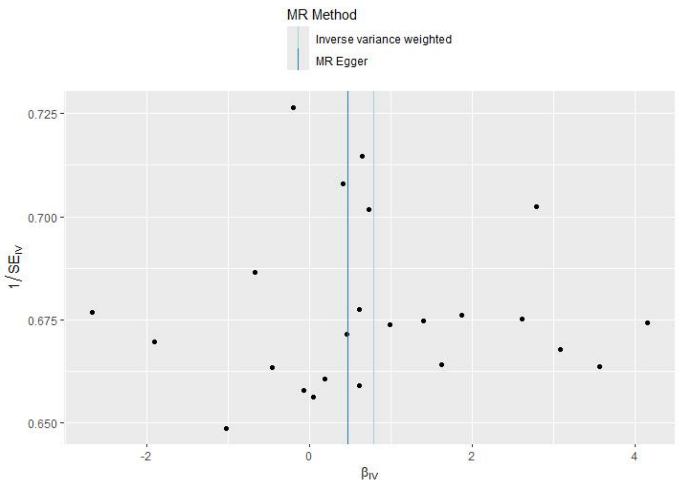

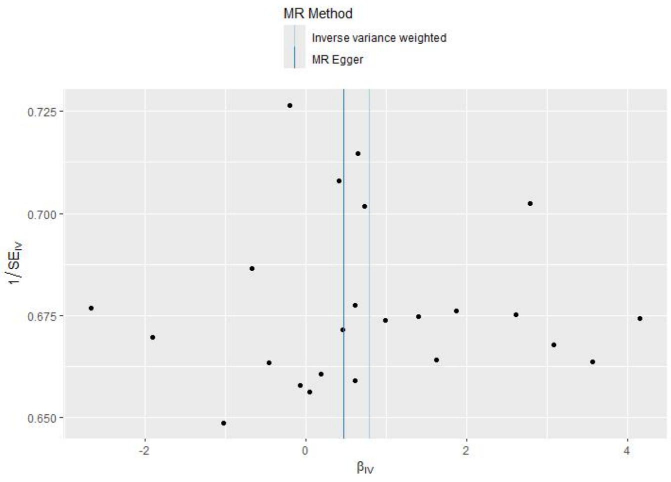

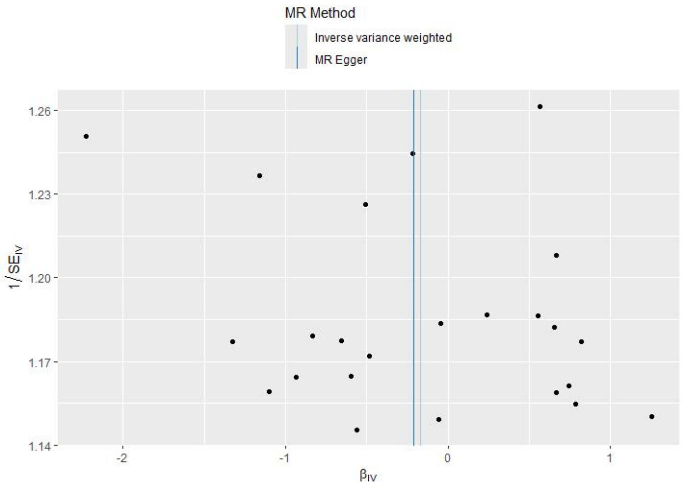

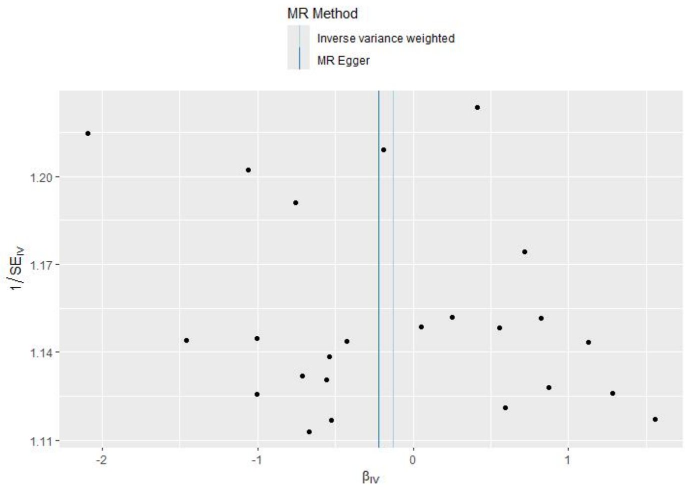

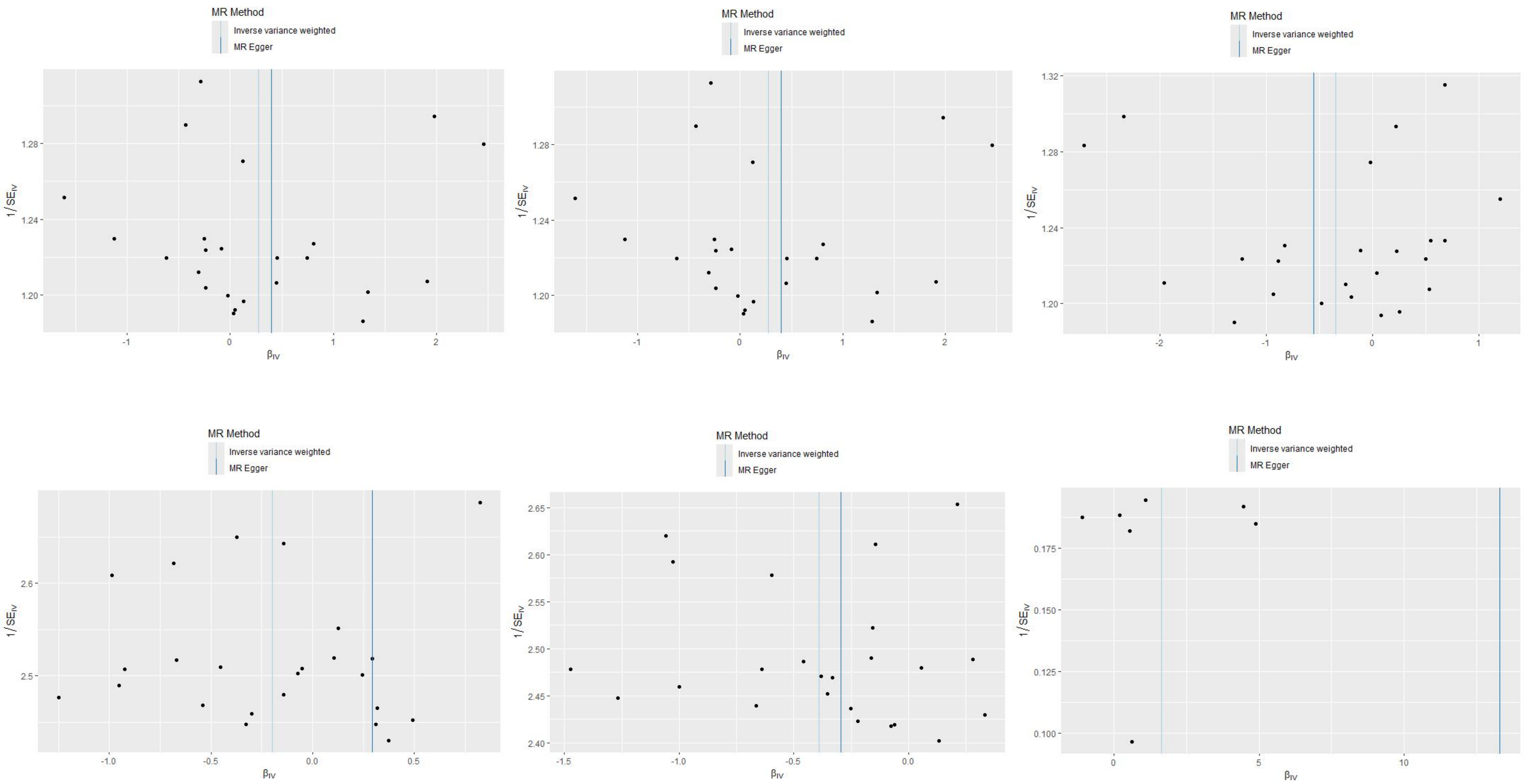
MR Method
Inverse variance weighted
MR Egger
1/SE_IV
β_IV

Exposure: Ever used hormone-replacement therapy

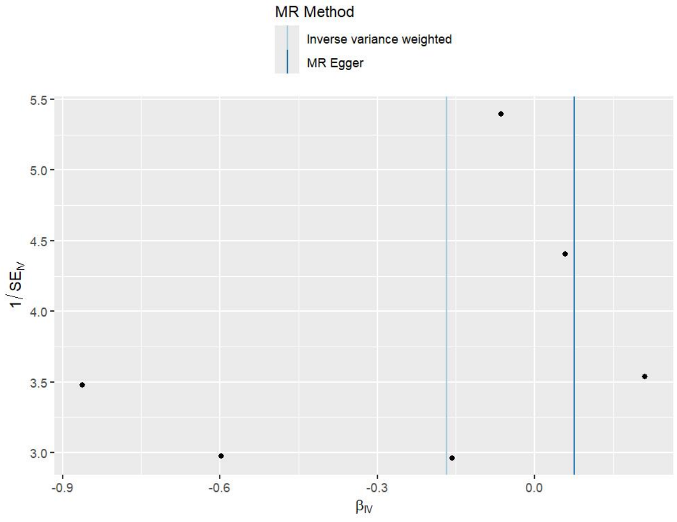

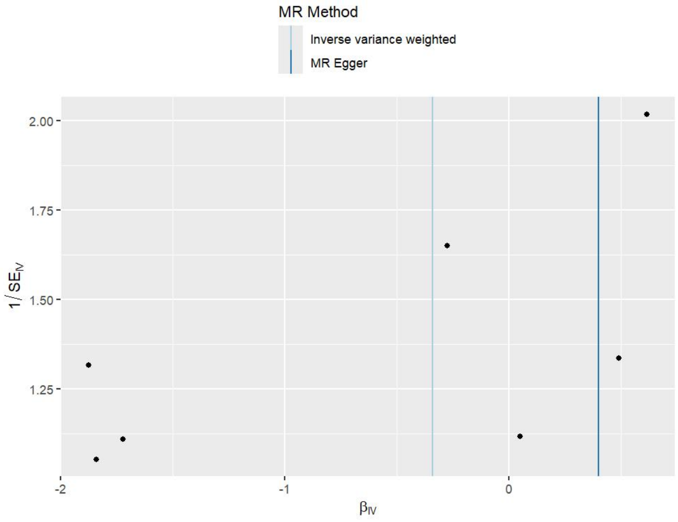

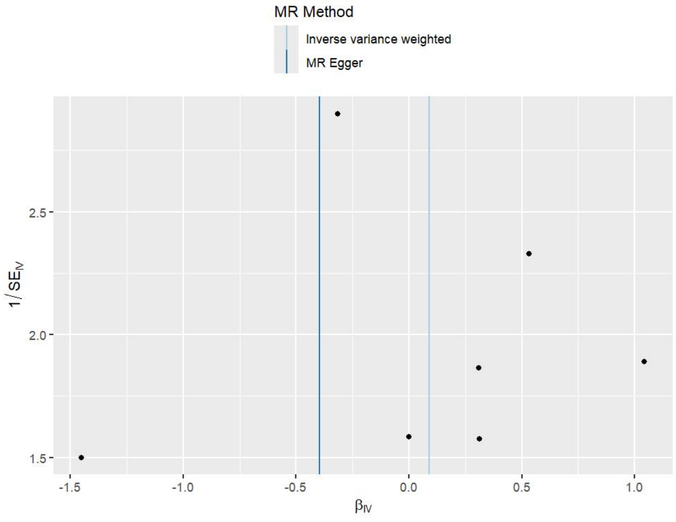

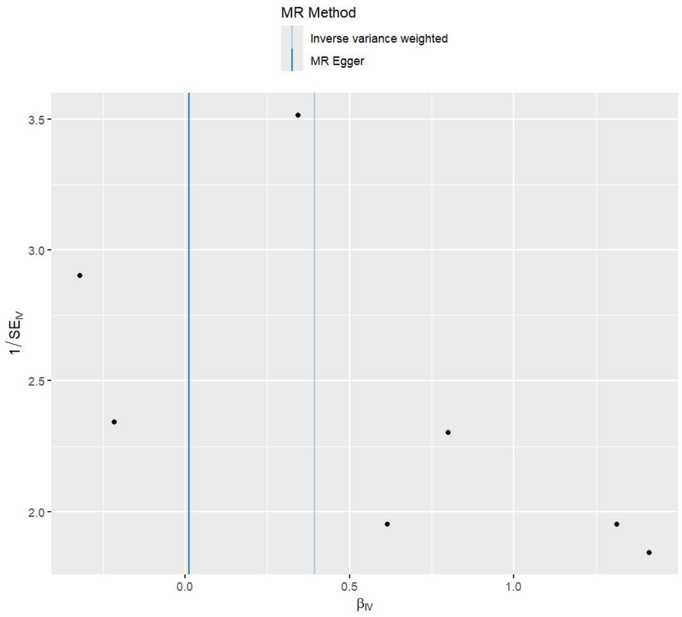

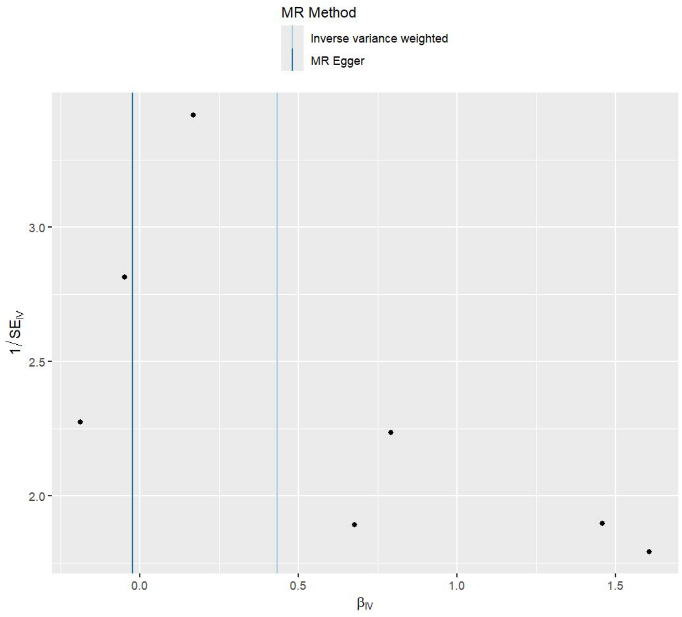

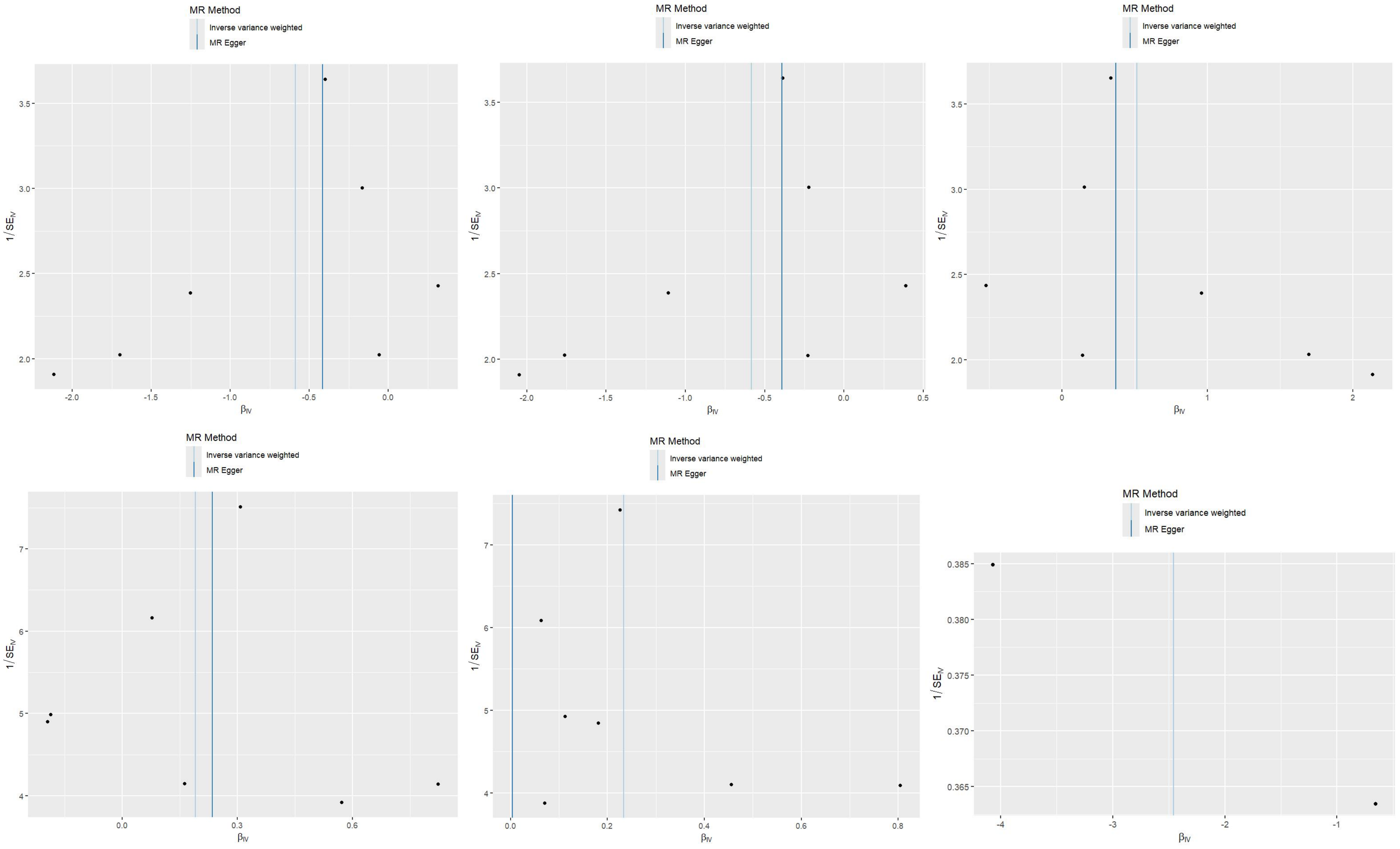
MR Method
Inverse variance weighted
MR Egger
1/SE$_{IV}$
β$_{IV}$
MR Method
Inverse variance weighted
MR Egger
1/SE$_{IV}$
β$_{IV}$
MR Method
Inverse variance weighted
MR Egger
1/SE$_{IV}$
β$_{IV}$
MR Method
Inverse variance weighted
MR Egger
1/SE$_{IV}$
β$_{IV}$
MR Method
Inverse variance weighted
MR Egger
1/SE$_{IV}$
β$_{IV}$
MR Method
Inverse variance weighted
MR Egger
1/SE$_{IV}$
β$_{IV}$

Figure S4. Forest plots depicting the results of sensitivity analyses using a leave-one-out approach to assess the influence of reproductive traits on cognitive function.

Exposure: Age at menarche

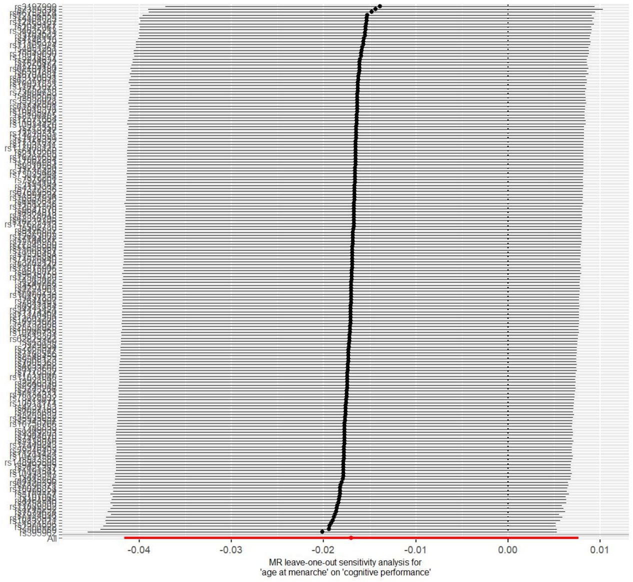

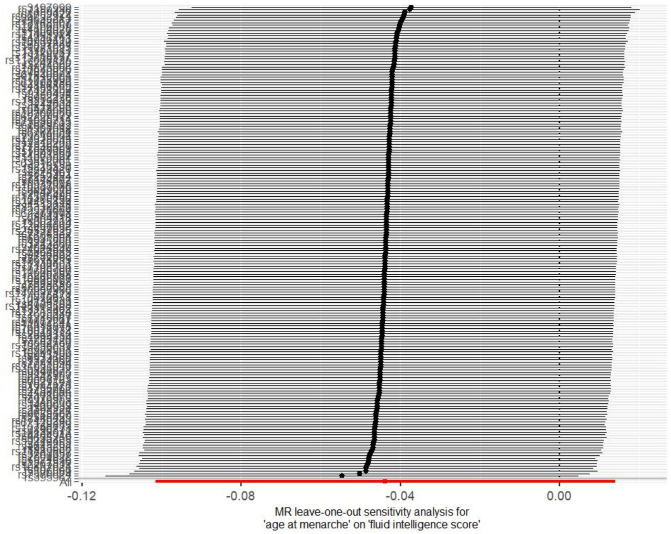

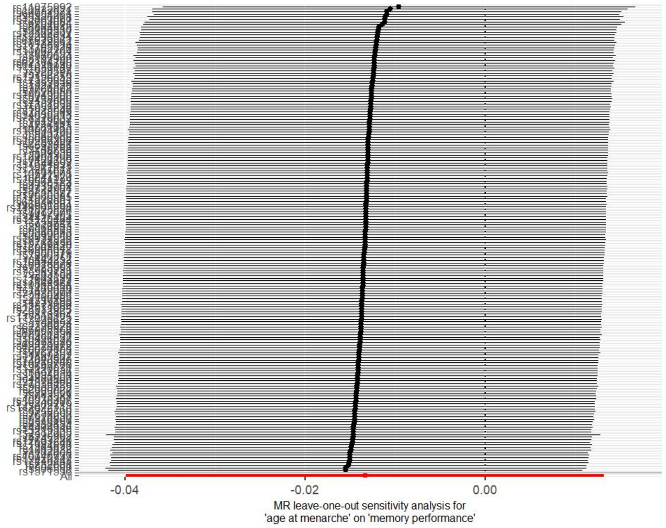

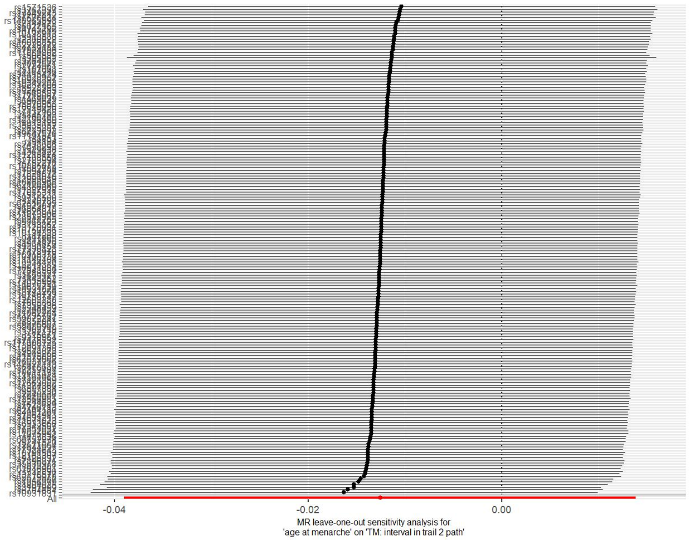

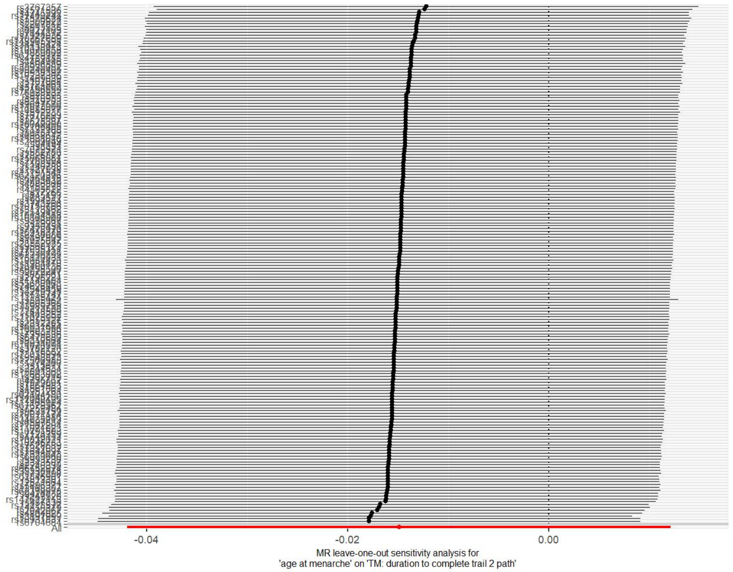

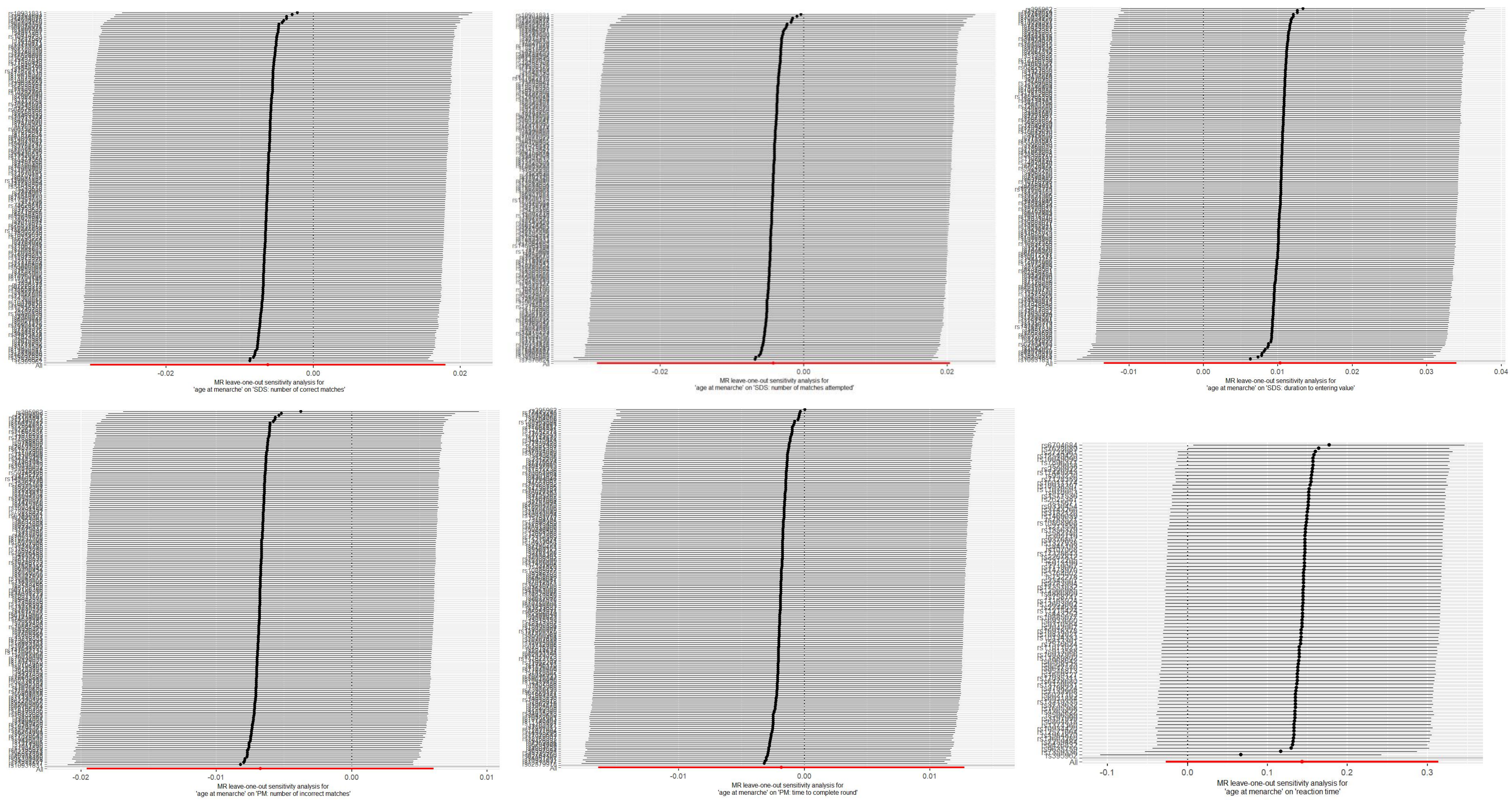

MR leave-one-out sensitivity analysis for
'age at menarche' on 'SDS: number of correct matches'
MR leave-one-out sensitivity analysis for
'age at menarche' on 'SDS: number of matches attempted'
MR leave-one-out sensitivity analysis for
'age at menarche' on 'SDS: duration to entering value'
MR leave-one-out sensitivity analysis for
'age at menarche' on 'PM: number of incorrect matches'
MR leave-one-out sensitivity analysis for
'age at menarche' on 'PM: time to complete round'
MR leave-one-out sensitivity analysis for
'age at menarche' on 'reaction time'
All

Exposure: Age at menopause

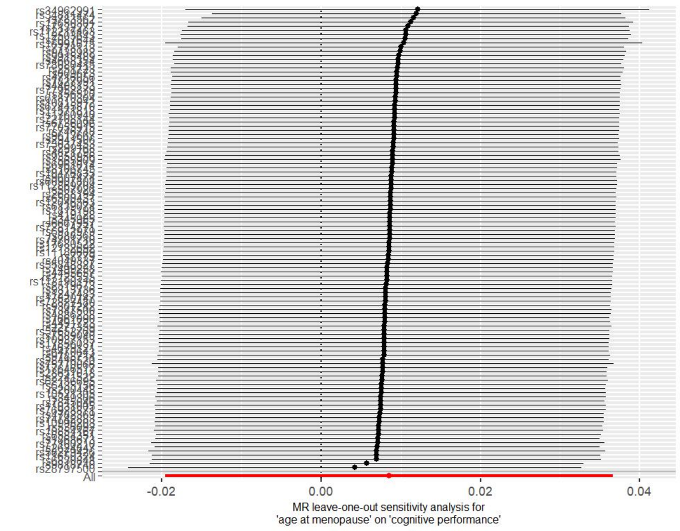

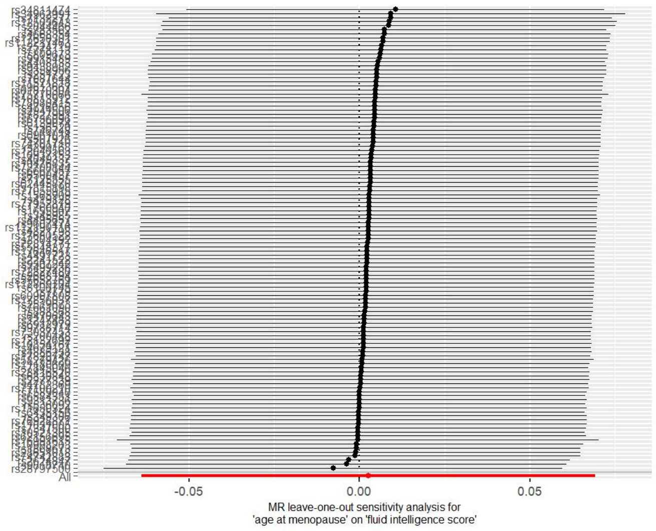

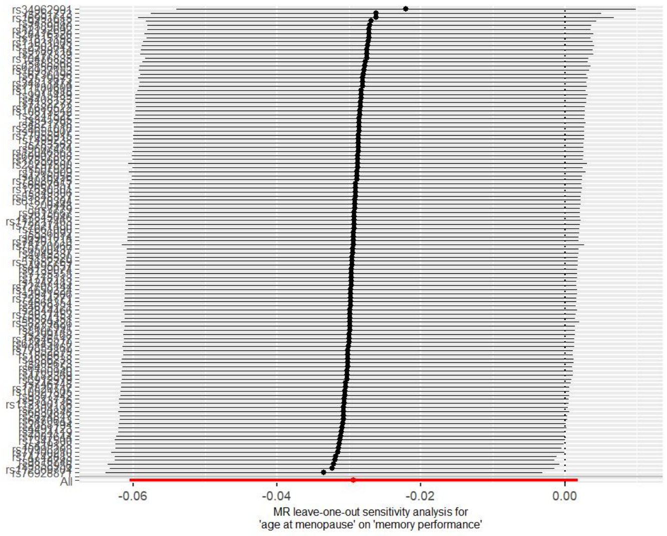

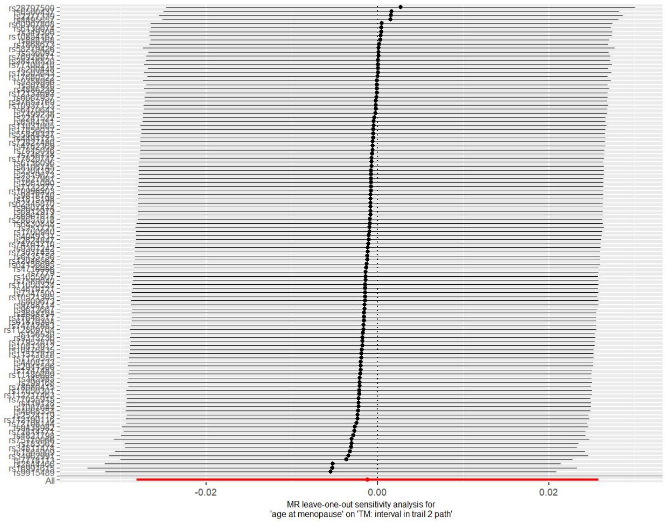

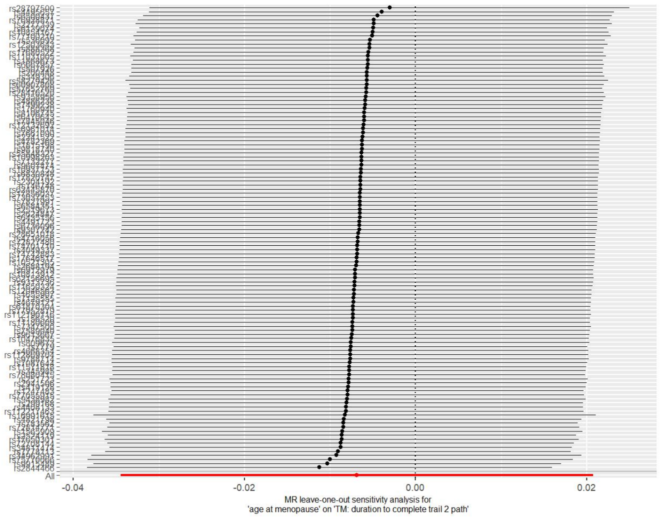

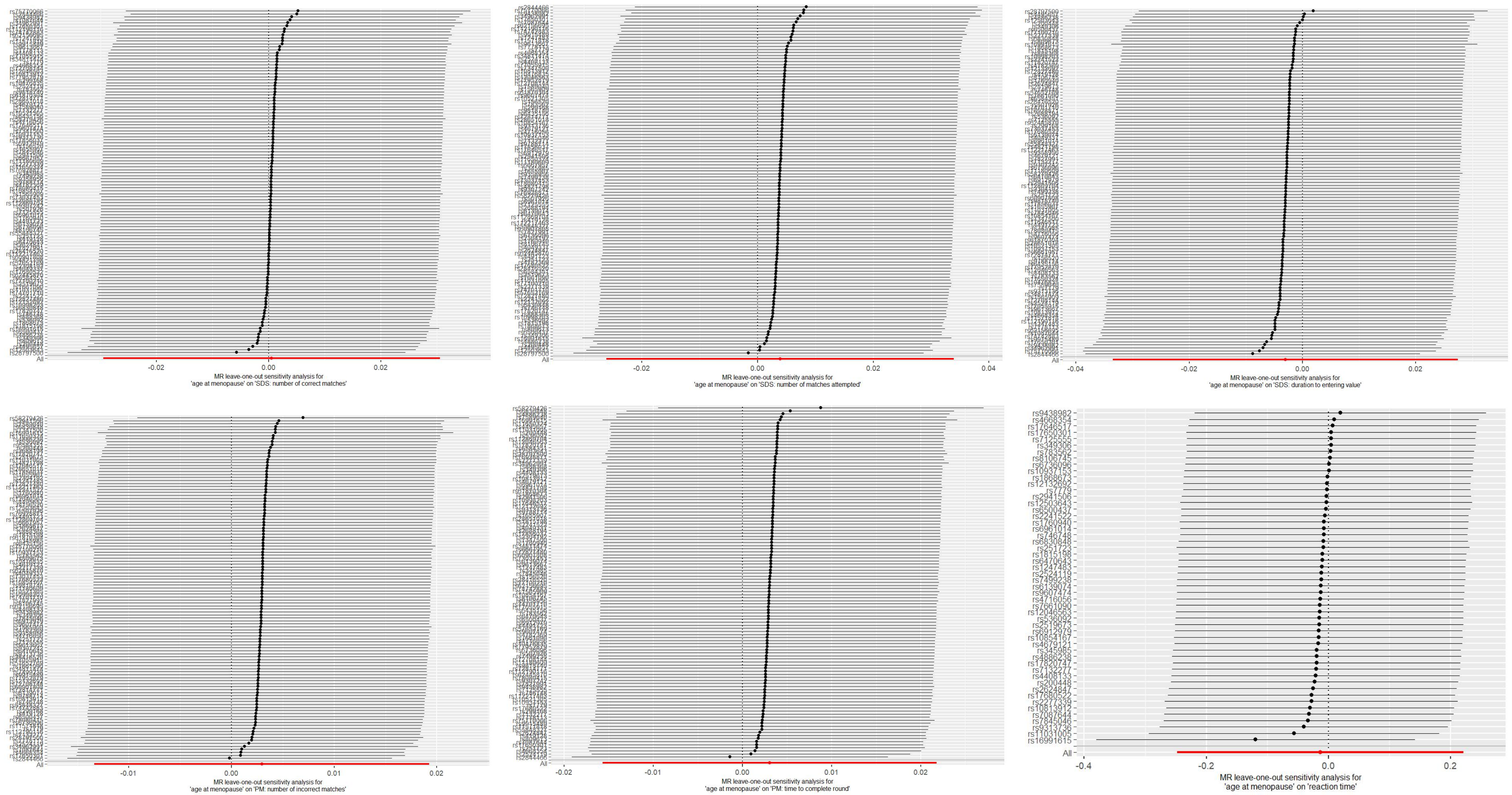

MR leave-one-out sensitivity analysis for
'age at menopause' on 'SDS: number of correct matches'
-0.02
0.00
0.02
All
MR leave-one-out sensitivity analysis for
'age at menopause' on 'SDS: number of matches attempted'
-0.02
0.00
0.02
0.04
All
MR leave-one-out sensitivity analysis for
'age at menopause' on 'SDS: duration to entering value'
-0.04
-0.02
0.00
0.02
All
MR leave-one-out sensitivity analysis for
'age at menopause' on 'PM: number of incorrect matches'
-0.01
0.00
0.01
0.02
All
MR leave-one-out sensitivity analysis for
'age at menopause' on 'PM: time to complete round'
-0.02
-0.01
0.00
0.01
0.02
All
MR leave-one-out sensitivity analysis for
'age at menopause' on 'reaction time'
-0.4
-0.2
0.0
0.2
All

Exposure: Age at first sexual intercourse

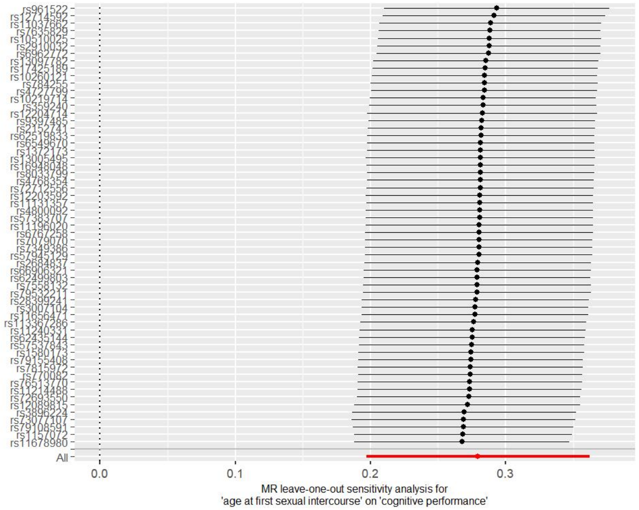

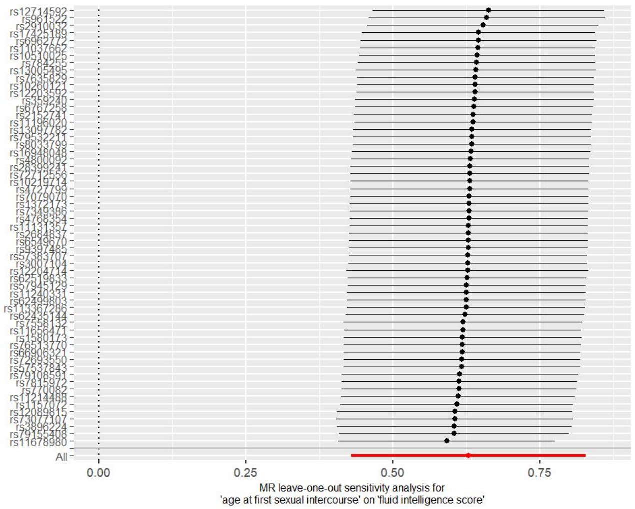

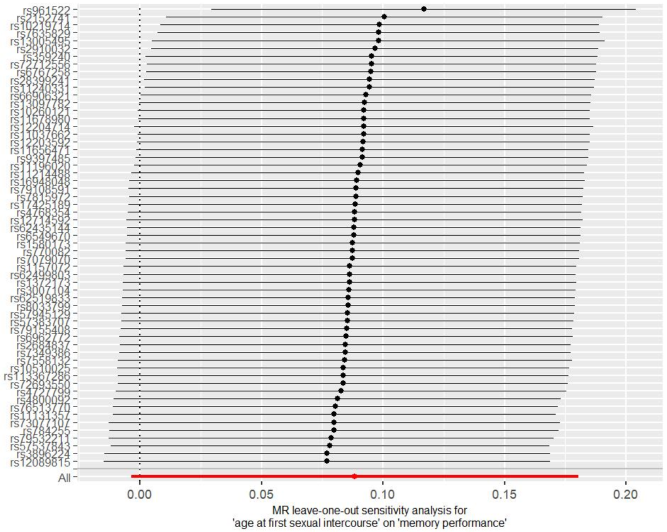

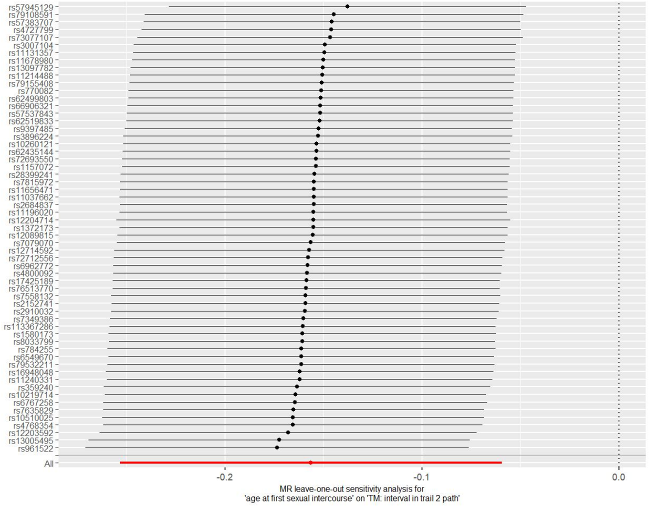

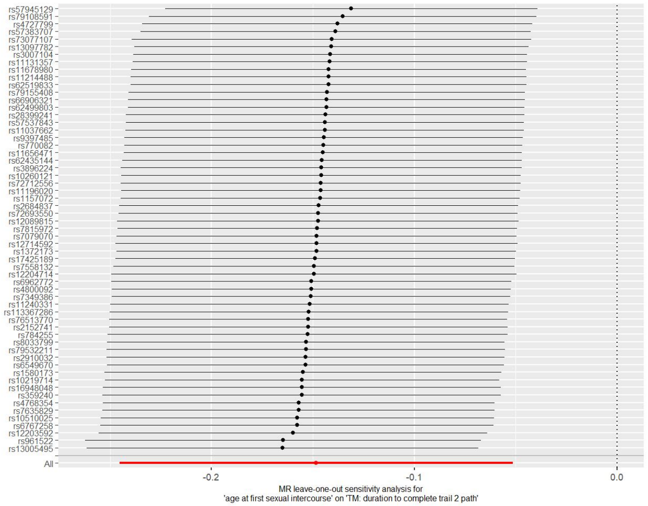

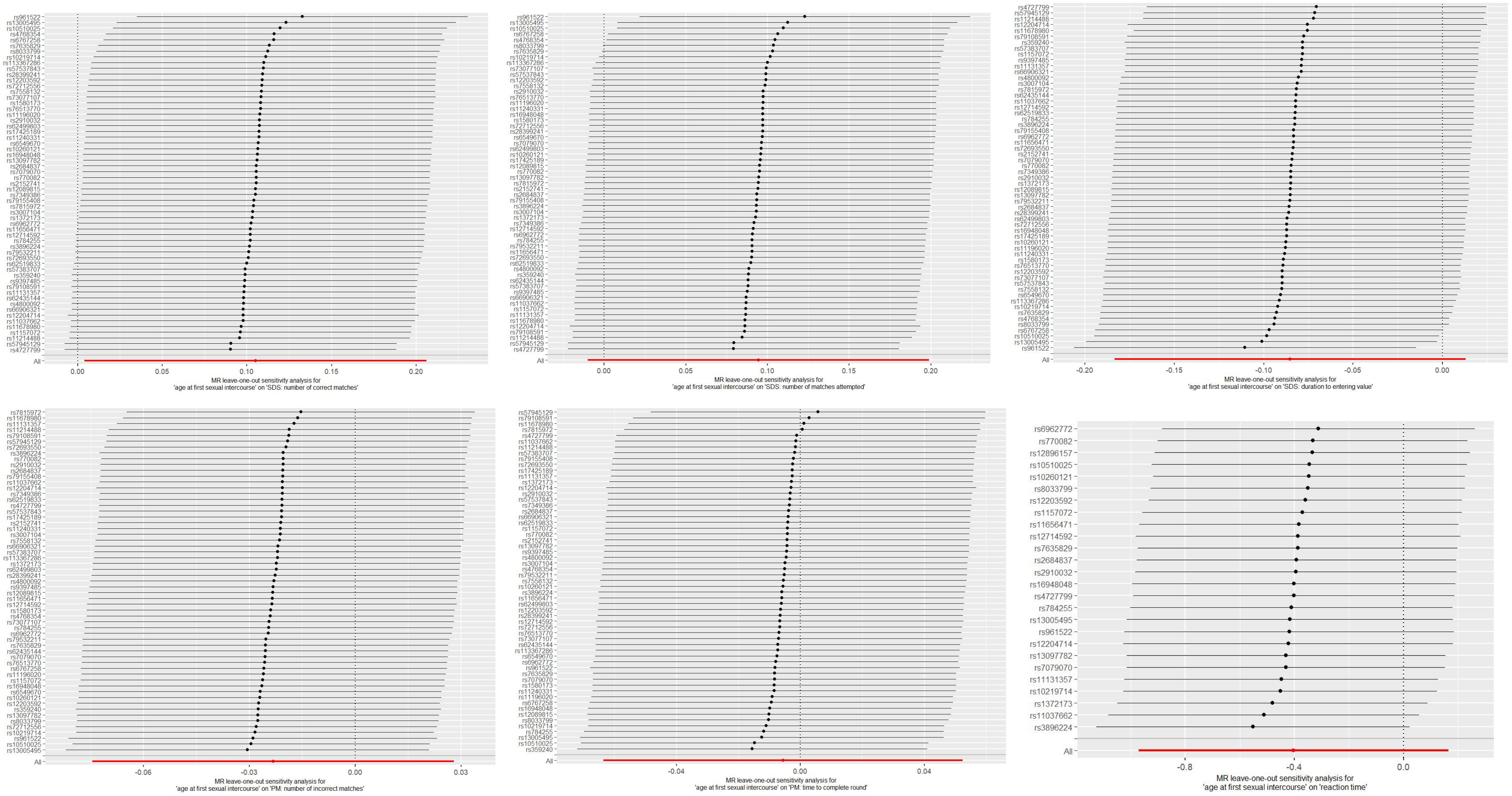

MR leave-one-out sensitivity analysis for
'age at first sexual intercourse' on 'SDS: number of correct matches'
0.00 0.05 0.10 0.15 0.20
MR leave-one-out sensitivity analysis for
'age at first sexual intercourse' on 'SDS: number of matches attempted'
0.00 0.05 0.10 0.15 0.20
MR leave-one-out sensitivity analysis for
'age at first sexual intercourse' on 'SDS: duration to entering value'
-0.20 -0.15 -0.10 -0.05 0.00
MR leave-one-out sensitivity analysis for
'age at first sexual intercourse' on 'PM: number of incorrect matches'
-0.06 -0.03 0.00 0.03
MR leave-one-out sensitivity analysis for
'age at first sexual intercourse' on 'PM: time to complete round'
-0.04 0.00 0.04
MR leave-one-out sensitivity analysis for
'age at first sexual intercourse' on 'reaction time'
-0.8 -0.4 0.0
All

Exposure: Age at first birth

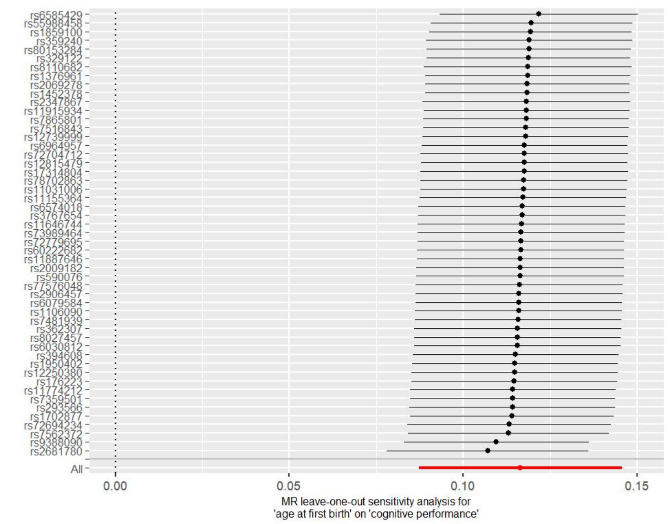

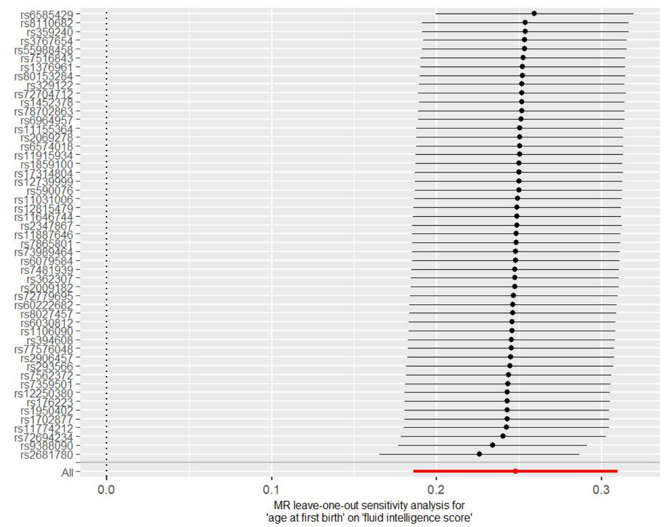

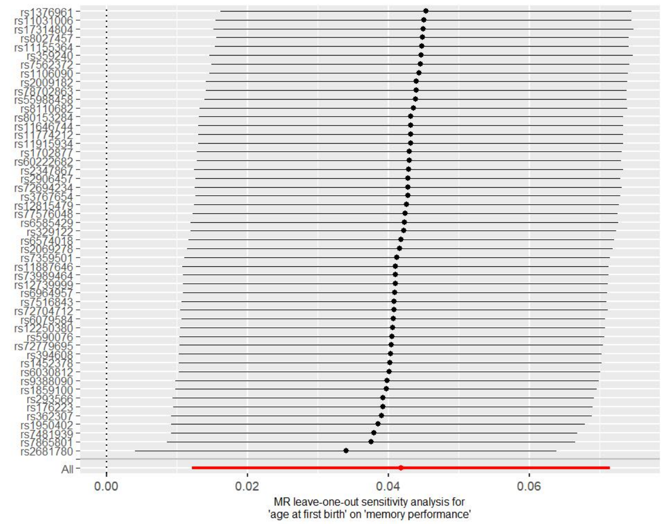

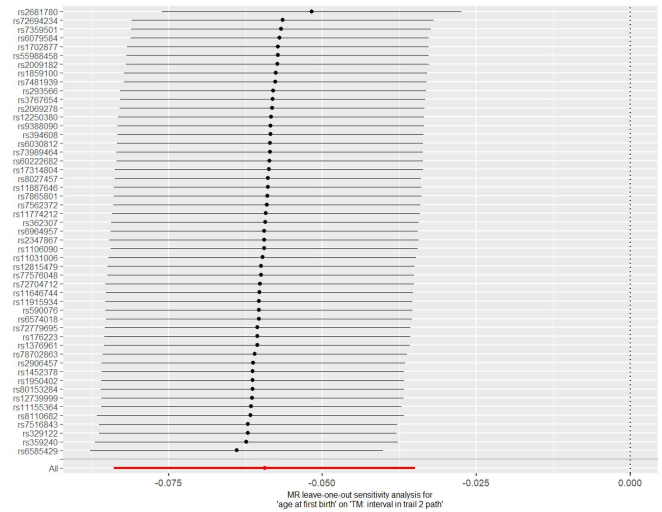

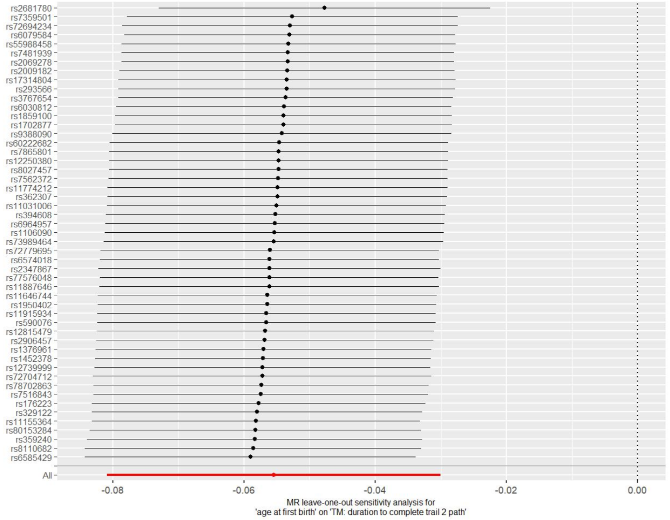

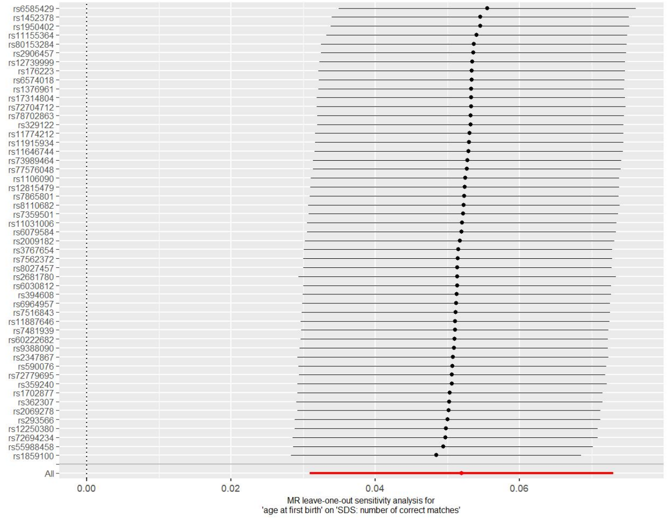
rs6585429
rs1452378
rs1950402
rs11155364
rs80153284
rs2906457
rs12739999
rs176223
rs6574018
rs1376961
rs17314804
rs72704712
rs78702863
rs329122
rs11774212
rs11915934
rs11646744
rs73989464
rs77576048
rs1106090
rs12815479
rs7865801
rs8110682
rs7359501
rs11031006
rs6079584
rs2009182
rs3767654
rs7562372
rs8027457
rs2681780
rs6030812
rs394608
rs6964957
rs7516843
rs11887646
rs7481939
rs60222682
rs9388090
rs2347867
rs590076
rs72779695
rs359240
rs1702877
rs362307
rs2069278
rs293566
rs12250380
rs72694234
rs55988458
rs1859100
All
0.00
0.02
0.04
0.06
MR leave-one-out sensitivity analysis for
'age at first birth' on 'SDS: number of correct matches'

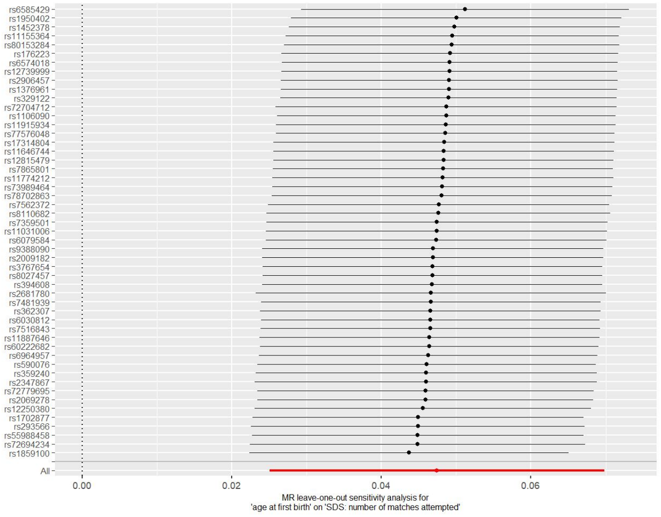
rs6585429
rs1950402
rs1452378
rs11155364
rs80153284
rs176223
rs6574018
rs12739999
rs2906457
rs1376961
rs329122
rs72704712
rs1106090
rs11915934
rs77576048
rs17314804
rs11646744
rs12815479
rs7865801
rs11774212
rs73989464
rs78702863
rs7562372
rs8110682
rs7359501
rs11031006
rs6079584
rs9388090
rs2009182
rs3767654
rs8027457
rs394608
rs2681780
rs7481939
rs362307
rs6030812
rs7516843
rs11887646
rs60222682
rs6964957
rs590076
rs359240
rs2347867
rs72779695
rs2069278
rs12250380
rs1702877
rs293566
rs55988458
rs72694234
rs1859100
All
0.00
0.02
0.04
0.06
MR leave-one-out sensitivity analysis for
'age at first birth' on 'SDS: number of matches attempted'

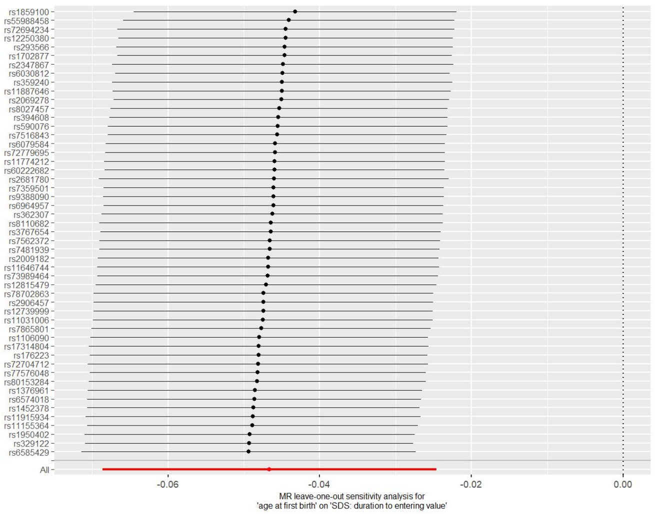
rs1859100
rs55988458
rs72694234
rs12250380
rs293566
rs1702877
rs2347867
rs6030812
rs359240
rs11887646
rs2069278
rs8027457
rs394608
rs590076
rs7516843
rs6079584
rs72779695
rs11774212
rs60222682
rs2681780
rs7359501
rs9388090
rs6964957
rs362307
rs8110682
rs3767654
rs7562372
rs7481939
rs2009182
rs11646744
rs73989464
rs12815479
rs78702863
rs2906457
rs12739999
rs11031006
rs7865801
rs1106090
rs17314804
rs176223
rs72704712
rs77576048
rs80153284
rs1376961
rs6574018
rs1452378
rs11915934
rs11155364
rs1950402
rs329122
rs6585429
All
-0.06
-0.04
-0.02
0.00
MR leave-one-out sensitivity analysis for
'age at first birth' on 'SDS: duration to entering value'

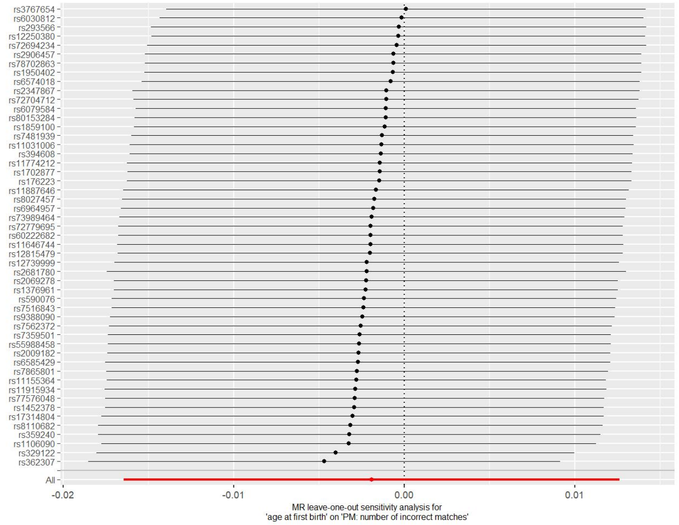
rs3767654
rs6030812
rs293566
rs12250380
rs72694234
rs2906457
rs78702863
rs1950402
rs6574018
rs2347867
rs72704712
rs6079584
rs80153284
rs1859100
rs7481939
rs11031006
rs394608
rs11774212
rs1702877
rs176223
rs11887646
rs8027457
rs6964957
rs73989464
rs72779695
rs60222682
rs11646744
rs12815479
rs12739999
rs2681780
rs2069278
rs1376961
rs590076
rs7516843
rs9388090
rs7562372
rs7359501
rs55988458
rs2009182
rs6585429
rs7865801
rs11155364
rs11915934
rs77576048
rs1452378
rs17314804
rs8110682
rs359240
rs1106090
rs329122
rs362307
All
-0.02
-0.01
0.00
0.01
MR leave-one-out sensitivity analysis for
'age at first birth' on 'PM: number of incorrect matches'

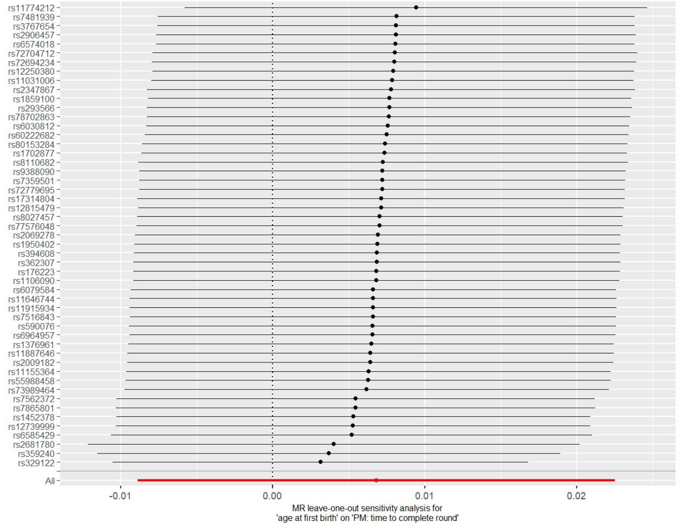
rs11774212
rs7481939
rs3767654
rs2906457
rs6574018
rs72704712
rs72694234
rs12250380
rs11031006
rs2347867
rs1859100
rs293566
rs78702863
rs6030812
rs60222682
rs80153284
rs1702877
rs8110682
rs9388090
rs7359501
rs72779695
rs17314804
rs12815479
rs8027457
rs77576048
rs2069278
rs1950402
rs394608
rs362307
rs176223
rs1106090
rs6079584
rs11646744
rs11915934
rs7516843
rs590076
rs6964957
rs1376961
rs11887646
rs2009182
rs11155364
rs55988458
rs73989464
rs7562372
rs7865801
rs1452378
rs12739999
rs6585429
rs2681780
rs359240
rs329122
All
-0.01
0.00
0.01
0.02
MR leave-one-out sensitivity analysis for
'age at first birth' on 'PM: time to complete round'

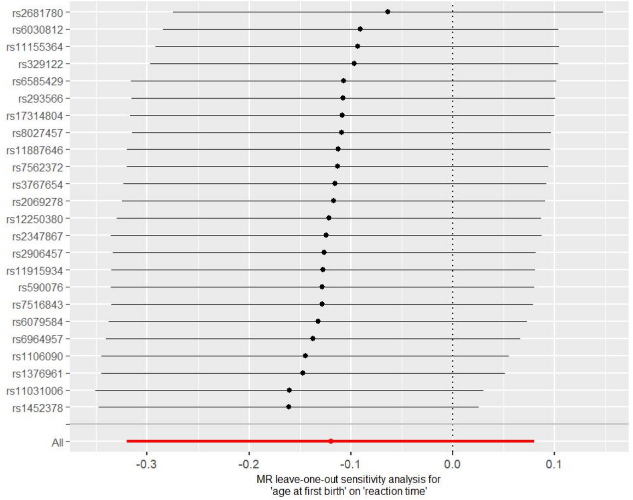
rs2681780
rs6030812
rs11155364
rs329122
rs6585429
rs293566
rs17314804
rs8027457
rs11887646
rs7562372
rs3767654
rs2069278
rs12250380
rs2347867
rs2906457
rs11915934
rs590076
rs7516843
rs6079584
rs6964957
rs1106090
rs1376961
rs11031006
rs1452378
All
-0.3
-0.2
-0.1
0.0
0.1
MR leave-one-out sensitivity analysis for
'age at first birth' on 'reaction time'

Exposure: Birth weight of first child

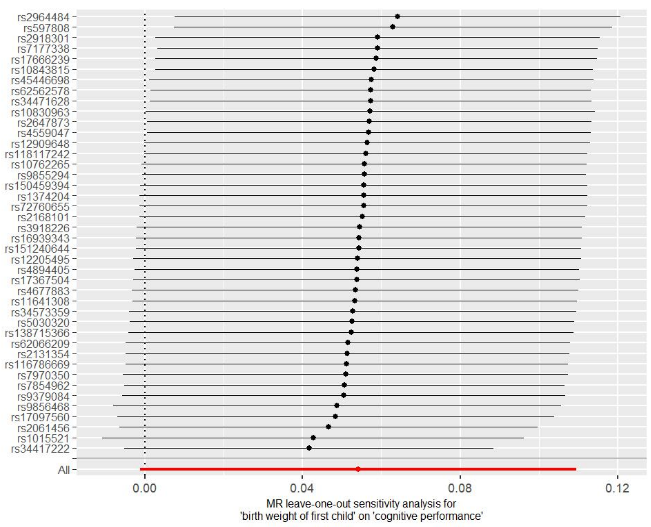

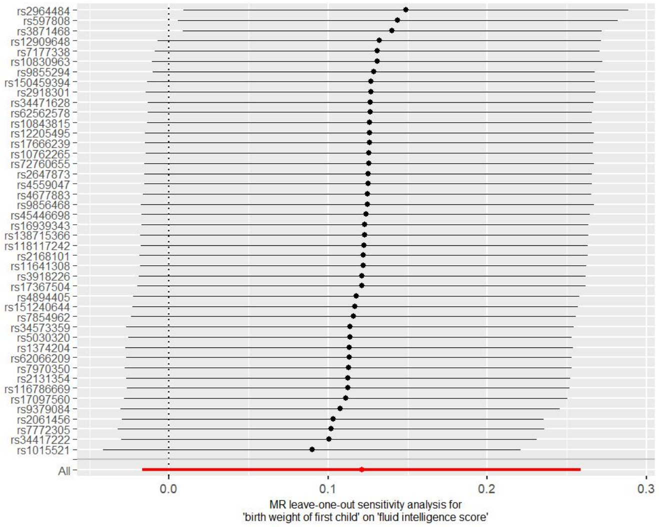

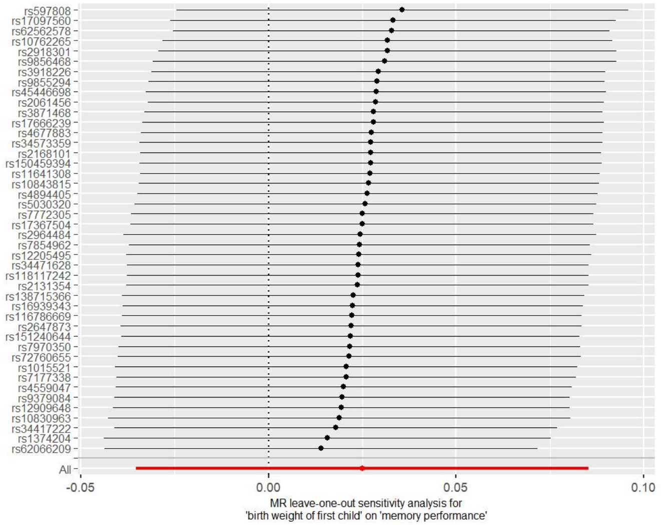

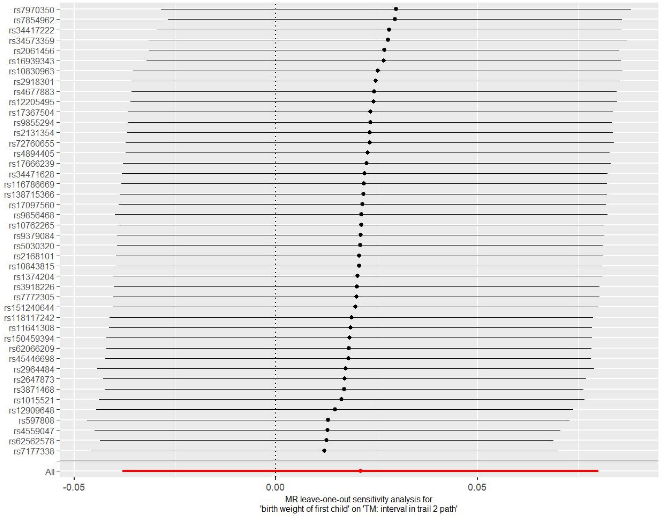

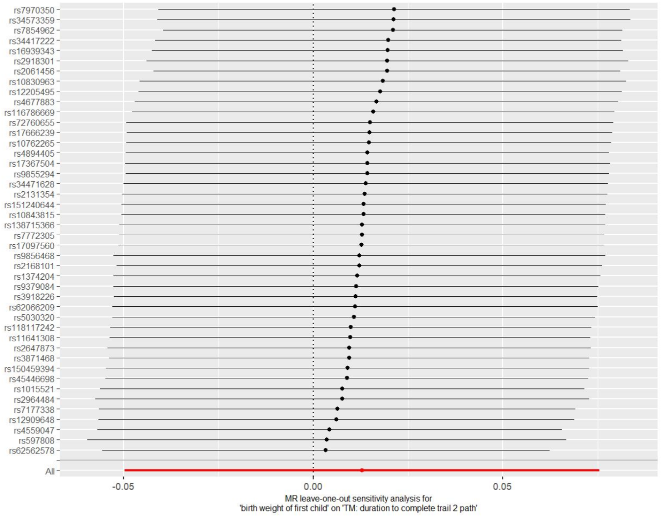

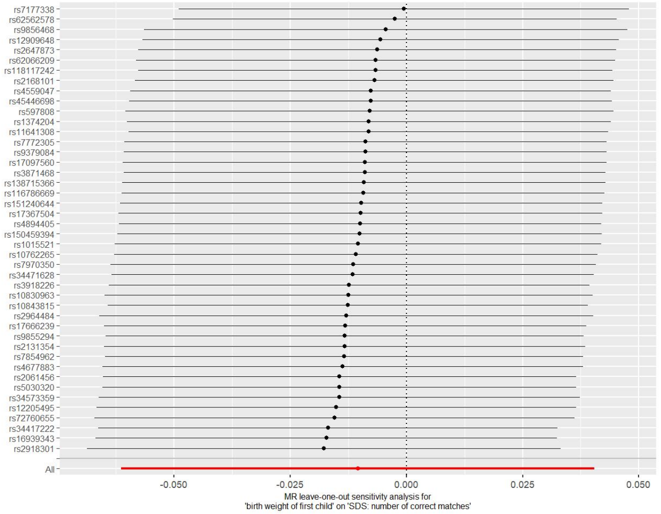
rs7177338
rs62562578
rs9856468
rs12909648
rs2647873
rs62066209
rs118117242
rs2168101
rs4559047
rs45446698
rs597808
rs1374204
rs11641308
rs7772305
rs9379084
rs17097560
rs3871468
rs138715366
rs116786669
rs151240644
rs17367504
rs4894405
rs150459394
rs1015521
rs10762265
rs7970350
rs34471628
rs3918226
rs10830963
rs10843815
rs2964484
rs17666239
rs9855294
rs2131354
rs7854962
rs4677883
rs2061456
rs5030320
rs34573359
rs12205495
rs72760655
rs34417222
rs16939343
rs2918301
All
-0.050
-0.025
0.000
0.025
0.050
MR leave-one-out sensitivity analysis for
'birth weight of first child' on 'SDS: number of correct matches'

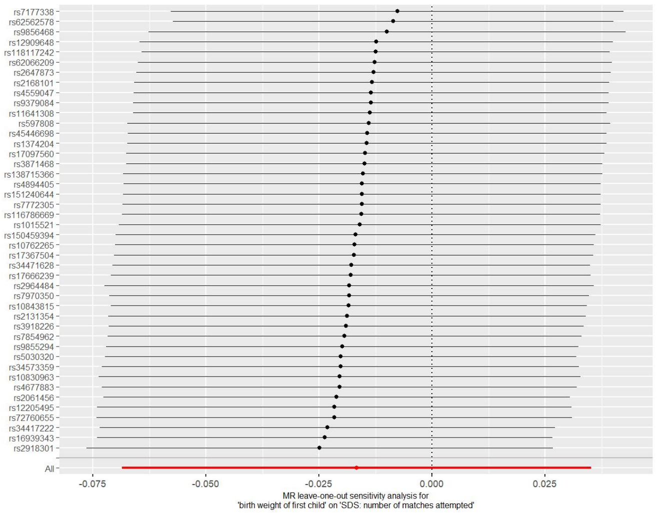
rs7177338
rs62562578
rs9856468
rs12909648
rs118117242
rs62066209
rs2647873
rs2168101
rs4559047
rs9379084
rs11641308
rs597808
rs45446698
rs1374204
rs17097560
rs3871468
rs138715366
rs4894405
rs151240644
rs7772305
rs116786669
rs1015521
rs150459394
rs10762265
rs17367504
rs34471628
rs17666239
rs2964484
rs7970350
rs10843815
rs2131354
rs3918226
rs7854962
rs9855294
rs5030320
rs34573359
rs10830963
rs4677883
rs2061456
rs12205495
rs72760655
rs34417222
rs16939343
rs2918301
All
-0.075
-0.050
-0.025
0.000
0.025
MR leave-one-out sensitivity analysis for
'birth weight of first child' on 'SDS: number of matches attempted'

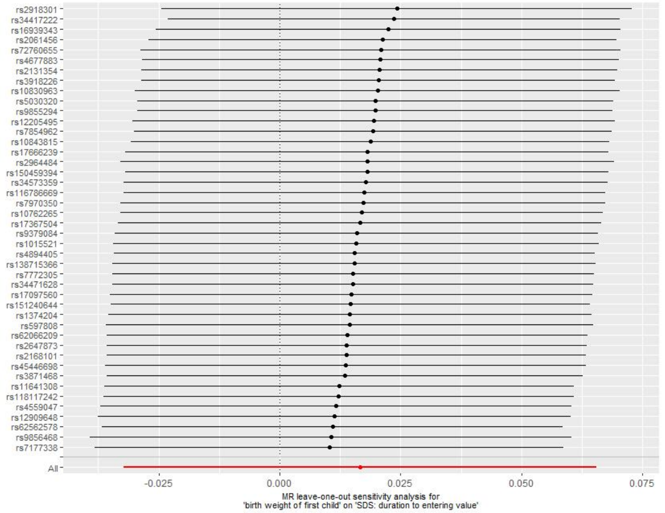
rs2918301
rs34417222
rs16939343
rs2061456
rs72760655
rs4677883
rs2131354
rs3918226
rs10830963
rs5030320
rs9855294
rs12205495
rs7854962
rs10843815
rs17666239
rs2964484
rs150459394
rs34573359
rs116786669
rs7970350
rs10762265
rs17367504
rs9379084
rs1015521
rs4894405
rs138715366
rs7772305
rs34471628
rs17097560
rs151240644
rs1374204
rs597808
rs62066209
rs2647873
rs2168101
rs45446698
rs3871468
rs11641308
rs118117242
rs4559047
rs12909648
rs62562578
rs9856468
rs7177338
All
-0.025
0.000
0.025
0.050
0.075
MR leave-one-out sensitivity analysis for
'birth weight of first child' on 'SDS: duration to entering value'

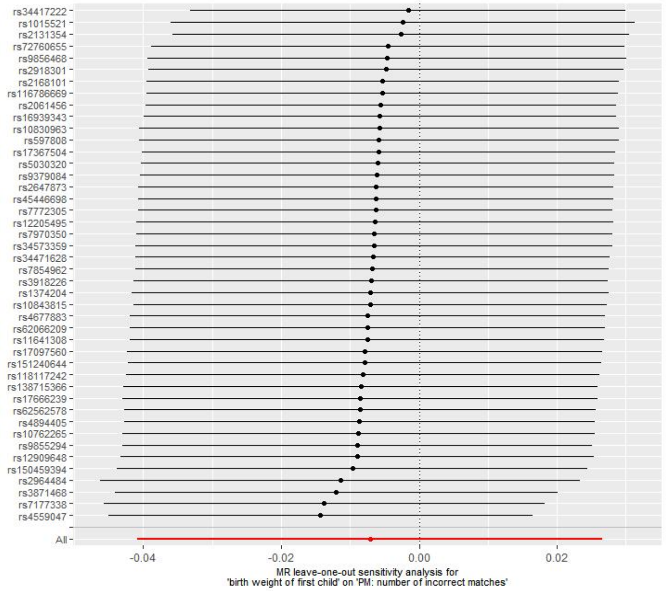
rs34417222
rs1015521
rs2131354
rs72760655
rs9856468
rs2918301
rs2168101
rs116786669
rs2061456
rs16939343
rs10830963
rs597808
rs17367504
rs5030320
rs9379084
rs2647873
rs45446698
rs7772305
rs12205495
rs7970350
rs34573359
rs34471628
rs7854962
rs3918226
rs1374204
rs10843815
rs4677883
rs62066209
rs11641308
rs17097560
rs151240644
rs118117242
rs138715366
rs17666239
rs62562578
rs4894405
rs10762265
rs9855294
rs12909648
rs150459394
rs2964484
rs3871468
rs7177338
rs4559047
All
-0.04
-0.02
0.00
0.02
MR leave-one-out sensitivity analysis for
'birth weight of first child' on 'PM: number of incorrect matches'

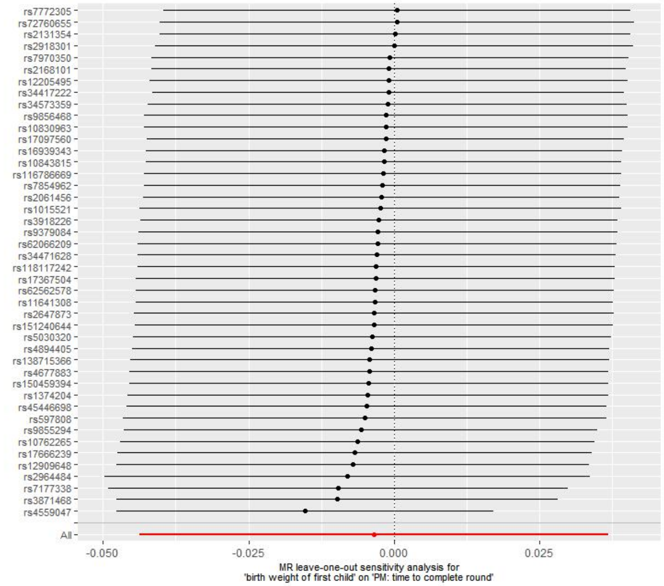
rs7772305
rs72760655
rs2131354
rs2918301
rs7970350
rs2168101
rs12205495
rs34417222
rs34573359
rs9856468
rs10830963
rs17097560
rs16939343
rs10843815
rs116786669
rs7854962
rs2061456
rs1015521
rs3918226
rs9379084
rs62066209
rs34471628
rs118117242
rs17367504
rs62562578
rs11641308
rs2647873
rs151240644
rs5030320
rs4894405
rs138715366
rs4677883
rs150459394
rs1374204
rs45446698
rs597808
rs9855294
rs10762265
rs17666239
rs12909648
rs2964484
rs7177338
rs3871468
rs4559047
All
-0.050
-0.025
0.000
0.025
MR leave-one-out sensitivity analysis for
'birth weight of first child' on 'PM: time to complete round'

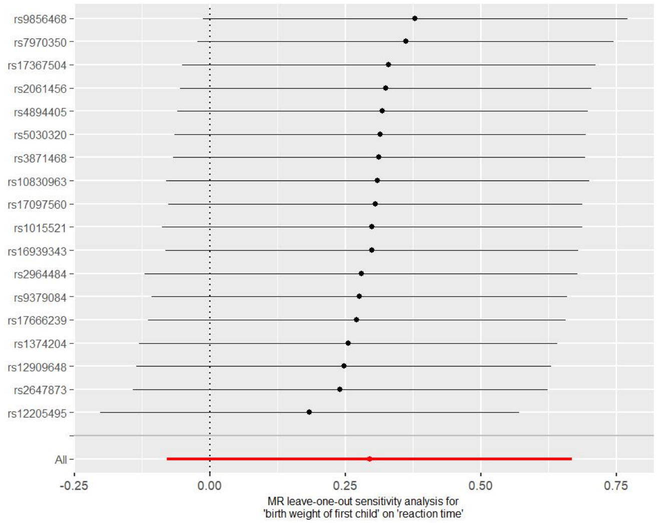
rs9856468
rs7970350
rs17367504
rs2061456
rs4894405
rs5030320
rs3871468
rs10830963
rs17097560
rs1015521
rs16939343
rs2964484
rs9379084
rs17666239
rs1374204
rs12909648
rs2647873
rs12205495
All
-0.25
0.00
0.25
0.50
0.75
MR leave-one-out sensitivity analysis for
'birth weight of first child' on 'reaction time'

Exposure: Number of live births

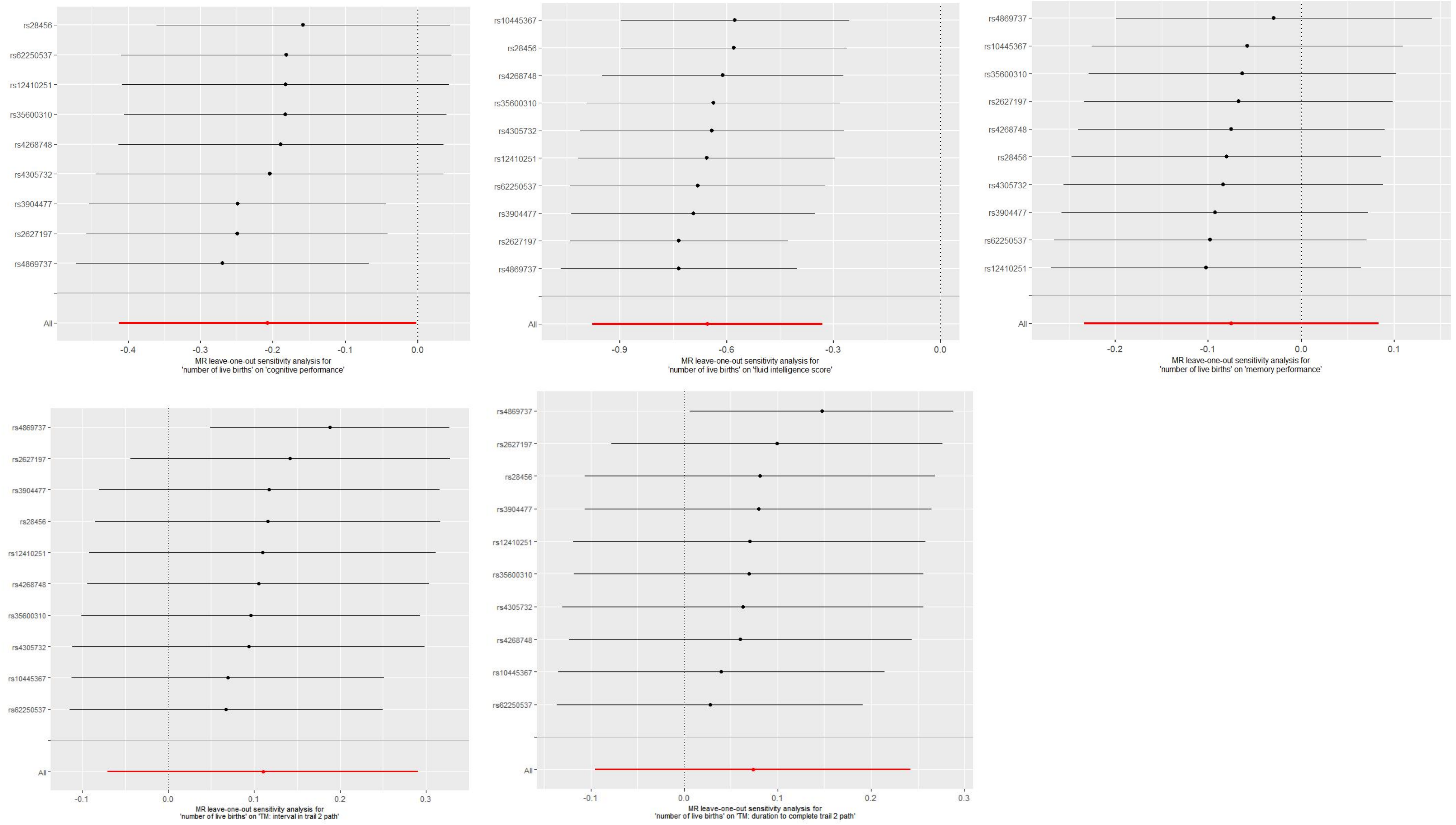

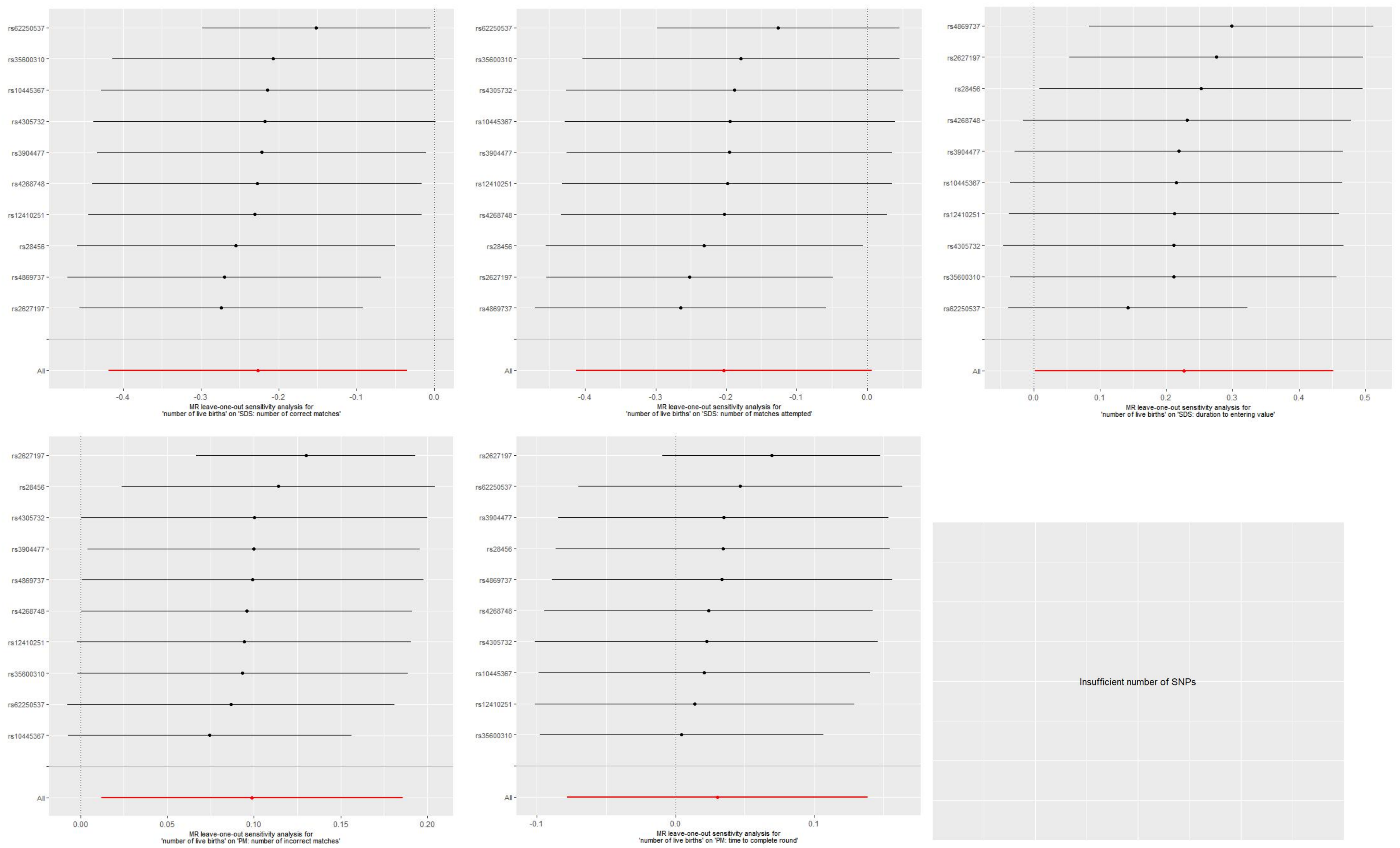
rs62250537
rs35600310
rs10445367
rs4305732
rs3904477
rs4268748
rs12410251
rs28456
rs4869737
rs2627197
All
MR leave-one-out sensitivity analysis for
'number of live births' on 'SDS: number of correct matches'
rs62250537
rs35600310
rs4305732
rs10445367
rs3904477
rs12410251
rs4268748
rs28456
rs2627197
rs4869737
All
MR leave-one-out sensitivity analysis for
'number of live births' on 'SDS: number of matches attempted'
rs4869737
rs2627197
rs28456
rs4268748
rs3904477
rs10445367
rs12410251
rs4305732
rs35600310
rs62250537
All
MR leave-one-out sensitivity analysis for
'number of live births' on 'SDS: duration to entering value'
rs2627197
rs28456
rs4305732
rs3904477
rs4869737
rs4268748
rs12410251
rs35600310
rs62250537
rs10445367
All
MR leave-one-out sensitivity analysis for
'number of live births' on 'PM: number of incorrect matches'
rs2627197
rs62250537
rs3904477
rs28456
rs4869737
rs4268748
rs4305732
rs10445367
rs12410251
rs35600310
All
MR leave-one-out sensitivity analysis for
'number of live births' on 'PM: time to complete round'
Insufficient number of SNPs

Exposure: Medical abortion

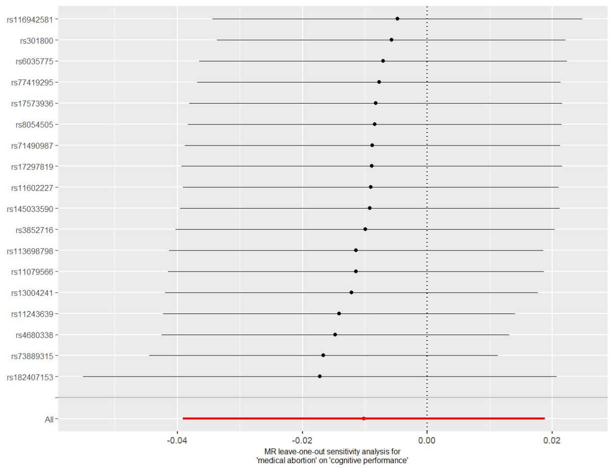

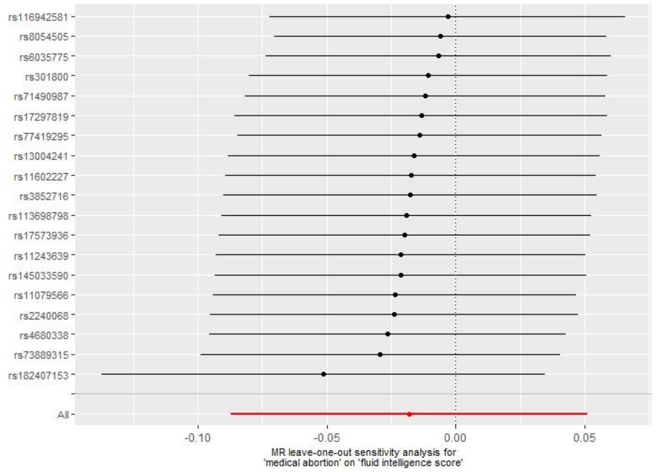

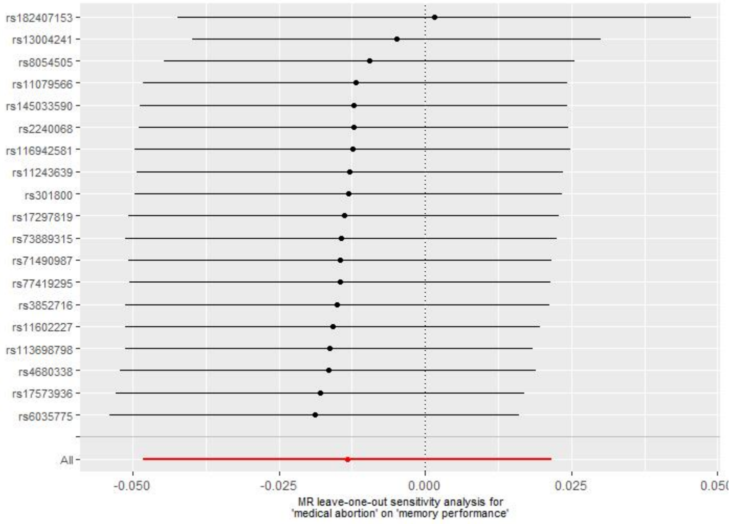

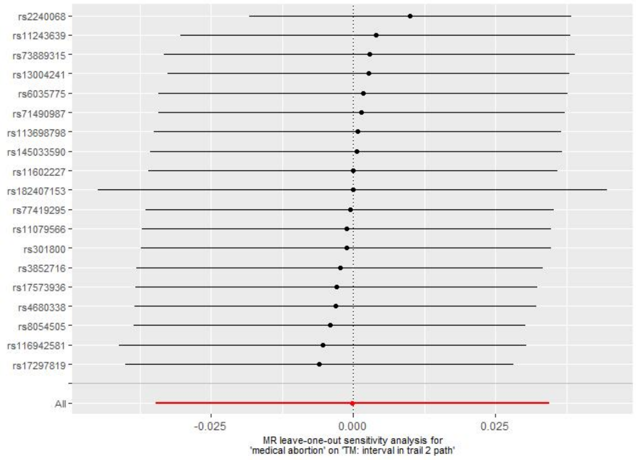

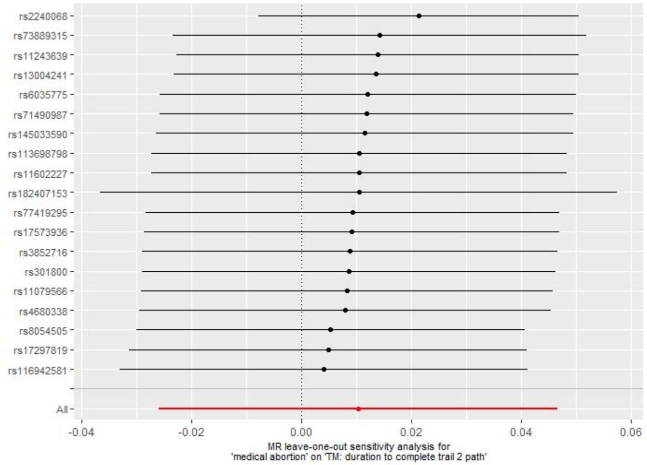

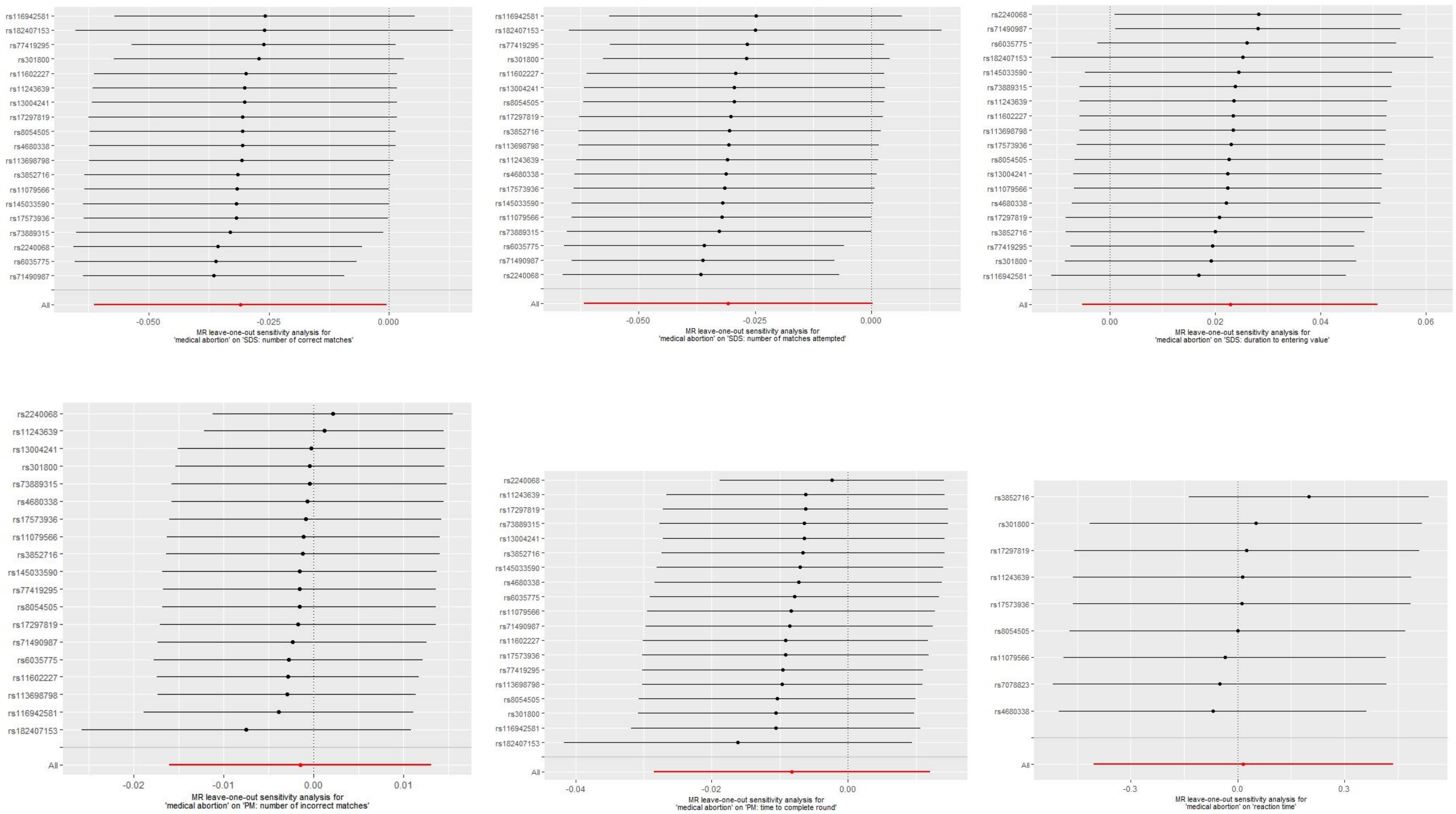
MR leave-one-out sensitivity analysis for
'medical abortion' on 'SDS: number of correct matches'
MR leave-one-out sensitivity analysis for
'medical abortion' on 'SDS: number of matches attempted'
MR leave-one-out sensitivity analysis for
'medical abortion' on 'SDS: duration to entering value'
MR leave-one-out sensitivity analysis for
'medical abortion' on 'PM: number of incorrect matches'
MR leave-one-out sensitivity analysis for
'medical abortion' on 'PM: time to complete round'
MR leave-one-out sensitivity analysis for
'medical abortion' on 'reaction time'

Exposure: Ever taken oral contraceptive pill

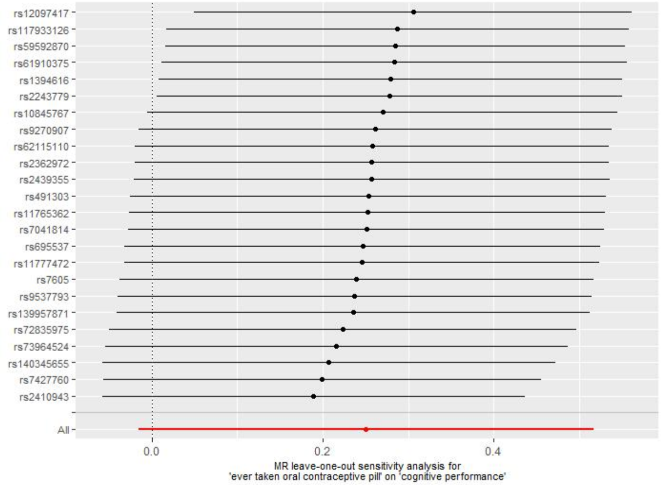

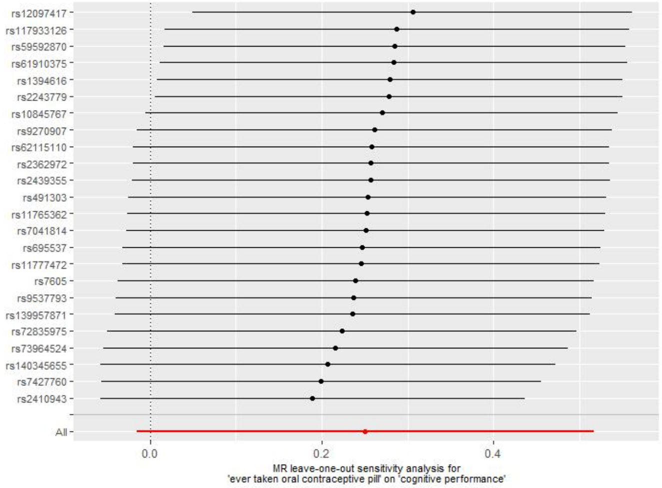

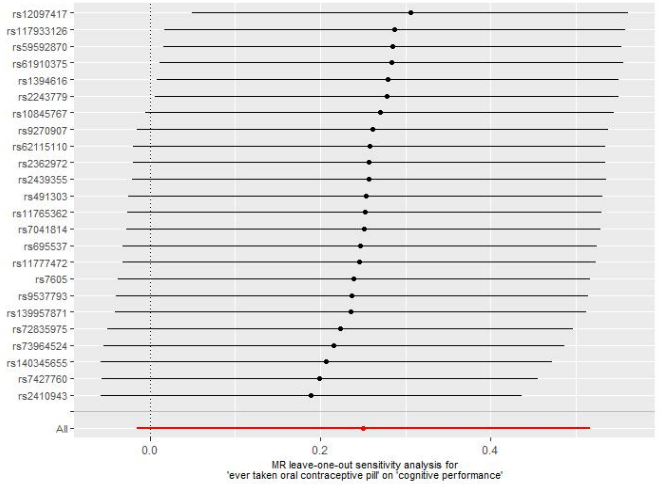

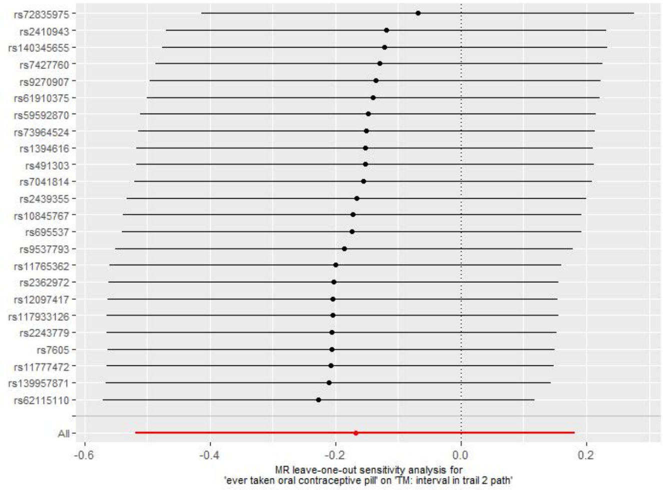

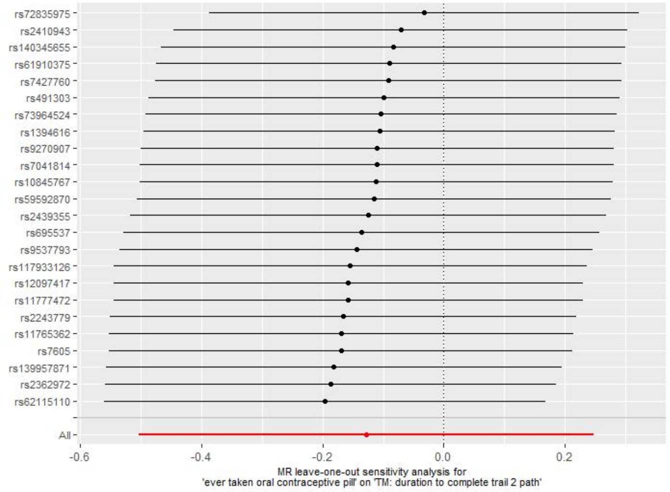

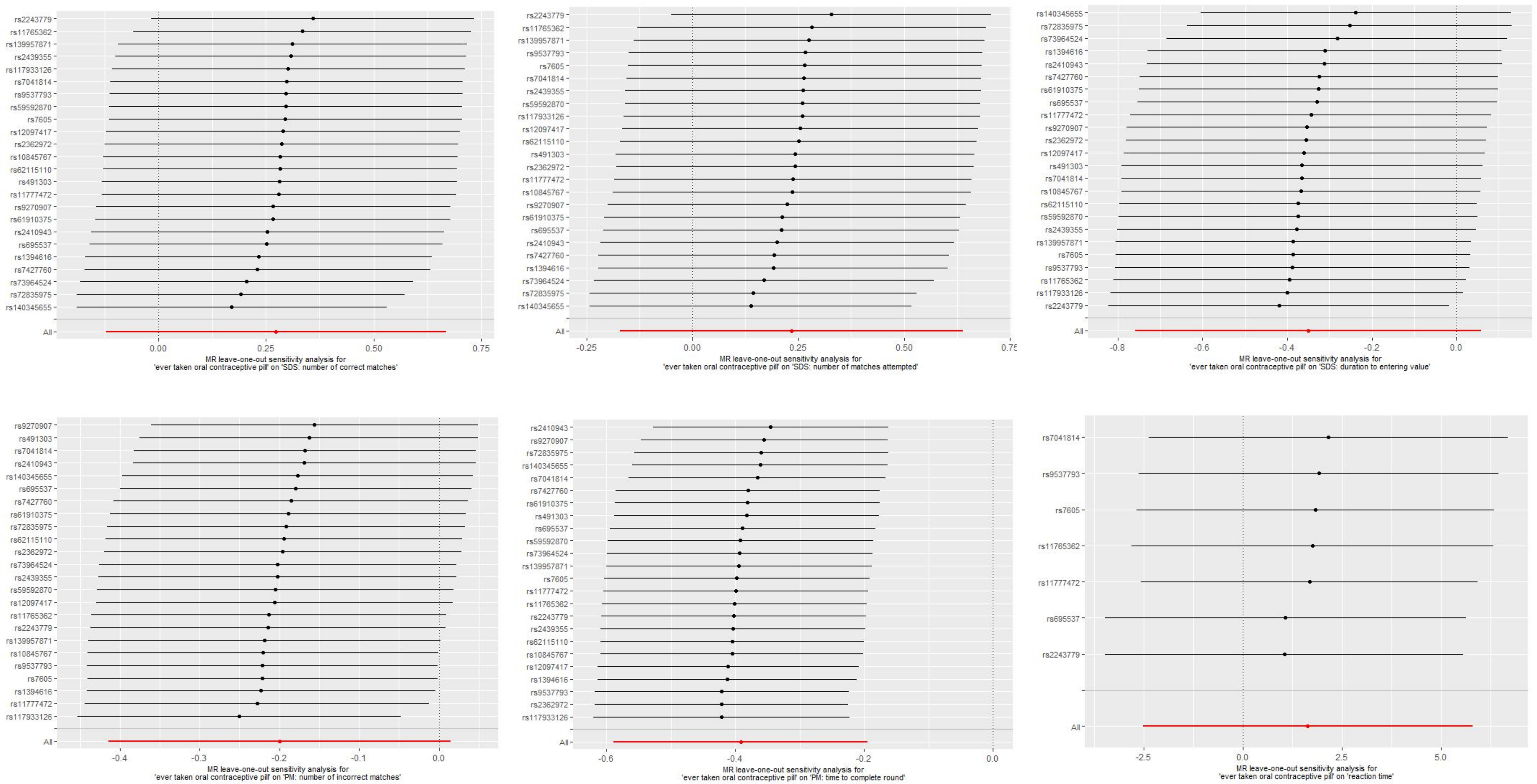

MR leave-one-out sensitivity analysis for
'ever taken oral contraceptive pill' on 'SDS: number of correct matches'
MR leave-one-out sensitivity analysis for
'ever taken oral contraceptive pill' on 'SDS: number of matches attempted'
MR leave-one-out sensitivity analysis for
'ever taken oral contraceptive pill' on 'SDS: duration to entering value'
MR leave-one-out sensitivity analysis for
'ever taken oral contraceptive pill' on 'PM: number of incorrect matches'
MR leave-one-out sensitivity analysis for
'ever taken oral contraceptive pill' on 'PM: time to complete round'
MR leave-one-out sensitivity analysis for
'ever taken oral contraceptive pill' on 'reaction time'

Exposure: Ever used hormone-replacement therapy

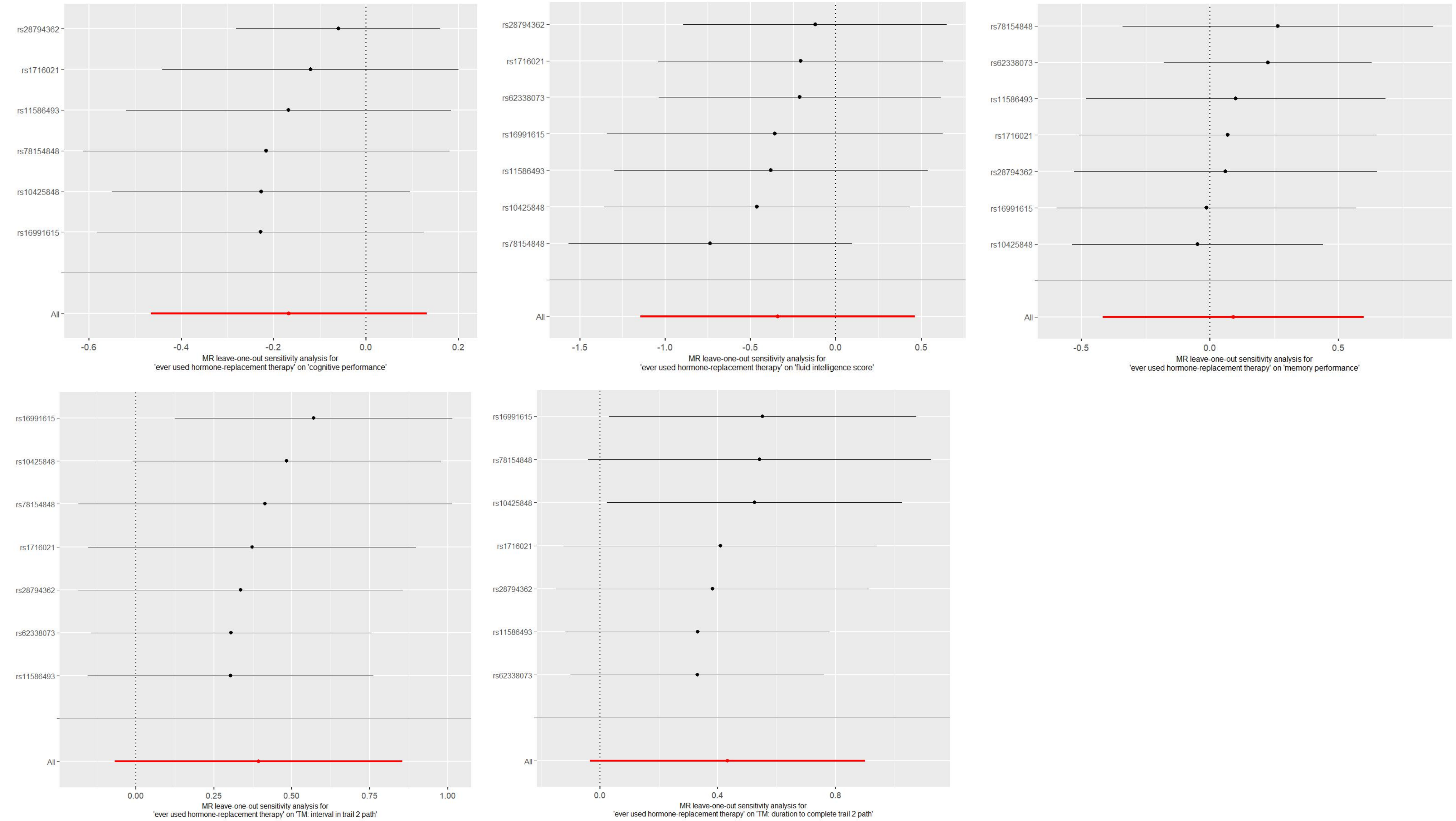

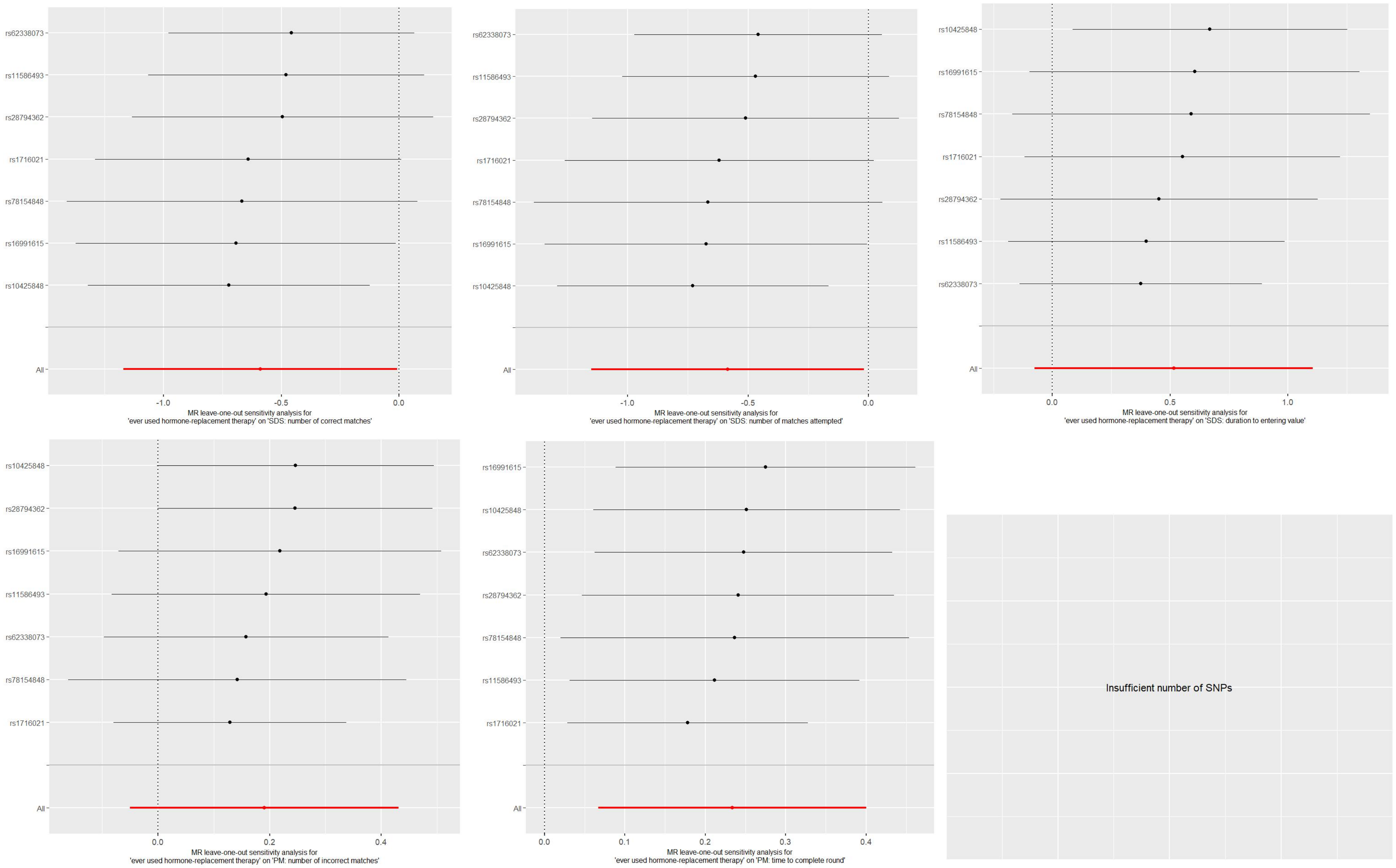
rs62338073
rs11586493
rs28794362
rs1716021
rs78154848
rs16991615
rs10425848
All
-1.0
-0.5
0.0
MR leave-one-out sensitivity analysis for
'ever used hormone-replacement therapy' on 'SDS: number of correct matches'
rs62338073
rs11586493
rs28794362
rs1716021
rs78154848
rs16991615
rs10425848
All
-1.0
-0.5
0.0
MR leave-one-out sensitivity analysis for
'ever used hormone-replacement therapy' on 'SDS: number of matches attempted'
rs10425848
rs16991615
rs78154848
rs1716021
rs28794362
rs11586493
rs62338073
All
0.0
0.5
1.0
MR leave-one-out sensitivity analysis for
'ever used hormone-replacement therapy' on 'SDS: duration to entering value'
rs10425848
rs28794362
rs16991615
rs11586493
rs62338073
rs78154848
rs1716021
All
0.0
0.2
0.4
MR leave-one-out sensitivity analysis for
'ever used hormone-replacement therapy' on 'PM: number of incorrect matches'
rs16991615
rs10425848
rs62338073
rs28794362
rs78154848
rs11586493
rs1716021
All
0.0
0.1
0.2
0.3
0.4
MR leave-one-out sensitivity analysis for
'ever used hormone-replacement therapy' on 'PM: time to complete round'
Insufficient number of SNPs

Adjustment	Methods	Beta (95%CI)	P
**MVMR for the effect of HRT use on cognitive function**			
**Controlled for early menopause**			
SDS: Number of correct matches	IVW	-0.634 (-1.279 to 0.012)	0.054
SDS: Number of matches attempted	IVW	-0.614 (-1.230 to 0.002)	0.051
PM: Time to complete round	IVW	0.311 (-0.137 to 0.760)	0.174
**Controlled for early menopause and premature ovarian insufficiency**			
SDS: Number of correct matches	IVW	-0.829 (-1.607 to 0.011)	0.056
SDS: Number of matches attempted	IVW	-0.775 (-1.560 to 0.010)	0.053
PM: Time to complete round	IVW	0.151 (-0.388 to 0.689)	0.584
**Controlled for early menopause, premature ovarian insufficiency, age at HRT initiation and duration of use**			
SDS: Number of correct matches	IVW	-1.050 (-2.007 to 0.001)	0.052
SDS: Number of matches attempted	IVW	-0.957 (-1.914 to 0.001)	0.051
PM: Time to complete round	IVW	0.232 (-0.322 to 0.786)	0.412

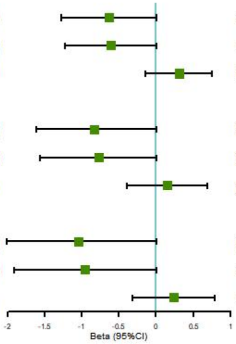

Figure S5. MVMR analyses of statistically significant HRT use and cognitive function tests in the UVMR, after adjustment for early menopause, premature ovarian insufficiency, age at HRT initiation and duration of use.

Reproductive traits	Methods	Beta (95%CI)	P
**Controlled for total breastfeeding duration**			
**Age at first birth**			
Cognitve performance	IVW	0.104 (0.069 to 0.139)	<0.001
Fluid intelligence score (FIS)	IVW	0.217 (0.143 to 0.291)	<0.001
Memory performance	IVW	0.015 (-0.018 to 0.049)	0.375
TM: Interval in trail 2 path	IVW	-0.049 (-0.080 to -0.019)	0.002
TM: Duration to complete trail 2 path	IVW	-0.041 (-0.072 to -0.009)	0.012
SDS: Number of correct matches	IVW	0.047 (0.020 to 0.074)	<0.001
SDS: Number of matches attempted	IVW	0.042 (0.013 to 0.072)	0.004
SDS: Duration to entering value	IVW	-0.042 (-0.070 to -0.014)	0.003
**Number of live births**			
Cognitve performance	IVW	-0.204 (-0.373 to -0.036)	0.017
Fluid intelligence score (FIS)	IVW	-0.683 (-1.092 to -0.274)	0.001
SDS: Number of correct matches	IVW	-0.220 (-0.403 to -0.037)	0.018
SDS: Duration to entering value	IVW	0.223 (0.011 to 0.436)	0.039
PM: Number of incorrect matches	IVW	0.101 (-0.006 to 0.207)	0.064

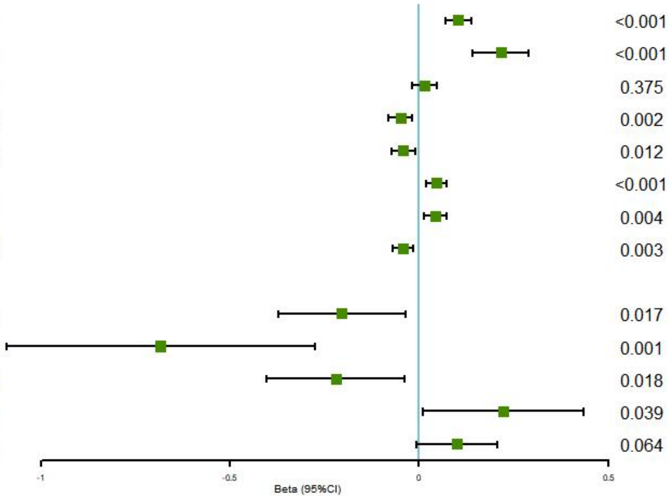

Figure S6. MVMR analyses of statistically significant age at first birth/number of live births and cognitive function tests in the UVMR, after adjustment for total breastfeeding duration.

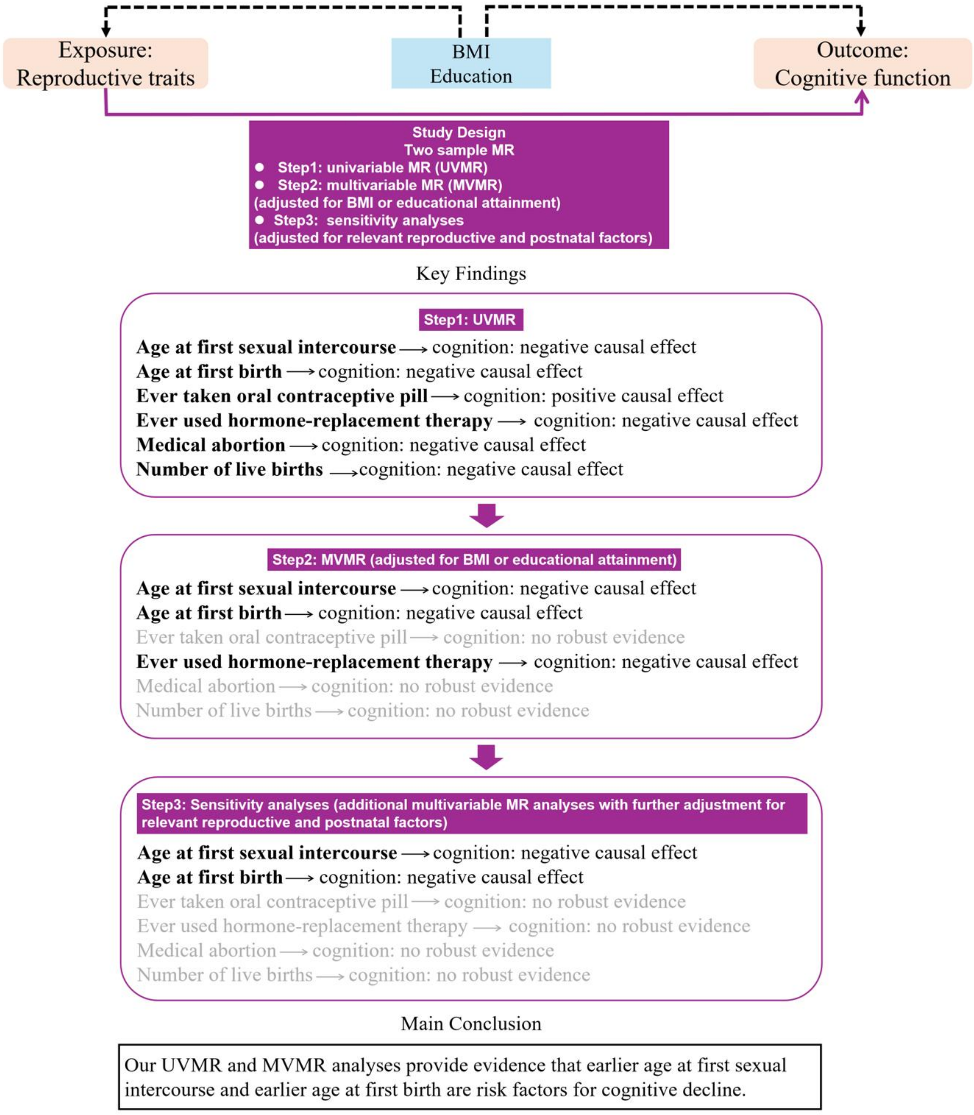

Figure S7. Study workflow and summary of key results from Mendelian randomization analyses.
